# Halogenoborane mediated allene cyclooligomerization†
†Electronic supplementary information (ESI) available: Additional experimental details, further spectral and crystallographic data. CCDC 1862533–1862535 and 1881527. For ESI and crystallographic data in CIF or other electronic format see DOI: 10.1039/c8sc04790a


**DOI:** 10.1039/c8sc04790a

**Published:** 2019-01-02

**Authors:** Xin Tao, Christian Wölke, Constantin G. Daniliuc, Gerald Kehr, Gerhard Erker

**Affiliations:** a Organisch-Chemisches Institut , Westfälische Wilhelms-Universität Münster , Corrensstraße 40 , 48149 Münster , Germany . Email: erker@uni-muenster.de

## Abstract

The halogenoboranes XB(C_6_F_5_)_2_ (X: Cl, Br) react with allene to give a mixture of the cyclotrimer 1,3,5-trimethylenecyclohexane (**1**) and the related halogenoborylated cyclotetramerization products **3a** and **3b**. Alkyl-substituted allenes were catalytically cyclotrimerized metal-free by XB(C_6_F_5_)_2_ (X: Cl, Br) to give *cis*,*trans*-2,4,6-trialkyl-1,3,5-trimethylenecyclohexanes under mild conditions.

## Abstract 2

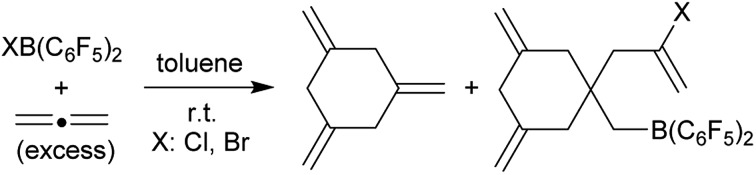
Allene reacts with the strongly electrophilic halogenoboranes XB(C_6_F_5_)_2_ (X: Cl or Br) by forming a mixture of 1,3,5-trimethylenecyclohexane and the stoichiometric halogeno-borated tetramerization products.

Allenes serve as important organic building blocks.^1^ The parent allene, propadiene, has been cyclotrimerized at a variety of transition metal catalysts, but this usually gave predominately 1,2,4-trimethylenecyclohexane and only minor amounts of the 1,3,5-trimethylenecyclohexane isomer **1**.^2^,^3^ Compound **1** had been synthesized in stoichiometric reaction sequences.^4^ We recently developed the metal-free, HB(C_6_F_5_)_2_ catalysed formation of isomerically pure **1** by allene cyclotrimerization under mild conditions.^5^ We also had recently shown that a small series of 1-alkynes could be converted to linear oligomers by treatment with either of the halogenoboranes XB(C_6_F_5_)_2_**2a** (X: Cl) or **2b** (X: Br). The resulting products contained a halide substituent at one end of the oligoacetylene chain and the B(C_6_F_5_)_2_ functional group at the other.^6^ This prompted us to investigate which type of a reaction allene would undergo with either of the reagents **2a** and **b**. We have performed these reactions and here report about their surprising outcome.

We first carried out the reaction of excess allene with ClB(C_6_F_5_)_2_ (**2a**) in d_8_-toluene in a Young NMR tube at room temperature. After 7 h reaction time we monitored the NMR features of a mixture that contained the products 1,3,5-trimethylenecyclohexane (**1**) and the chloroborylated allene tetramer **3a** in a *ca.* 1 : 1 ratio (both present in *ca.* 8 mol% in the mixture) plus unreacted **2a** (*ca.* 10 mol%) and allene (*ca.* 75 mol%). After 24 h the amount of the pair of allene cyclooligomerization products had almost doubled and there remained only *ca.* 3 mol% of the chloroborane **2a**. That was almost completely consumed after 48 h reaction time at room temperature. The products **1** and **3a** were identified spectroscopically from the mixture [**1**: *δ* 4.53, 2.70 (^1^H)]. Compound **3a** shows the typical olefinic exo-methylene pairs of ^1^H NMR resonances at *δ* 5.08, 4.84 (9-CH_2_=) and *δ* 4.63, 4.48 (4,6-CH_2_=) as well as the AX-spin system of the diastereotopic 3,7-CH_2_ pairs (*δ* 2.24, 2.00, ^2^*J*_HH_ = 14.0 Hz) and the AB pattern of the 5-CH_2_ at *δ* 2.58, 2.54. The 1-CH_2_ group at boron and the 8-CH_2_ unit give rise to ^1^H NMR signals at *δ* 2.18 and *δ* 2.26, respectively (atom numbering scheme analogous to Fig. 1). Compound **3a** shows a broad ^11^B NMR resonance at *δ* 71.5, which is typical for a planar tri-coordinate Lewis acidic borane in this situation (Δ^19^*F*_m,p_ = 13.2 ppm, for details see the ESI†).

**Fig. 1:**
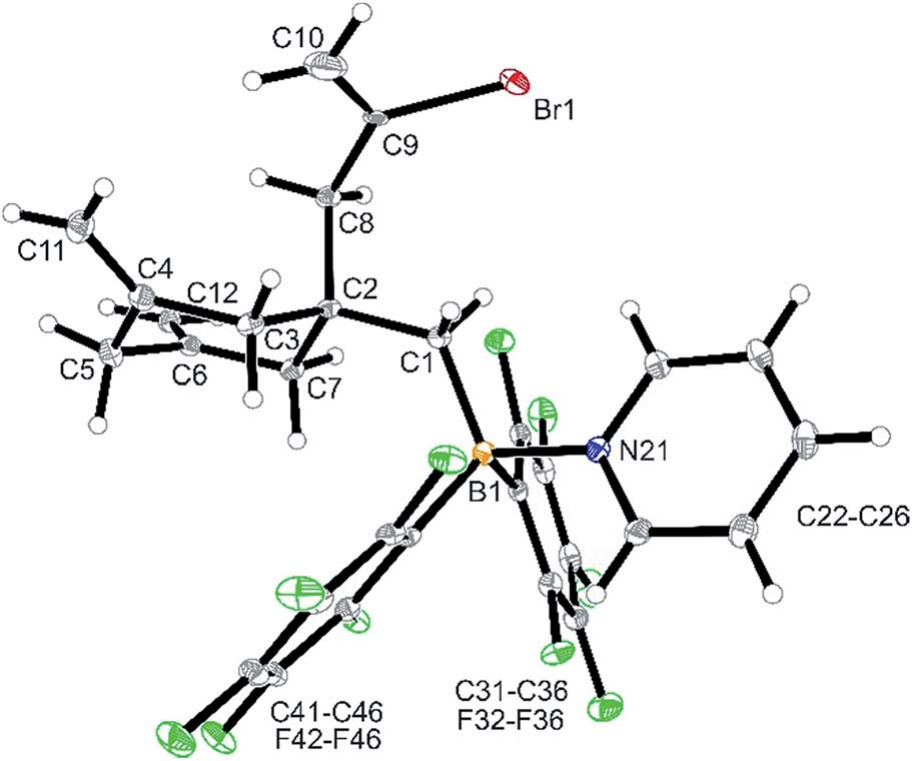
A view of the molecular structure of compound **4b**. Selected bond lengths (Å) and angles (°): B1–C1 1.634(2) B1–N21 1.661(2) C4–C11 1.323 (2) C6–C12 1.327(2) C9–C10 1.351(9) C9–Br1 1.910(5) B1–C1–C2 124.4(1) C1–C2–C8 108.5(1) C2–C8–C9 116.3(3) C8–C9–C10 124.5(6) C8–C9–Br1 118.4(4).

The reaction of allene with BrB(C_6_F_5_)_2_ (**2b**) was carried out analogously. The reaction was directly followed by NMR spectroscopy. It resulted in the formation of the products **1** and **3b** in a *ca.* 1 : 2 ratio (4 h, r.t.). Compound **3b** was characterized from the mixture by NMR spectroscopy. It shows similar spectra as its chloro-substituted analogue **3a** (see the ESI† for details and the depicted spectra).

We prepared compound **3a** on a preparative scale. After direct treatment with pyridine we isolated the Lewis adduct **4a** as a white solid in 53% yield (Scheme 1). It was characterized by C, H, N elemental analysis, by spectroscopy and by X-ray diffraction (see the ESI† for details). It showed very similar parameters as the analogous bromine containing compound **4b** (see below).

**Scheme 1:**
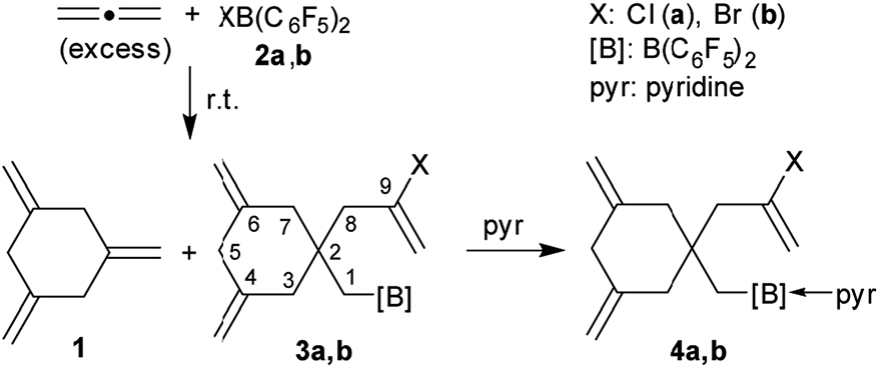
Reaction of allene with the halogenoboranes **2a** and **2b**.

Treatment of *in situ* prepared **3b** with a slight excess of pyridine gave the adduct **4b**, which we isolated in 72% yield as a white solid on a 100 mg scale. The X-ray crystal structure analysis (Fig. 1) showed the central six-membered ring that was constructed by connecting three allene units. It features the exo-methylene C=C double bonds annulated at carbon atoms C4 and C6 and it has the CH_2_–CBr=CH_2_ unit, derived from the fourth connected allene unit, attached at the ring carbon atom C2. This carbon atom also bears the CH_2_–B(C_6_F_5_)_2_ substituent, which has the pyridine donor added to its boron atom. The central six-membered carbocycle of compound **4b** features a distorted chair-like ring conformation.

In solution (CD_2_Cl_2_) compound **4b** shows the ^1^H NMR =CH_2_ signals of the symmetry equivalent pair of exo-methylene groups at the ring carbons C4 and C6 and the pair of signals of the C9=CH_2_ moiety. The C3/C7 CH_2_ hydrogen atoms are pairwise diastereotopic and the C8 and C1 CH_2_ groups both show ^1^H NMR singlets. Compound **4b** shows a ^11^B NMR resonance in the typical tetra-coordinated borane range at *δ* –1.4 (Δ^19^*F*_m,p_ = 5.6 ppm).

It is probably reasonable to assume that our reaction sequence starts with a halogenoboration reaction^7^ of allene (Scheme 2). This would generate the allylborane **5** that probably undergoes an allylboration^8^ reaction with a second equivalent of allene to form the intermediate **6**. Since this contains an allylborane subunit it can undergo another allylboration reaction to give **7**. This sequence could in principle be propagated to form a respective series of linear borylated allene oligomers **7**, **8***etc.*, were there not the attractive possibility of these systems to undergo alternative intramolecular allylboration.^9^ In the case of **7** this would lead to the cyclization product **9**. Subsequent XB(C_6_F_5_)_2_ elimination provides an attractive pathway to the observed product 1,3,5-trimethylenecyclohexane (**1**). This would in principle constitute a cyclotrimerization of allene catalysed by the XB(C_6_F_5_)_2_ reagents. However, the intramolecular allylboration of **8**, giving the other experimentally observed products **3**, represents a competing stoichiometric reaction branch that eventually removes the XB(C_6_F_5_)_2_ reagent from the system (Scheme 2) and thus terminates the catalytic sequence of the formation of **1**.

**Scheme 2:**
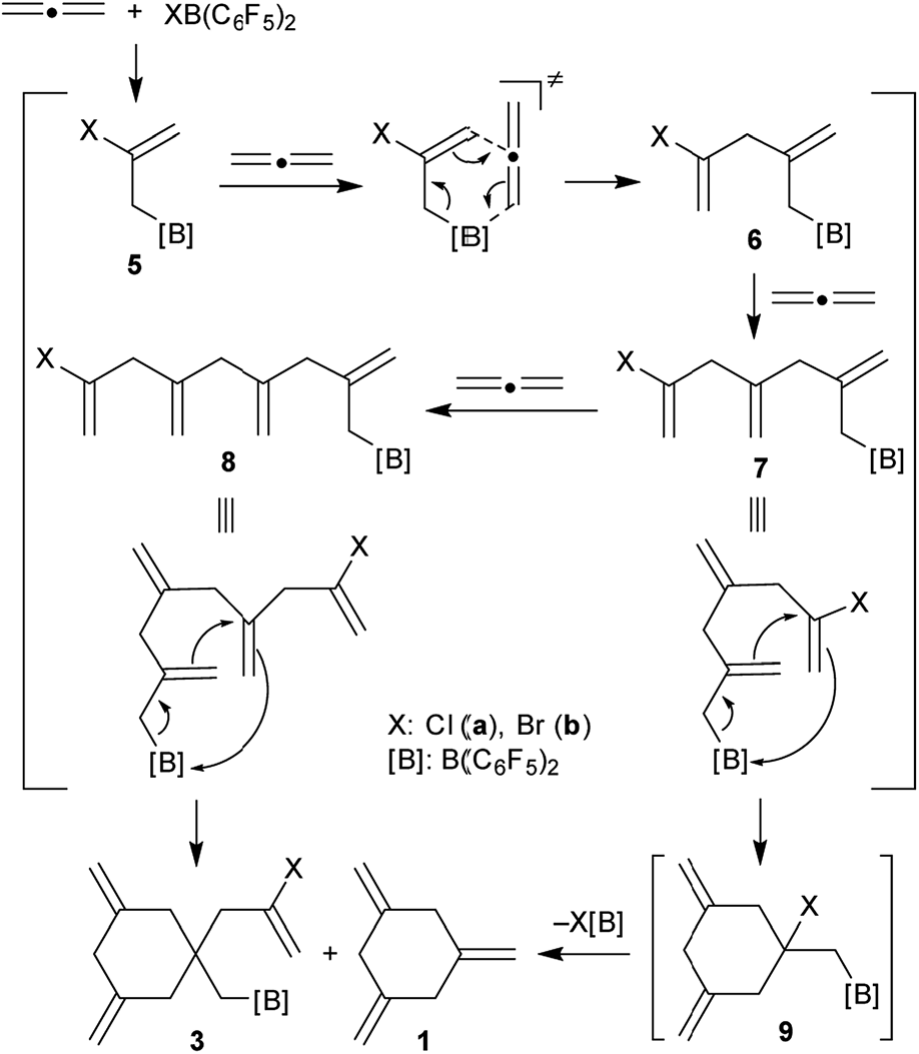
Reaction pathway for the XB(C_6_F_5_)_2_ mediated allene cyclooligomerization.

Compounds **3a,b** are reactive boron Lewis acids. With the bulky ^*t*^Bu_3_P Lewis base (**10**) they react by ring closure^10^,^11^ to eventually give the zwitterionic product **12** (Scheme 3). As a typical example, the reaction of the *in situ* generated chloride containing derivative **3a** was carried out with a *ca.* two molar equivalents of ^*t*^Bu_3_P in toluene (24 h, 60 °C). Workup with pentane and dichloromethane in this case gave a *ca.* 79 : 21 mixture of compound **12** (characterization see below) and the [^*t*^Bu_3_PH^+^]Cl^–^ phosphonium salt **11a** (^31^P NMR: *δ* 47.9, ^1^*J*_PH_ ∼463 Hz, some of the stoichiometric by-product **11a** was probably lost during the workup procedure).

**Scheme 3:**
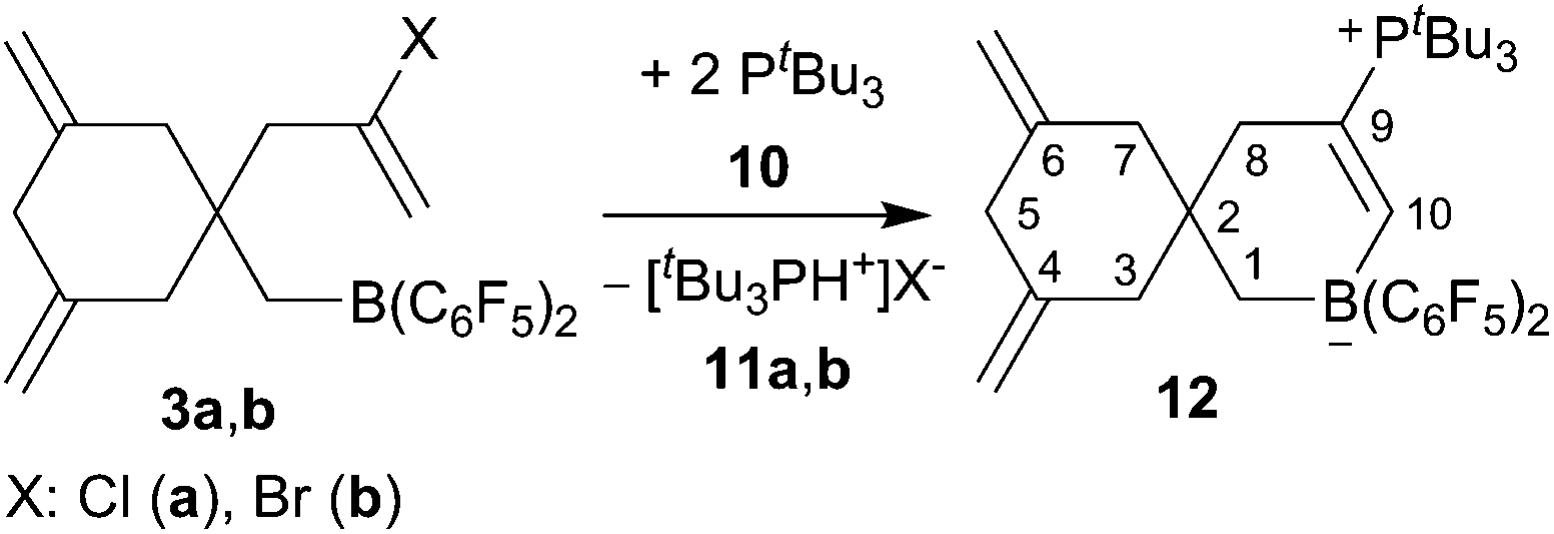
Reaction of the products **3** with two molar equiv. of ^*t*^Bu_3_P.

The reaction between the more reactive borane **3b** and ^*t*^Bu_3_P was carried out similarly (toluene, 24 h, r.t.). Workup in this case gave the pure compound **12** as a white solid, which we isolated in 65% yield. It was characterized by C, H, N elemental analysis, by spectroscopy and by X-ray diffraction (single crystals of compound **12** were obtained from dichloromethane/pentane by the diffusion method). The X-ray crystal structure analysis shows the newly formed borataspiro[5,5]undecene framework (Fig. 2) which was selectively formed by boron induced allene tetramerization followed by phosphane induced ring closure reaction. The zwitterionic compound has the bulky ^*t*^Bu_3_P-substituent attached at the C(9)=C(10) carbon–carbon double bond. The adjacent six-membered ring shows carbon atoms C4 and C6 which serve both as the ring sp^2^-carbons of the pair of exo-methylene C=CH_2_ groups.

**Fig. 2:**
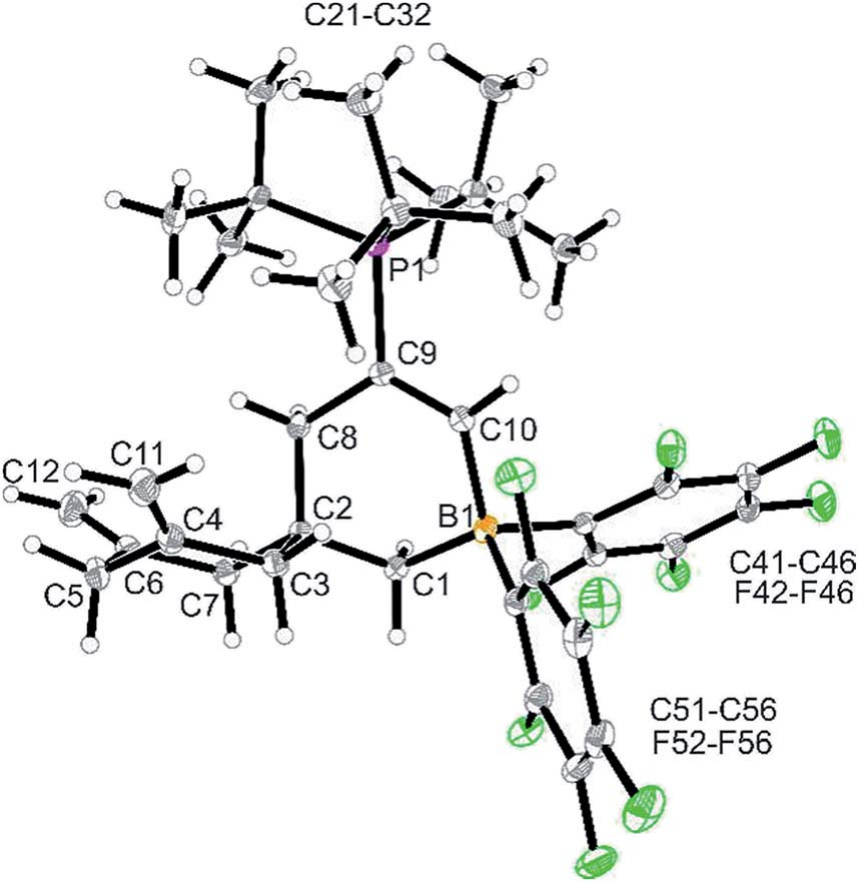
A view of the molecular structure of the zwitterionic FLP product **12**. Selected bond lengths (Å) and angles (°): B1–C1 1.630(4) B1–C10 1.624(4) C4–C11 1.328(4) C6–C12 1.323(5) C9–C10 1.343(4) C9–P1 1.834(3) B1–C1–C2 113.5(2) C1–C2–C8 108.5(2) C1–B1–C10 107.0(2) C2–C2–C7 108.0(2) C2–C8–C9 114.0(2) C8–C9–C10 121.4(2) C9–C10–B1 125.7(3).

In solution (CD_2_Cl_2_) compound **12** shows the typical ^1^H NMR doublet of the ^*t*^Bu_3_P-substituent. It features the =CH signals of the newly formed internal olefinic moiety at *δ* 8.21 [^1^H: d, ^3^*J*_PH_ = 27.8 Hz; ^13^C: *δ* 177.0 (broad)]. The BCH_2_ group shows up as a ^1^H NMR signal at *δ* 1.20 and the C(8)H_2_ methylene group as a doublet at *δ* 2.06 (^3^*J*_PH_ = 4.5 Hz). The pair of olefinic exomethylene groups of the adjacent carbocyclic six-membered ring shows typical ^1^H NMR signals at *δ* 4.61/4.41 and the resonances of the pairwise diastereotopic methylene hydrogen atoms at the C3/C7 pair and at C5 (see the ESI† for details).

We also reacted a pair of alkyl-substituted allenes with the halogenoboranes **2** and found a marked dependence of the overall reaction pathway on the substitution pattern. In the two cases investigated the competition between the two previously observed pathways, namely catalytic cyclotrimerization and stoichiometric formation of a tetramer derivative, was shifted to the catalytic side.

We treated *n*-octylallene (**13c**), which we prepared according to a procedure reported by Ma *et al.*^12^ with 10 mol% of ClB(C_6_F_5_)_2_ (**2a**) in d_8_-toluene solution at 60 °C. Workup after 48 h reaction time involving purification by chromatography gave the cyclotrimer **14c** as the major product (Scheme 4). Compound **14c** was isolated in 44% yield as a colorless oil. The NMR spectra showed the symmetry features of the *cis*,*trans*-2,4,6-trialkyl-substituted 1,3,5-trimethylenecyclohexane diastereomer. It features the ^1^H NMR triplets of the 2,6-CH and the 4-CH ring hydrogens in a 2 : 1 intensity ratio. The 1-H_2_C= unit shows one ^1^H NMR resonance, whereas the 3,5-H_2_C= moieties showed two due to their stereochemically unsymmetrical environment (see the ESI† for further details). The BrB(C_6_F_5_)_2_ borane is an equally efficient metal-free cyclotrimerization catalyst for the *n*-octylallene **13c**. The catalytic reaction was carried out under analogous conditions and gave the product **14c** in 46% yield after workup (some minor byproduct was isolated (*ca.* 20%) but not positively identified as yet). We performed the XB(C_6_F_5_)_2_ catalysed alkylallene cyclotrimerization reaction for a second example: the reaction of *n*-dodecylallene (**13d**) with either of the Cl/BrB(C_6_F_5_)_2_ borane (10 mol%, 60 °C, 48 h in d_8_-toluene) gave the tri-substituted cyclotrimer **14d** with *cis*,*trans*-attachment of the long chained alkyl groups as the major product. We isolated it as a colorless oil in 40% yield from the ClB(C_6_F_5_)_2_ catalysed reaction [54% with BrB(C_6_F_5_)_2_] (see the ESI† for the characterization of **14d**).

**Scheme 4:**
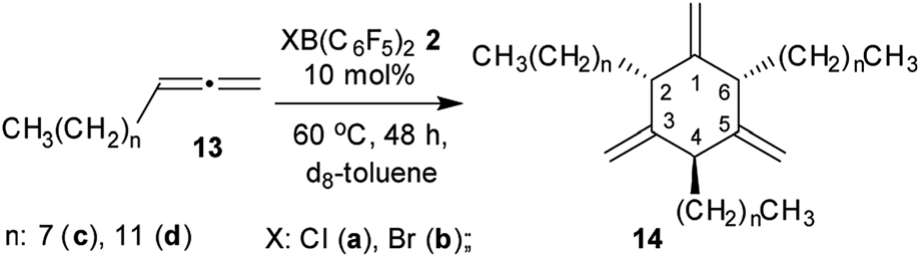
XB(C_6_F_5_)_2_ (X: Cl, Br) catalyzed cyclotrimerization of alkyl substituted allenes.

We had previously shown that HB(C_6_F_5_)_2_ serves as an efficient metal-free catalyst for the cyclotrimerization of allene to 1,3,5-trimethylenecyclohexane and of cyclohexylallene to *cis*,*trans*-2,4,6-tricyclohexyl-1,3,5-trimethylenecyclohexane.^5^ Our present study shows that the strongly electrophilic halogenoboranes XB(C_6_F_5_)_2_ (X: Cl, Br) in principle can induce the same catalytic reaction. Both these strongly electrophilic halogeno-boranes serve as catalysts for the cyclotrimerization of long-chain *n*-alkylallenes **13c,d** to give the respective cyclotrimers **14c,d** as the major products. Ring-closure and elimination is sufficiently effective to close the catalytic cycle with liberation of the respective XB(C_6_F_5_)_2_ (X: Cl, Br) catalyst (Scheme 2). However, the reaction seems to be sensitive to sterics in the competition between ring-closure and further allene incorporation. In the case of the unsubstituted parent allene substrate we found a substantially competing further oligomerization step which eventually leads to the formation of the stoichiometric products **3a** and **3b**, thereby consuming the borane catalyst of the allene cyclotrimerization reaction and, thus, leading to termination of the catalytic reaction in this specific case. Nevertheless, our study has shown that allene oligomerization beyond trimerization is possible with such borane systems. This makes us hopeful that it might be possible to open novel ways of utilization of allenes by a further development of metal-free reactions along the characteristic lines that have shown up in this study.

We have started to use the allene cyclotrimerization products **1** and **14** as the starting materials for the conversion to the respective arene isomers. It is known that 1,3,5-trimethylene-cyclohexane is resistant to thermal isomerization, but it was reported that it could be isomerized by treatment with acid.2a,^13^ We repeated this reaction: treatment of **1** with 5 mol% of *p*-toluene sulfonic acid in toluene solution at r.t. for 1.5 h cleanly converted **1** to mesitylene (Scheme 5). We also treated the *n*-octyl- and cyclohexylallene cyclotrimers **14c**^14^ and **14e**^5^ with *p*-toluene sulfonic acid. Compound **14c** was fully converted on a preparative scale with 10 mol% of the acid catalyst during 48 h at r.t. The hexa-substituted arene **15c** was isolated as an oil in 78% after workup. It was characterized by ^1^H and ^13^C NMR spectroscopy (see the ESI† for details). Compound **14e** needed slightly more vigorous conditions. It required treatment with 10 mol% of the *p*-toluene sulfonic acid catalyst at 80 °C for 5 h to become converted. This reaction is not overly selective, but we isolated the aromatic isomer **15e** in 37% yield from the reaction mixture. The compound was characterized by spectroscopy and by an X-ray crystal structure analysis (see the ESI† for details). In principle, these reactions have shown that the metal-free X–B(C_6_F_5_)_2_ catalyzed allene cyclotrimerization opens attractive pathways to the synthesis of interesting highly substituted arene products.

**Scheme 5:**
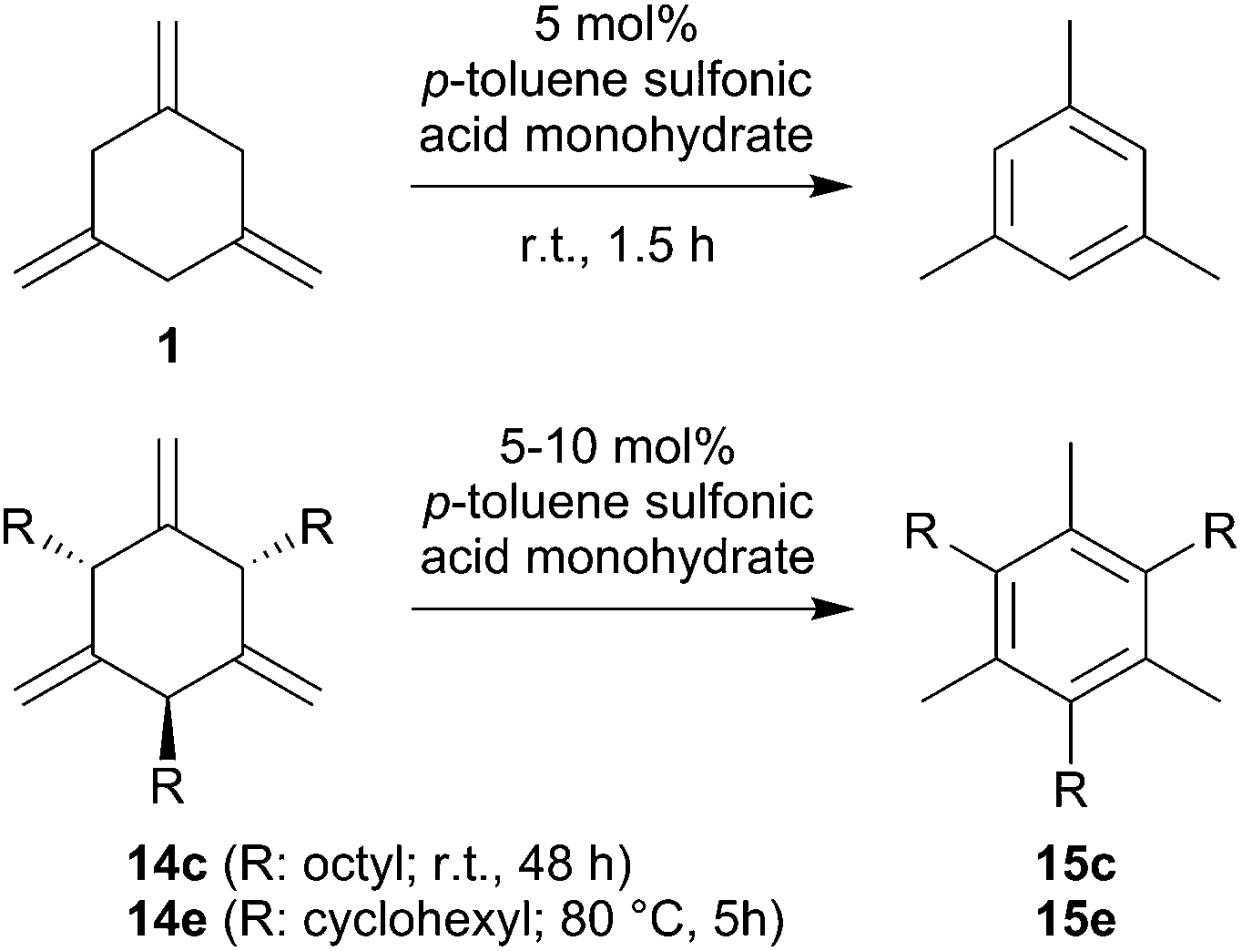
Acid catalyzed isomerization reactions of allene-cyclotrimers to mesitylene derivatives.

## Conflicts of interest

There are no conflicts to declare.

## Supplementary Material

Supplementary information

Crystal structure data
