# Supplementary material for: Halogenoborane mediated allene cyclooligomerization
Source: Chem Sci. 2019 Jan 2;10(8):2478–82. doi: 10.1039/c8sc04790a (PMC6385850; doi:10.1039/c8sc04790a)
Supplement: Supplementary file 1 [file SC-010-C8SC04790A-s001.pdf]

Electronic Supplementary Material (ESI) for Chemical Science.
This journal is © The Royal Society of Chemistry 2019

Halogenoborane mediated allene cyclooligomerization

Xin Tao, Christian Wölke, Constantin G. Daniliuc, Gerald Kehr, Gerhard Erker

Organisch-Chemisches Institut, Westfälische Wilhelms-Universität Münster, Corrensstraße 40, 48149 Münster, Germany

Supporting Information

Table of Contents

General Information S2

A) Reaction of $ClB(C_6F_5)_2$ with excess allene S3

B) Reaction of $BrB(C_6F_5)_2$ with excess allene S7

C) Synthesis of compound **4a** S11

D) Synthesis of compound **4b** S16

E) Synthesis of compound **12** S20

Experiment 1: isolation of a mixture of compounds **11a** and **12** S20

Experiment 2: isolation and characterization of compound **12** S23

Experiment 3: compound **11b** S27

F) $XB(C_6F_5)_2$ (X = Cl, Br) catalyzed cyclotrimerization of substituted allenes **13** S31

G) Isomerization of cyclotrimer **1** S37

H) Isomerization of cyclotrimer **14c** S39

I) Isomerization of cyclotrimer **14e** S41

General Information. All reactions involving air- or moisture-sensitive compounds were carried out under an inert gas atmosphere (Argon) by using Schlenk-type glassware or in a glovebox. All solvents were dried and degassed before use, if necessary for the respective reaction. Chemicals: Unless otherwise noted all chemicals were used as purchased. The following instruments were used for physical characterization of the compounds: elemental analyses: Foss-Heraeus CHNO-Rapid; NMR: Varian UNITY plus NMR spectrometer (^{1}H, 600 MHz; ^{13}C, 151 MHz; ^{11}B, 192 MHz; ^{19}F, 564 MHz; ^{31}P, 243 MHz). NMR chemical shifts are given relative to $SiMe_4$ and referenced to the respective solvent signals (^{1}H and ^{13}C) or external standard [$\delta(BF_3{\cdot}OEt_2)$ = 0 for ^{11}B NMR, $\delta(CFCl_3{\cdot}OEt_2)$ = 0 for ^{19}F NMR]. NMR assignments were supported by additional 2D NMR experiments.

X-Ray diffraction: For compound **12** data sets were collected with a Nonius Kappa CCD diffractometer. Programs used: data collection, COLLECT (R. W. W. Hooft, Bruker AXS, **2008**, Delft, The Netherlands); data reduction Denzo-SMN (Z. Otwinowski, W. Minor, *Methods Enzymol.* **1997**, *276*, 307-326); absorption correction, Denzo (Z. Otwinowski, D. Borek, W. Majewski, W. Minor, *Acta Crystallogr*. **2003**, *A59*, 228-234); structure solution SHELXS-97 (G. M. Sheldrick, *Acta Crystallogr*. **1990**, *A46*, 467-473); structure refinement SHELXL-97 (G. M. Sheldrick, *Acta Crystallogr*. **2008**, *A64*, 112-122). Data sets for compounds **4b** and **15e** were collected with a D8 Venture CMOS diffractometer. For compound **4a** data sets were collected with a Bruker APEX II CCD diffractometer. Programs used: data collection: APEX3 V2016.1-0 (Bruker AXS Inc., **2016**); cell refinement: SAINT V8.37A (Bruker AXS Inc., **2015**); data reduction: SAINT V8.37A (Bruker AXS Inc., **2015**); absorption correction, SADABS V2014/7 (Bruker AXS Inc., **2014**); structure solution SHELXT-2015 (Sheldrick, **2015**); structure refinement SHELXL-2015 (Sheldrick, **2015**) and graphics, XP (Bruker AXS Inc., **2015**). *R*-values are given for observed reflections, and wR^2 values are given for all reflections. *Exceptions and special features*: For compounds **4a** and **4b** the CH_2-CCl=CH_2 and CH_2-CBr=CH_2 units were found disordered over two positions in the asymmetric unit. Several restraints (SADI, SAME, ISOR and SIMU) were used in order to improve refinement stability. Moreover, for compound **4a** a badly disordered half C_3H_4 (allene) molecule was found in the asymmetrical unit and could not be satisfactorily refined. The program SQUEEZE (Spek, A.L. (**2015**). Acta Cryst. C71, 9-18) was therefore used to remove mathematically the effect of the solvent. The quoted formula and derived parameters are not included the squeezed allene molecule. For compound **15e** two cyclohexyl and

one methyl groups were found disordered over two positions in the asymmetric unit. Several restraints (SADI, SAME, ISOR and SIMU) were used in order to improve refinement stability.CCDC deposition numbers are 1862533 to 1862535 and 1881527.

Materials. $ClB(C_6F_5)_2$ and $BrB(C_6F_5)_2$ were prepared according to procedures described in the literature [J. Li, C. G. Daniliuc, G. Kehr and G. Erker, *Chem. Commun.*, 2018, **54**, 6344; A. Ueno, J. Li, C. G. Daniliuc, G. Kehr and G. Erker, *Chem. Eur. J.*, 2018, **24**, 10044]. Allene was purchased from abcr GmbH in 96% purity, and used as received. Compound **14e** was prepared according to the procedures described in the literature [X. Tao, G. Kehr, C. G. Daniliuc and G. Erker, *Angew. Chem. Int. Ed.,* 2017, **56**, 1376].

A) Reaction of $ClB(C_6F_5)_2$ with excess allene

Scheme S1.

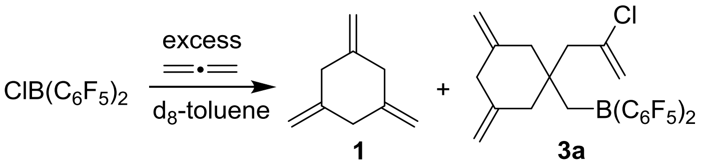

1 **3a**

$ClB(C_6F_5)_2$ (22.8 mg, 0.06 mmol) was dissolved in d_8-toluene (0.5 mL) in a Young NMR tube. After evacuating the tube, the solution was exposed to allene gas for several minutes at room temperature. Then the resulting reaction mixture was characterized by NMR experiments.

<u>*The reaction after 7 hours at r.t.*</u>: a mixture with allene (ca. 75 mol%, ^1H), compound **1** (ca. 8 mol%, ^1H), compound **3a** (ca. 7 mol%, ^1H), and unreacted $ClB(C_6F_5)_2$ (ca. 10 mol%, ^{19}F) as major components.

<u>*The reaction after 24 hours at r.t.*</u>: a mixture with allene (ca. 68 mol%, ^1H), compound **1** (ca. 15 mol%, ^1H), compound **3a** (ca. 14 mol%, ^1H), and unreacted $ClB(C_6F_5)_2$ (ca. 3 mol%, ^{19}F) as major components.

<u>*The reaction after 48 hours at r.t.*</u>: a mixture with allene (ca. 70 mol%, ^1H), compound **1** (ca. 15 mol%, ^1H), compound **3a** (ca. 14 mol%, ^1H) and unreacted $ClB(C_6F_5)_2$ (ca. < 1 mol%, ^{19}F) as major components

The NMR data of compound **1** in the reaction mixture are consistent with those reported in the literature [X. Tao, G. Kehr, C. G. Daniliuc and G. Erker, *Angew. Chem. Int. Ed.,* 2017, **56**, 1376]

NMR data of compound **1**:

^{1}H NMR (600 MHz, 299 K, d_8-toluene): δ ^{1}H: 4.53 (m, 2H, $=CH_2$), 2.70 (m, 2H, CH_2).

^{13}C{^{1}H} NMR (151 MHz, 299 K, d_8-toluene): δ ^{13}C: 145.9 (=C), 107.8 ($=CH_2$), 43.4 (CH_2).

Compound **3a** was characterized by NMR experiments from the reaction mixture after 7 hours at r.t.: NMR data of compound **3a**:

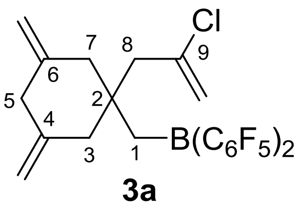

3a

^{1}H NMR (600 MHz, 299 K, d_8-toluene): δ ^{1}H: [5.08, 4.84](each m, each 1H, 9-CH_2=), [4.63, 4.48](each m, each 2H, 4,6-CH_2=), [2.58, 2.54](each d, $^{2}J_{HH}$ = 14.0 Hz, each 1H, 5-CH_2), 2.26 (s, 2H, 8-CH_2), [2.24, 2.00](each d, $^{2}J_{HH}$ = 14.0 Hz, each 2H, 3,7-CH_2), 2.18 (s, 2H, BCH_2).

^{13}C{^{1}H} NMR (151 MHz, 299 K, d_8-toluene): δ ^{13}C: 146.6 (dm, $^{1}J_{FC}$ ~ 240 Hz, C_6F_5), 144.1 (4,6-C=), 143.4 (dm, $^{1}J_{FC}$ ~ 250 Hz, C_6F_5), 140.0 (CCl=), 137.7 (dm, $^{1}J_{FC}$ ~ 250 Hz, C_6F_5), 117.1 (9-CH_2=), 115.7 (br, i-C_6F_5), 111.2 (4,6-CH_2=), 48.5 (8-CH_2), 46.6 (3,7-CH_2), 43.38 (5-CH_2), 42.9 (2-C), 42.2 (br, BCH_2).

^{19}F NMR (564 MHz, 299 K, d_8-toluene): δ ^{19}F: [–129.3 (m, 2F, *o*), –147.9 (tt, $^{3}J_{FF}$ = 21.0 Hz, J_{FF} = 4.4 Hz, 1F, *p*), –161.1 (m, 2F, *m*)](C_6F_5)[$\Delta\delta^{19}F_{m,p}$ = 13.2].

^{11}B{^{1}H} NMR (192 MHz, 299 K, d_8-toluene): δ ^{11}B: 71.5 ($\nu_{1/2}$ ~ 1000 Hz).

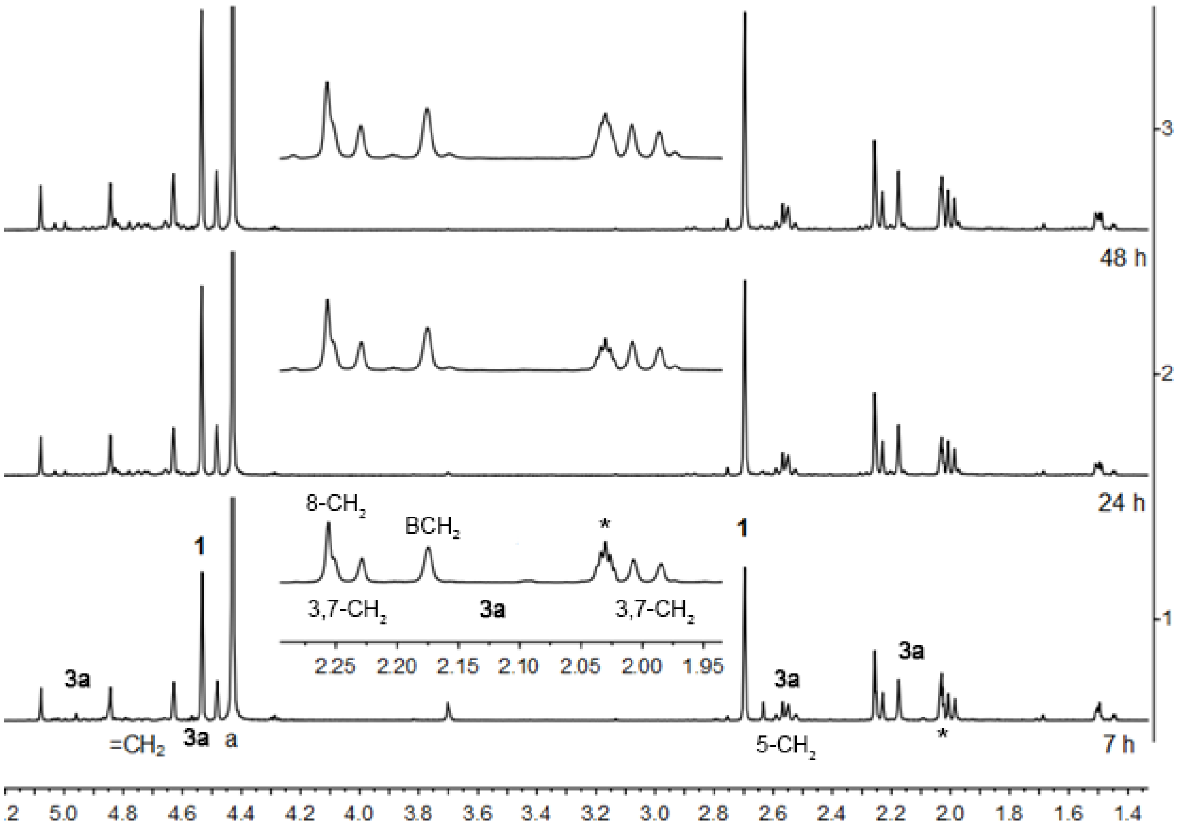

Figure S1. ^1H NMR (600 MHz, 299 K, d_8-toluene*) spectra of the mixture of the reaction of $ClB(C_6F_5)_2$ with excess allene after different reaction times at r.t.: 7 hours (spectrum 1); 24 hours (spectrum 2); 48 hours (spectrum 3). [a: allene]

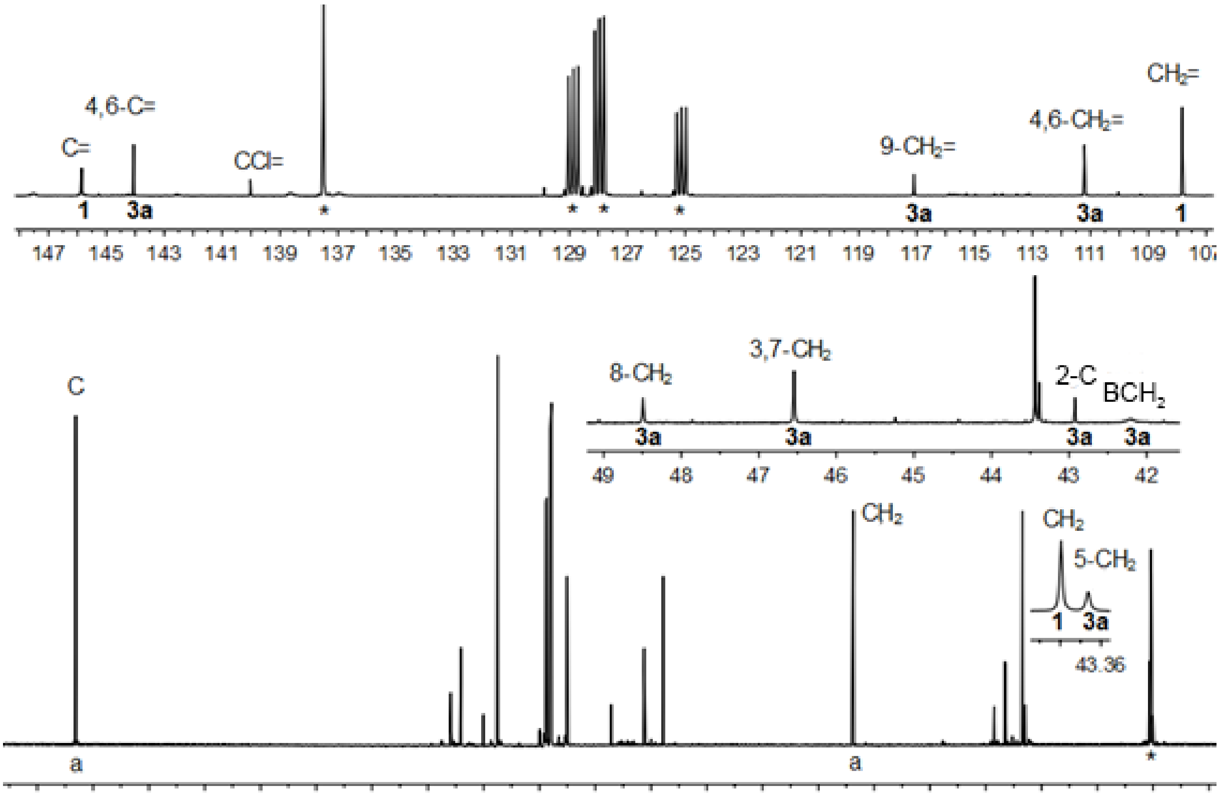

Figure S2. $^{13}C\{^1H\}$ NMR (151 MHz, 299 K, d_8-toluene*) spectrum of the mixture of the reaction of $ClB(C_6F_5)_2$ with excess allene after 7 hours at r.t. [a: allene]

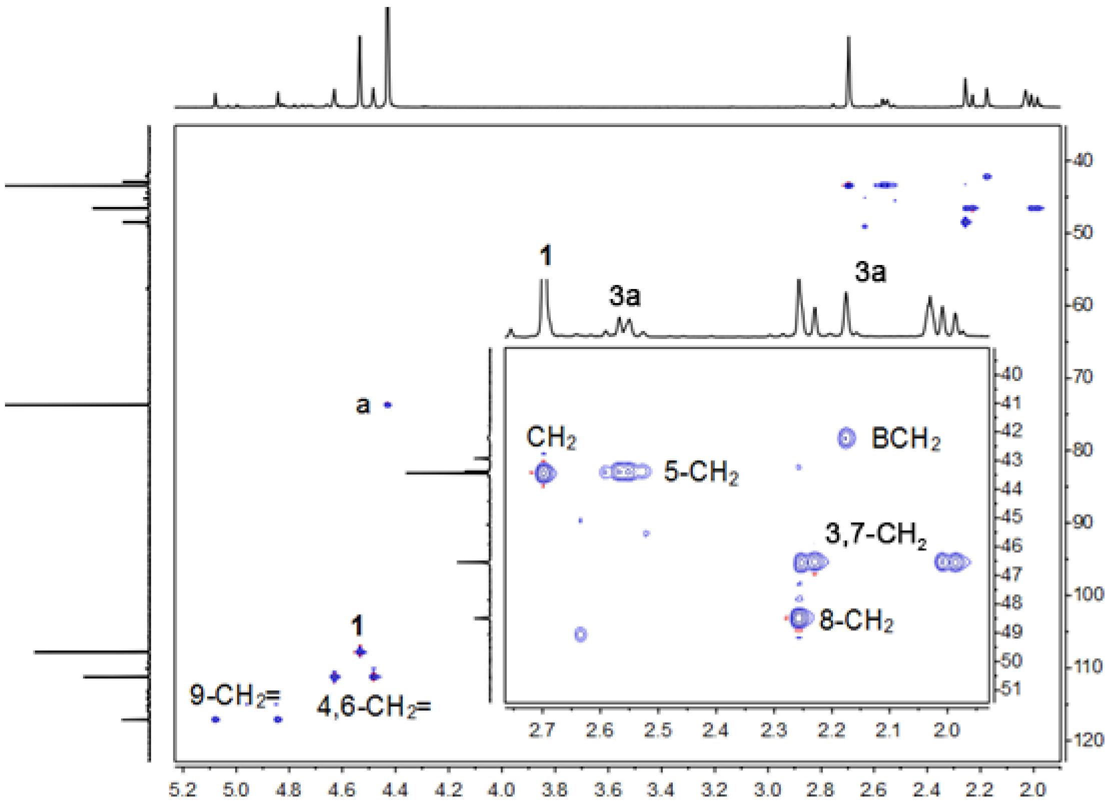

Figure S3. $^{1}H/^{13}C$ GHSQC (600 MHz/151 MHz, 299 K, d_8-toluene*) spectrum of the mixture of the reaction of $ClB(C_6F_5)_2$ with excess allene after 7 hours at r.t. [a: allene]

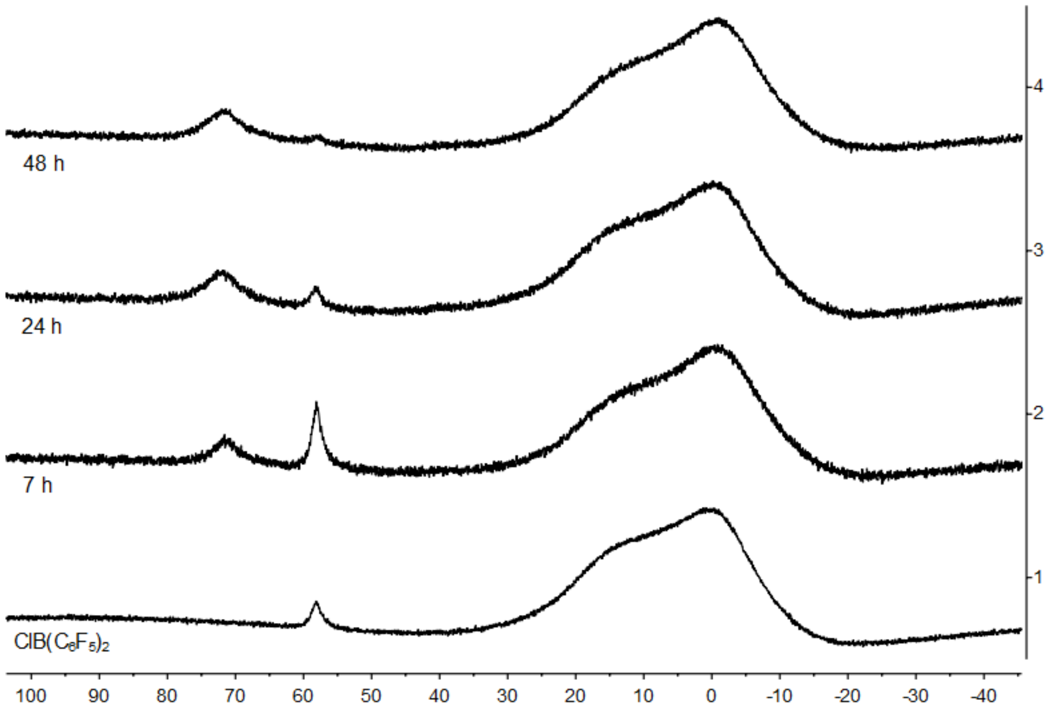

Figure S4. $^{11}B\{^{1}H\}$ NMR (192 MHz, 299 K, d_8-toluene) spectra of (1) $ClB(C_6F_5)_2$ and the mixture of the reaction of $ClB(C_6F_5)_2$ with excess allene after different reaction times at r.t.: (2) 7 hours, (3) 24 hours, and (4) 48 hours.

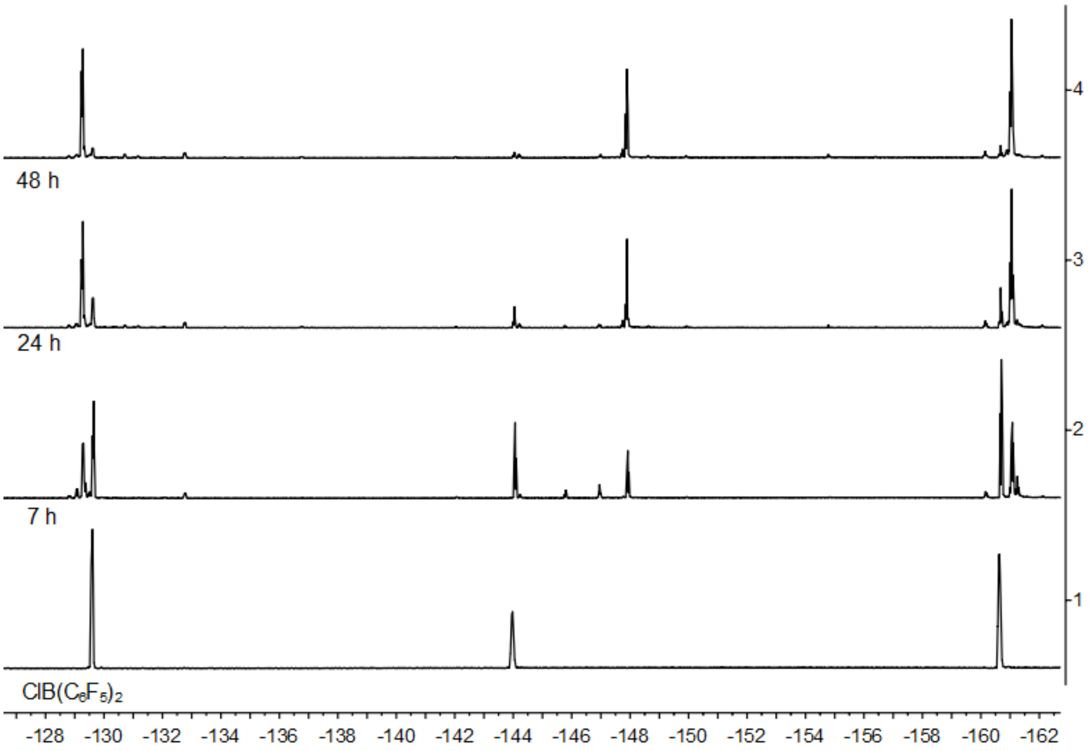

Figure S5. ^{19}F NMR (564 MHz, 299 K, d_8-toluene) spectra of (1) $ClB(C_6F_5)_2$ and the mixture of the reaction of $ClB(C_6F_5)_2$ with excess allene after different reaction times at r.t.: (2) 7 hours, (3) 24 hours, and (4) 48 hours.

B) Reaction of $BrB(C_6F_5)_2$ with excess allene

Scheme S2.

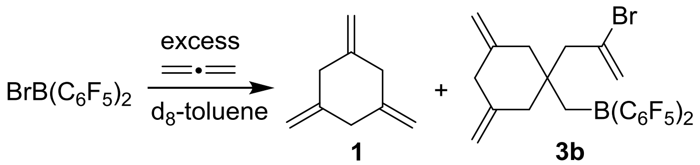

$BrB(C_6F_5)_2$ (25.4 mg, 0.06 mmol) was dissolved in d_8-toluene (0.5 mL) in a Young NMR tube. After evacuating the tube, the solution was exposed to allene gas for several minutes at room temperature. Then the resulting reaction mixture was characterized by NMR experiments after 4 hours at room temperature: a mixture with allene (ca. 91 mol%, ^{1}H), compound **1** (ca. 3 mol%, ^{1}H), and compound **3b** (ca. 6 mol%, ^{1}H) as major components.

The NMR data of compound **1** in the reaction mixture are consistent with those reported in the literature [X. Tao, G. Kehr, C. G. Daniliuc and G. Erker, *Angew. Chem. Int. Ed.,* 2017, **56**, 1376.]

NMR data of compound **1**:

^{1}H NMR (600 MHz, 299 K, d_8-toluene): δ ^{1}H: 4.53 (m, 2H, $=CH_2$), 2.70 (m, 2H, CH_2).

^{13}C{^{1}H} NMR (151 MHz, 299 K, d_8-toluene): δ ^{13}C: 145.9 (=C), 107.8 ($=CH_2$), 43.44 (CH_2).

Compound **3b** was characterized by NMR spectroscopy from the reaction mixture after 7 hours at r.t., NMR data of compound **3b**:

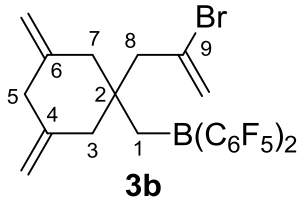

3b

^{1}H NMR (600 MHz, 299 K, d_8-toluene): δ ^{1}H: [5.34, 5.27](each m, each 1H, 9-CH_2=), [4.62, 4.48](each m, each 2H, 4,6-CH_2=), [2.57, 2.53](each d, $^2J_{HH}$ = 14.0 Hz, each 1H, 5-CH_2), 2.38 (s, 2H, 8-CH_2), [2.27, 1.97](each d, $^2J_{HH}$ = 14.0 Hz, each 2H, 3,7-CH_2), 2.21 (s, 2H, BCH_2).

^{13}C{^{1}H} NMR (151 MHz, 299 K, d_8-toluene): δ ^{13}C: 146.6 (dm, $^1J_{FC}$ ~ 240 Hz, C_6F_5), 144.1 (4,6-C=), 143.4 (dm, $^1J_{FC}$ ~ 260 Hz, C_6F_5), 137.7 (dm, $^1J_{FC}$ ~ 250 Hz, C_6F_5), 130.3 (CBr=), 121.7 (9-CH_2=), 115.7 (br, i-C_6F_5), 111.2 (4,6-CH_2=), 50.6 (8-CH_2), 46.5 (3,7-CH_2), [43.36, 43.35](2-C, 5-CH_2), 42.2 (br, BCH_2).

^{19}F NMR (564 MHz, 299 K, d_8-toluene): δ ^{19}F: [–129.3 (m, 2F, *o*), –147.9 (t, $^3J_{FF}$ = 21.0 Hz, 1F, *p*), –161.1 (m, 2F, *m*)](C_6F_5)[$\Delta\delta^{19}F_{m,p}$ = 13.1].

^{11}B{^{1}H} NMR (192 MHz, 299 K, d_8-toluene): δ ^{11}B: 72.3 ($\nu_{1/2}$ ~ 1200 Hz).

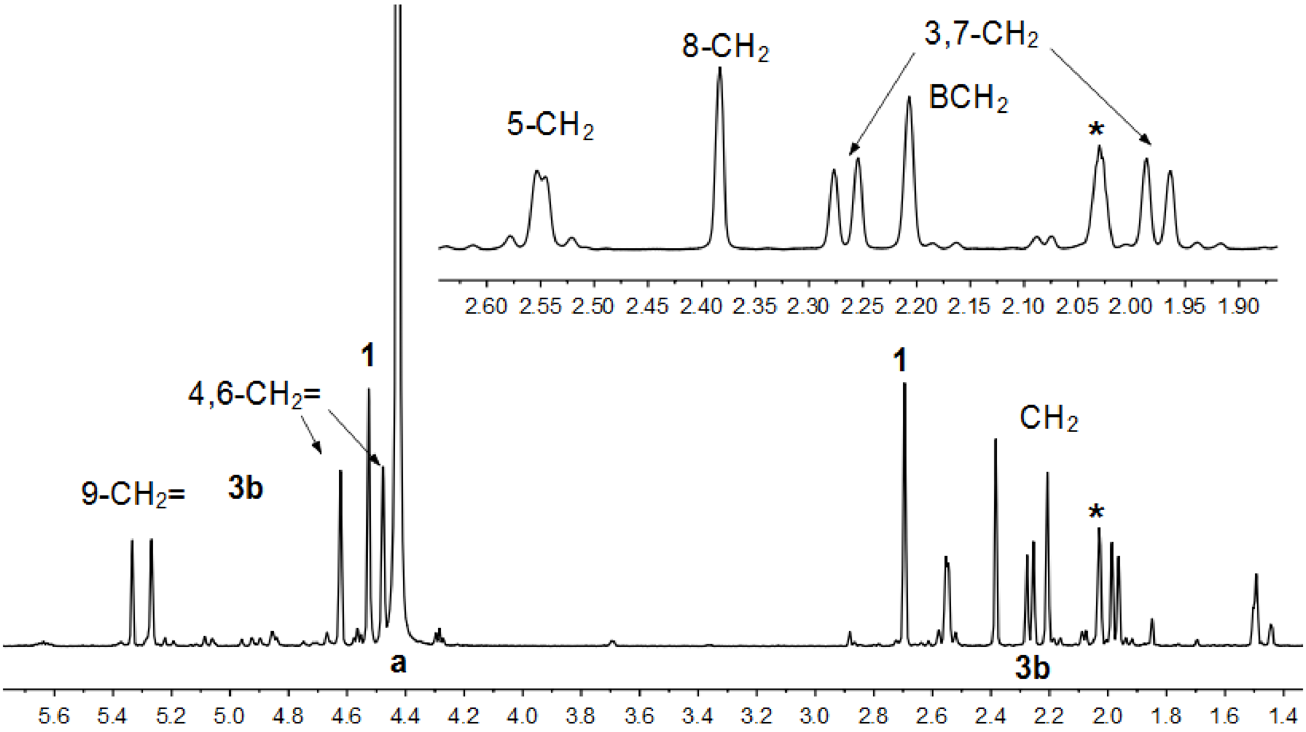

Figure S6. ^{1}H NMR (600 MHz, 299 K, d_8-toluene*) spectrum of the mixture of the reaction of $BrB(C_6F_5)_2$ with excess allene after 4 hours at r.t. [a: allene]

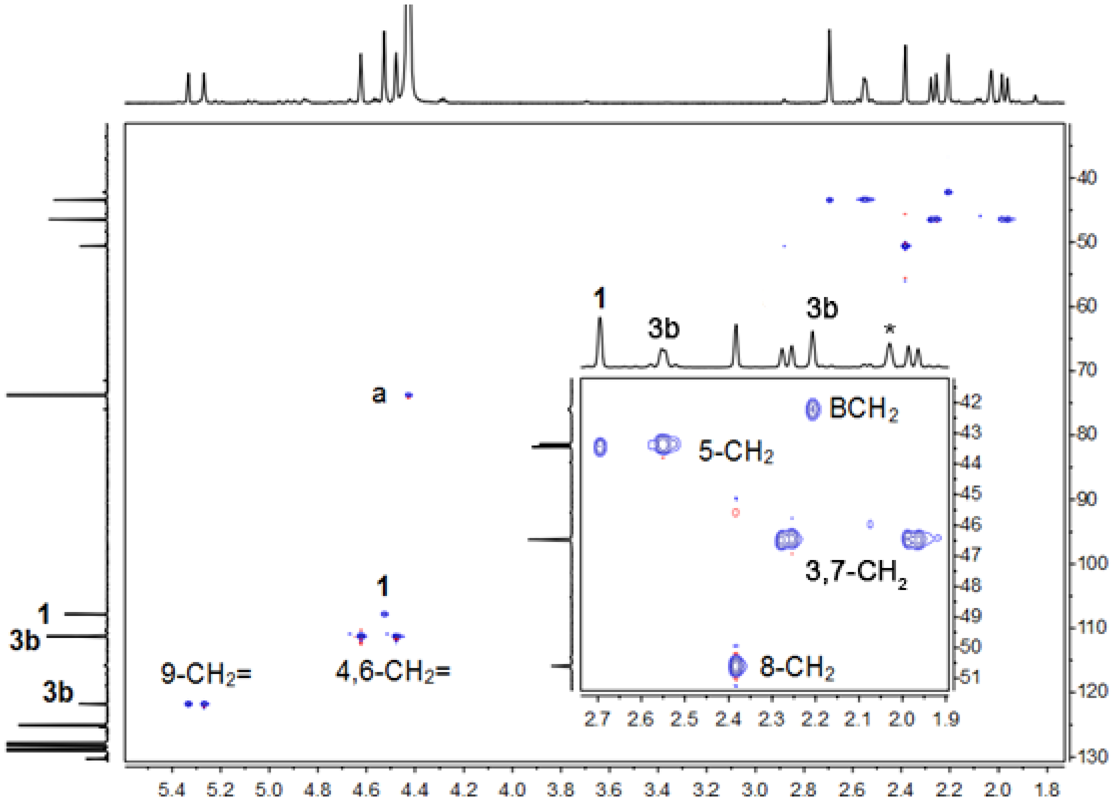

Figure S7. $^{1}H/^{13}C$ GHSQC (600 MHz/151 MHz, 299 K, d_8-toluene*) spectrum of the mixture of the reaction of $BrB(C_6F_5)_2$ with excess allene after 4 hours at r.t. [a: allene]

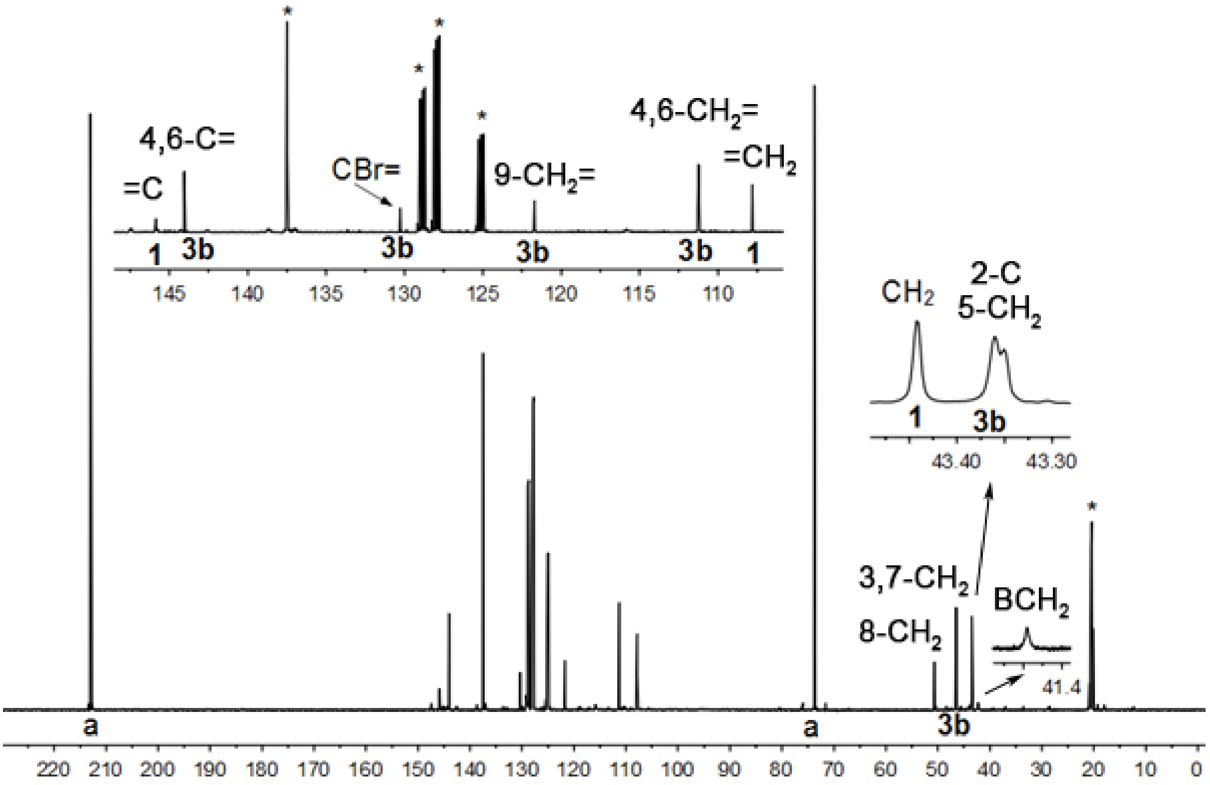

Figure S8. $^{13}C\{^1H\}$ NMR (151 MHz, 299 K, d_8-toluene*) spectrum of the mixture of the reaction of $BrB(C_6F_5)_2$ with excess allene after 4 hours at r.t. [a: allene]

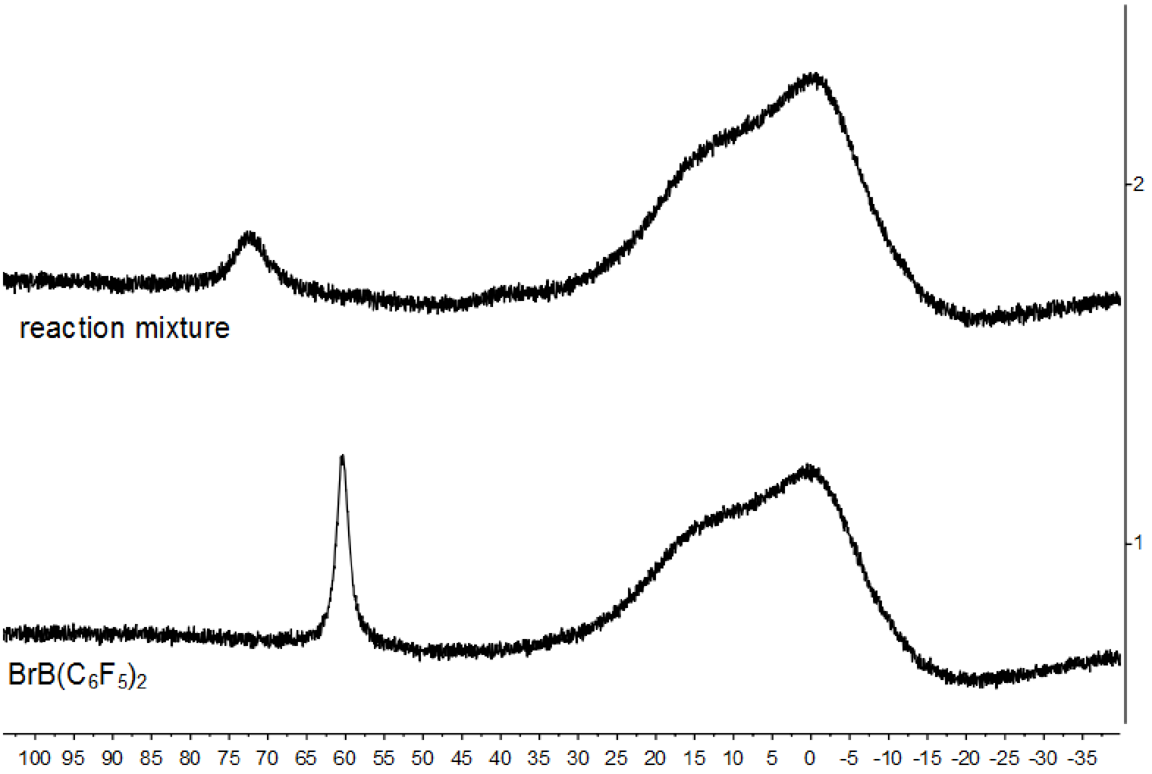

Figure S9. $^{11}B\{^1H\}$ NMR (192 MHz, 299 K, d_8-toluene) spectra of (1) $BrB(C_6F_5)_2$ and (2) the mixture of the reaction of $BrB(C_6F_5)_2$ with excess allene after 4 hours at r.t.

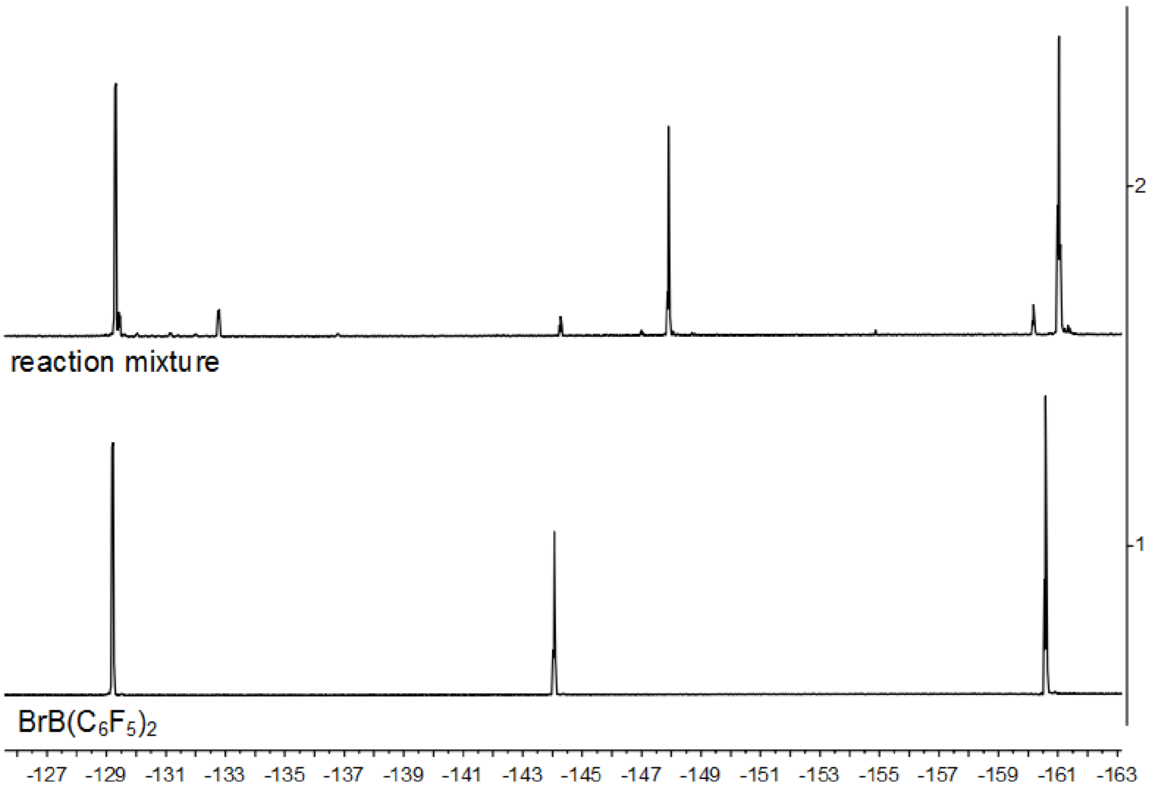

Figure S10. ^{19}F NMR (564 MHz, 299 K, d_8-toluene) spectra of (1) $BrB(C_6F_5)_2$ and (2) the mixture of the reaction of $ClB(C_6F_5)_2$ with excess allene after 4 hours at r.t.

C) Synthesis of compound **4a**

Scheme S3.

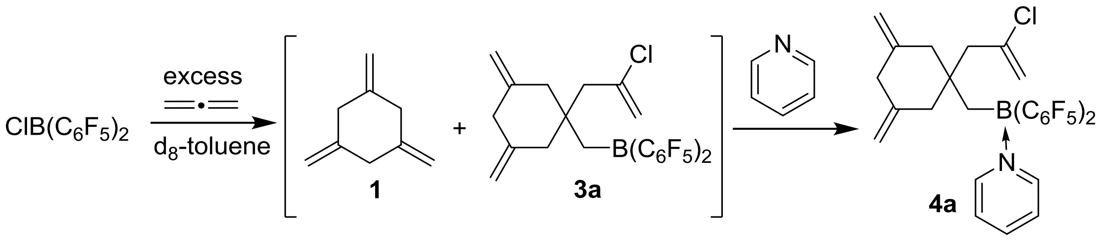

$ClB(C_6F_5)_2$ (120 mg, 0.320 mmol) was dissolved in d_8-toluene (2.0 mL) in a Schlenk tube. After evacuating the Schlenk tube, the solution was exposed to allene gas for several minutes at room temperature. Then the resulting reaction mixture was stirred for 48 hours at room temperature. Subsequently, pyridine (30.0 mg, 0.380 mmol) was added. After stirring the reaction mixture for 10 min. at room temperature, all volatiles were removed in vacuo and the residue was washed with pentane (1 mL × 3). Drying of the remaining solid in vacuo gave compound **4a** (105 mg, 0.170 mmol, 53%) as a white powder.

Anal. Calc. for $C_{29}H_{21}BClF_{10}N$: C, 56.20; H, 3.42; N, 2.26. Found: C, 56.77; H, 3.62; N, 2.24.

NMR data of compound **4a**:

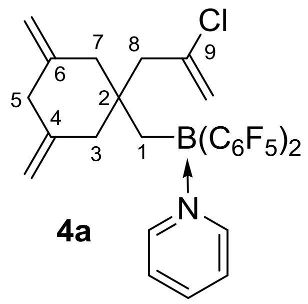

4a

^{1}**H NMR** (600 MHz, 299 K, CD_2Cl_2): δ ^{1}H: 8.77 (m, 2H, o-Py), 8.07 (m, 1H, p-Py), 7.64 (m, 2H, m-Py), [5.32, 5.12](each m, each 1H, 9-CH_2=), [4.67, 4.47](each m, each 2H, 4,6-CH_2=), [2.73, 2.57](each dm, $^{2}J_{HH}$ = 14.0 Hz, each 1H, 5-CH_2), 2.39 (s, 2H, 8-CH_2), [2.21, 1.68](each d, $^{2}J_{HH}$ = 13.5 Hz, each 2H, 3,7-CH_2), 1.74 (s, 2H, BCH_2).

^{13}**C{**^{1}**H} NMR** (151 MHz, 299 K, CD_2Cl_2): δ ^{13}C: 148.6 (dm, $^{1}J_{FC}$ ~ 240 Hz, C_6F_5), 146.2 (4,6-C=), 145.5 (o-Py), 141.9 (p-Py), 141.1 (CCl=), 140.0 (dm, $^{1}J_{FC}$ ~ 250 Hz, C_6F_5), 137.7 (dm, $^{1}J_{FC}$ ~ 250 Hz, C_6F_5), 126.7 (m-Py), 121.5 (br, i-C_6F_5), 116.6 (9-CH_2=), 109.6 (4,6-CH_2=), 48.7 (8-CH_2), 44.4 (3,7-CH_2), 43.9 (5-CH_2), 39.9 (2-C), 31.2 (br, BCH_2).

^{19}**F NMR** (564 MHz, 299 K, CD_2Cl_2): δ ^{19}F: [–129.4 (m, 2F, *o*), –158.4 (t, $^{3}J_{FF}$ = 21.0 Hz, 1F, *p*), –164.0 (m, 2F, *m*)](C_6F_5)[$\Delta\delta^{19}F_{m,p}$ = 5.6].

^{11}**B{**^{1}**H} NMR** (192 MHz, 299 K, CD_2Cl_2): δ ^{11}B: –1.4 ($\nu_{1/2}$ ~ 250 Hz).

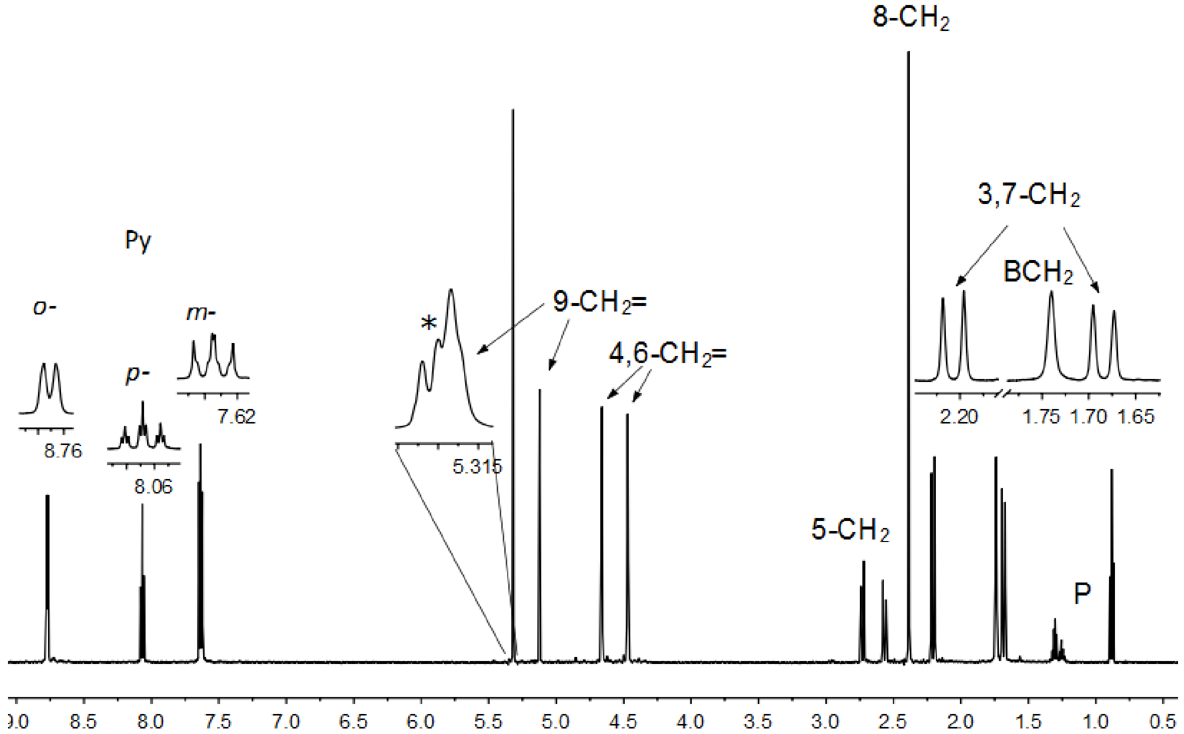

Figure S11. ^{1}H NMR (600 MHz, 299 K, CD_2Cl_2*) spectrum of compound **4a**. [P: pentane; Py: pyridine]

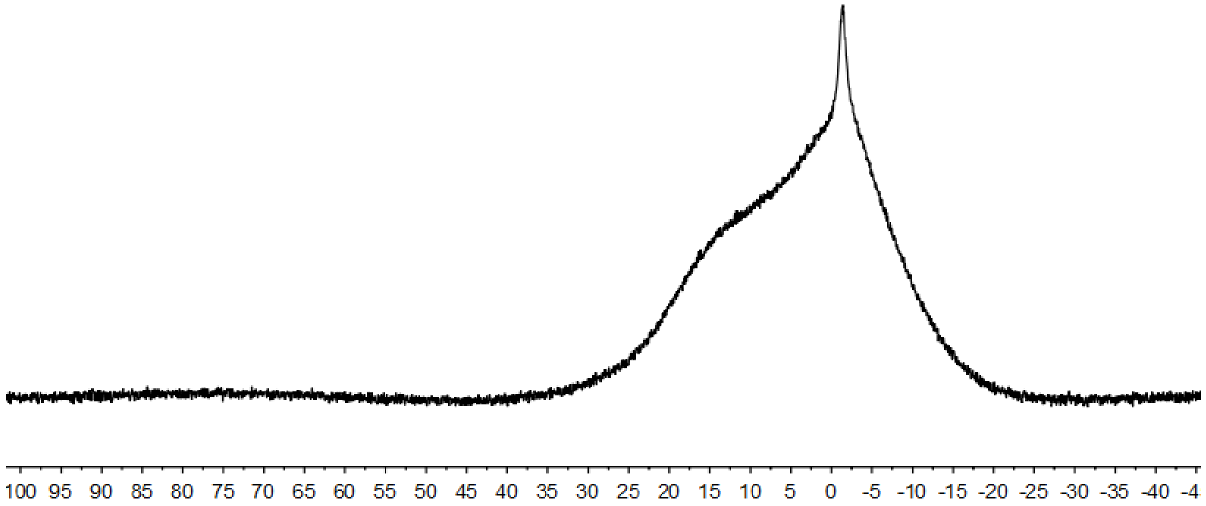

Figure S12. $^{11}B\{^1H\}$ NMR (192 MHz, 299 K, CD_2Cl_2) spectrum of compound **4a**.

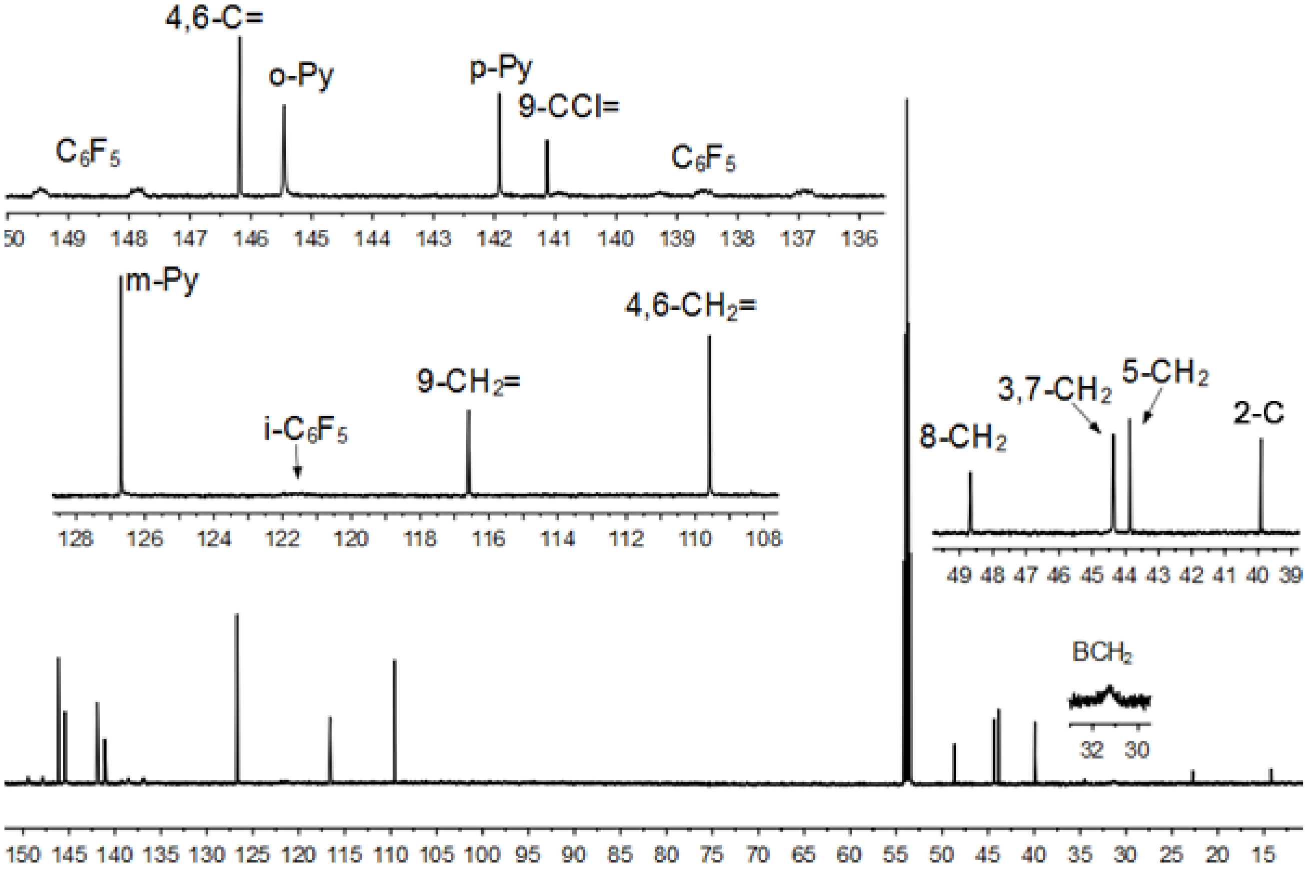

Figure S13. $^{13}C\{^1H\}$ NMR (151 MHz, 299 K, CD_2Cl_2) spectrum of compound **4a**.[Py: pyridine]

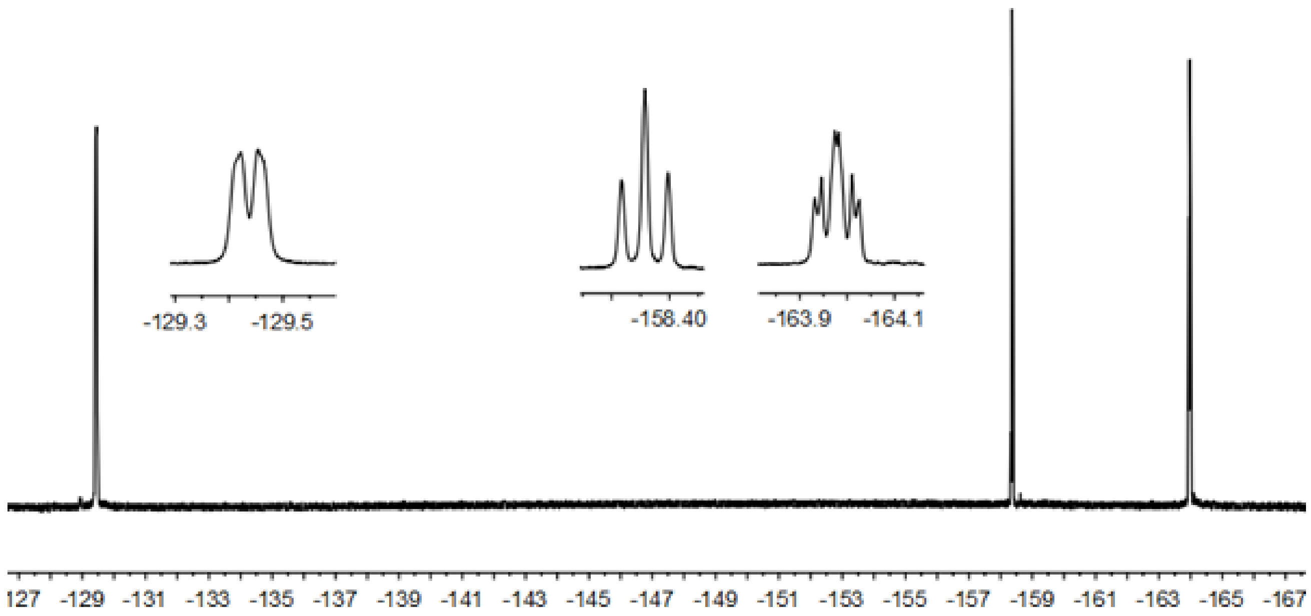

Figure S14. ^{19}F NMR (564 MHz, 299 K, CD_2Cl_2) spectrum of compound **4a**.

Single crystals suitable for the X-ray crystal structure analysis were obtained from diffusion of pentane vapor to a solution of the white powder in CH_2Cl_2 at room temperature.

X-ray crystal structure analysis of compound 4a (erk9164): A colorless plate-like specimen of $C_{29}H_{21}BClF_{10}N$, approximate dimensions 0.020 mm x 0.060 mm x 0.100 mm, was used for the X-ray crystallographic analysis. The X-ray intensity data were measured. A total of 1882 frames were collected. The total exposure time was 34.10 hours. The frames were integrated with the Bruker SAINT software package using a wide-frame algorithm. The integration of the data using a triclinic unit cell yielded a total of 20385 reflections to a maximum θ angle of 66.76° (0.84 Å resolution), of which 4780 were independent (average redundancy 4.265, completeness = 98.3%, R_{int} = 9.58%, R_{sig} = 10.26%) and 2842 (59.46%) were greater than 2σ(F^2). The final cell constants of a = 10.2284(12) Å, b = 11.0264(13) Å, c = 12.5397(13) Å, α = 88.589(8)°, β = 80.364(7)°, γ = 79.511(8)°, volume = 1371.0(3) $Å^3$, are based upon the refinement of the XYZ-centroids of 2983 reflections above 20 σ(I) with 8.155° < 2θ < 129.2°. Data were corrected for absorption effects using the multi-scan method (SADABS). The ratio of minimum to maximum apparent transmission was 0.823. The calculated minimum and maximum transmission coefficients (based on crystal size) are 0.8210 and 0.9600. The

structure was solved and refined using the Bruker SHELXTL Software Package, using the space group $P\bar{1}$, with Z = 2 for the formula unit, $C_{29}H_{21}BClF_{10}N$. The final anisotropic full-matrix least-squares refinement on F^2 with 408 variables converged at R1 = 5.70%, for the observed data and wR2 = 16.70% for all data. The goodness-of-fit was 0.972. The largest peak in the final difference electron density synthesis was 0.267 $e^-/Å^3$ and the largest hole was -0.255 $e^-/Å^3$ with an RMS deviation of 0.059 $e^-/Å^3$. On the basis of the final model, the calculated density was 1.501 g/cm^3 and F(000), 628 e^-. CCDC deposition number 1862533.

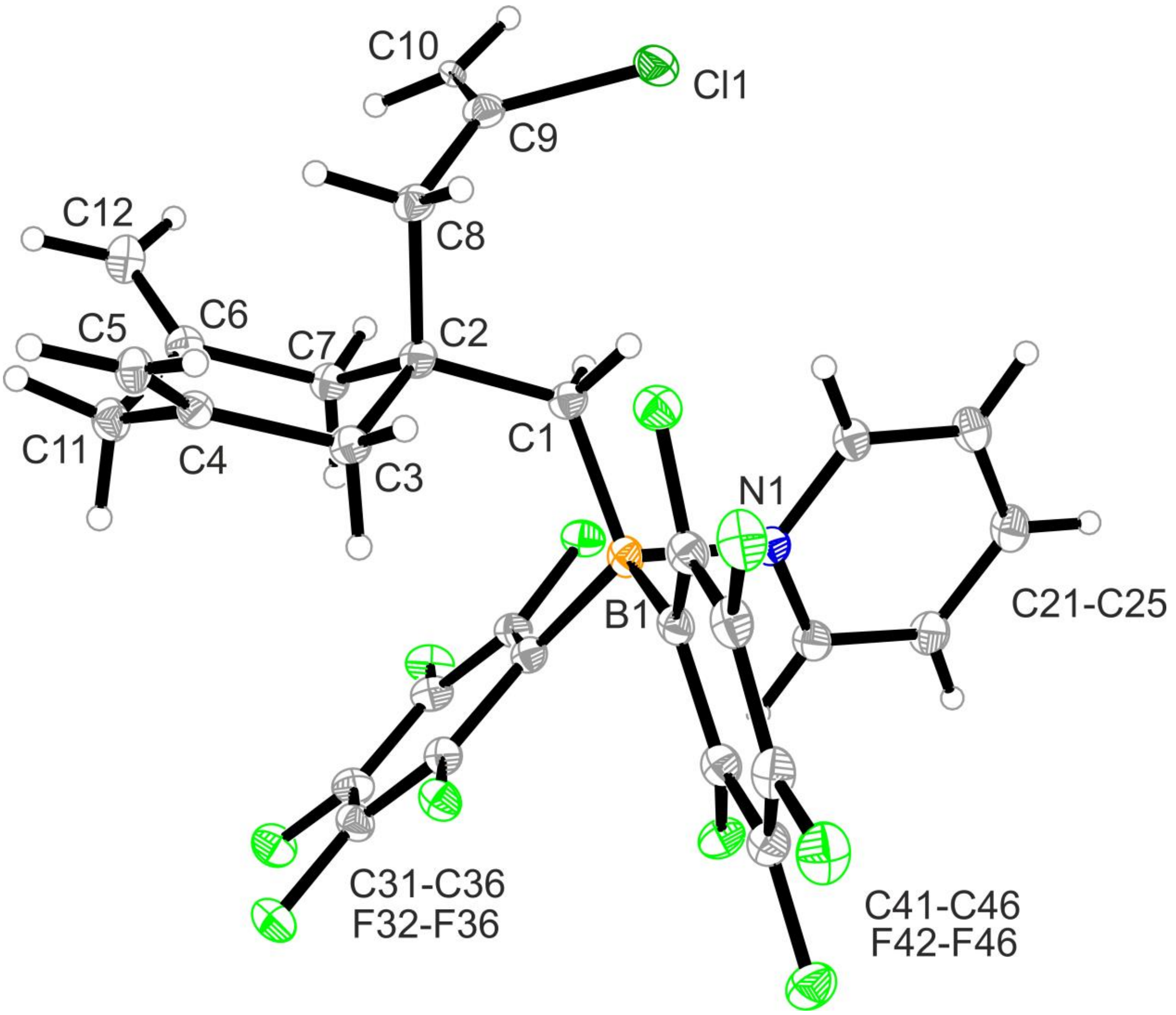

Figure S15. Crystal structure of compound **4a** (thermal ellipsoids: 15% probability)

D) Synthesis of compound **4b**

Scheme S4.

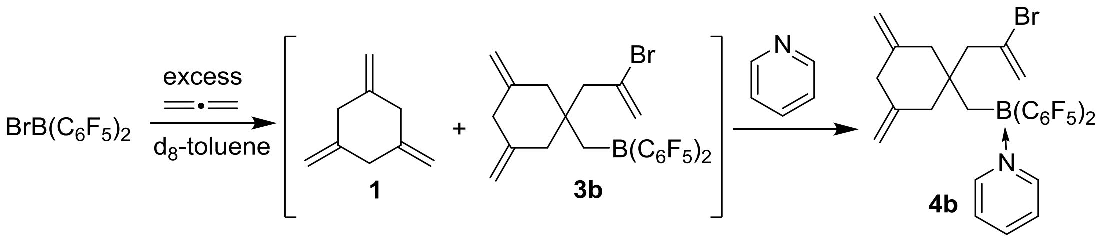

$BrB(C_6F_5)_2$ (127 mg, 0.300 mmol) was dissolved in d_8-toluene (2.0 mL) in a Schlenk tube. After evacuating the tube, the solution was exposed to allene gas for several minutes at room temperature. Then the resulting reaction mixture was stirred for 48 hours at room temperature. Subsequently, pyridine (30.0 mg, 0.380 mmol) was added to the resulting reaction mixture. After stirring for 10 min at room temperature, all volatiles were removed in vacuo and the residue was washed with pentane (1 mL × 3). Drying of the remaining solid in vacuo gave compound **4b** (144 mg, 0.220 mmol, 72%) as a white powder.

Anal. Calc. for $C_{29}H_{21}BBrF_{10}N$: C, 52.44; H, 3.19; N, 2.11. Found: C, 52.47; H, 3.23; N, 2.10.

NMR data of compound **4b**:

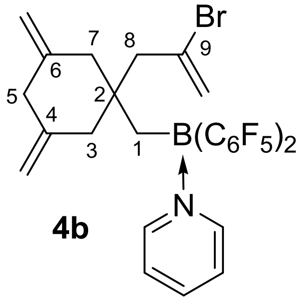

^{1}H NMR (600 MHz, 299 K, CD_2Cl_2): δ ^{1}H: 8.79 (m, 2H, o-Py), 8.07 (m, 1H, p-Py), 7.64 (m, 2H, m-Py), [5.60, 5.57](each m, each 1H, 9-CH_2=), [4.66, 4.47](each m, each 2H, 4,6-CH_2=), [2.73, 2.57](each dm, $^2J_{HH}$ = 14.0 Hz, each 1H, 5-CH_2), 2.52 (s, 2H, 8-CH_2), [2.23, 1.68](each d, $^2J_{HH}$ = 13.5 Hz, each 2H, 3,7-CH_2), 1.77 (s, 2H, BCH_2).

^{13}C{^{1}H} NMR (151 MHz, 299 K, CD_2Cl_2): δ ^{13}C: 148.6 (dm, $^1J_{FC}$ ~ 240 Hz, C_6F_5), 146.1 (4,6-C=), 145.5 (o-Py), 141.9 (p-Py), 140.0 (dm, $^1J_{FC}$ ~ 250 Hz, C_6F_5), 137.6 (dm, $^1J_{FC}$ ~ 250 Hz, C_6F_5), 131.6 (CBr=), 126.7 (m-Py), 121.5 (br, i-C_6F_5), 121.3 (9-CH_2=), 109.6 (4,6-CH_2=), 50.4 (8-CH_2), 44.3 (3,7-CH_2), 43.8 (5-CH_2), 40.4 (2-C), 31.5 (br, BCH_2).

^{19}F NMR (564 MHz, 299 K, CD_2Cl_2): δ ^{19}F: [–129.4 (m, 2F, *o*), –158.4 (t, $^3J_{FF}$ = 21.0 Hz, 1F, *p*), –164.0 (m, 2F, *m*)](C_6F_5)[$\Delta\delta^{19}F_{m,p}$ = 5.6].

^{11}B{^{1}H} NMR (192 MHz, 299 K, CD_2Cl_2): δ ^{11}B: –1.4 ($\nu_{1/2}$ ~ 220 Hz).

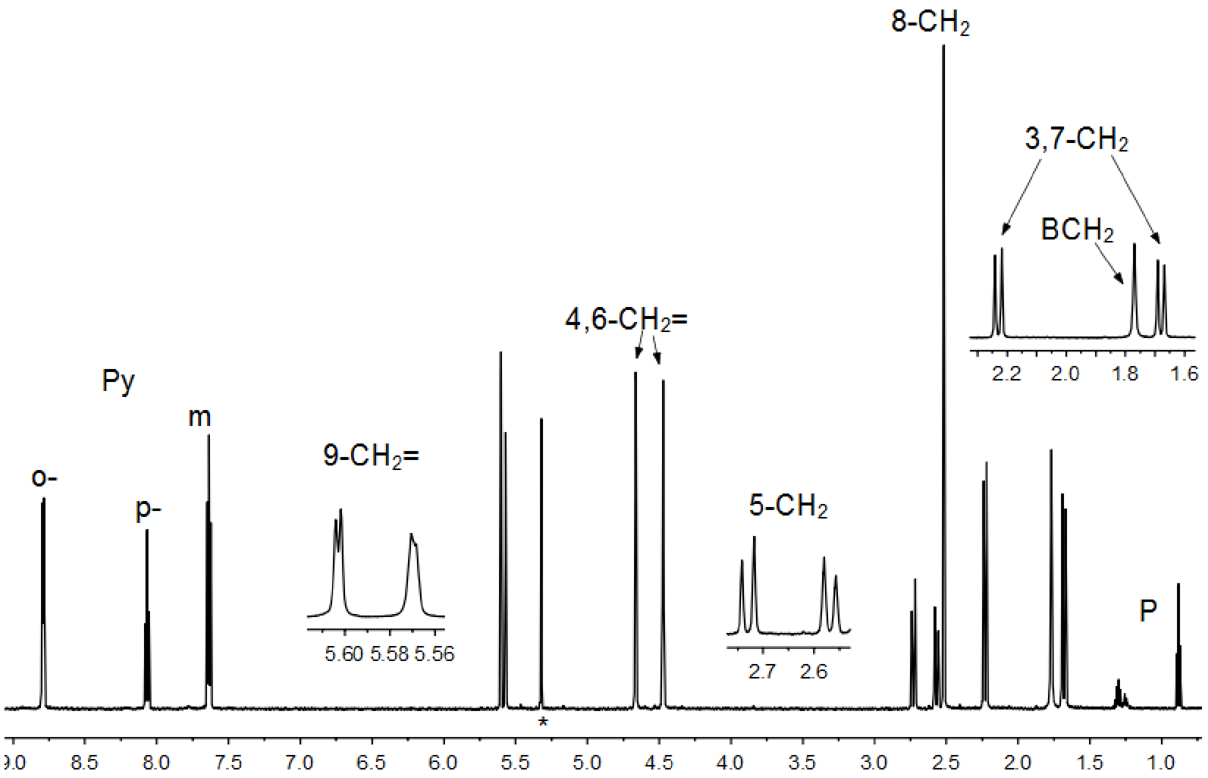

Figure S16. ^{1}H NMR (600 MHz, 299 K, CD_2Cl_2*) spectrum of compound **4b**. [P: pentane; Py: pyridine]

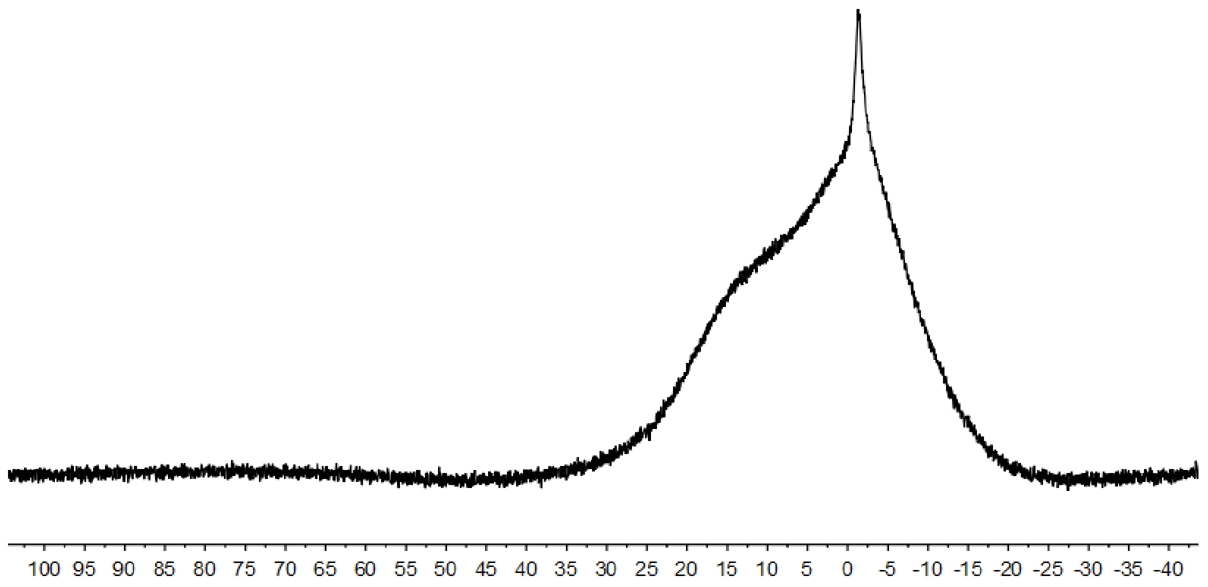

Figure S17. ^{11}B{^{1}H} NMR (192 MHz, 299 K, CD_2Cl_2) spectrum of compound **4b**.

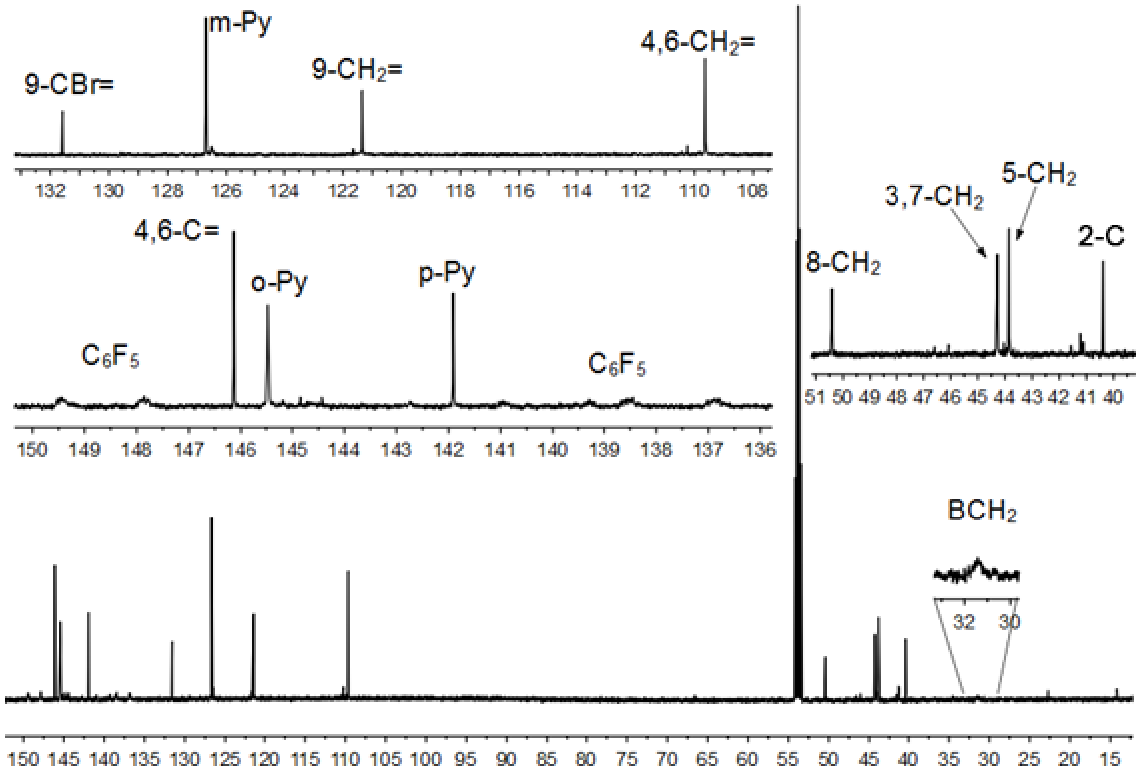

Figure S18. $^{13}C\{^{1}H\}$ NMR (151 MHz, 299 K, CD_2Cl_2) spectrum of compound **4b**.[Py: pyridine]

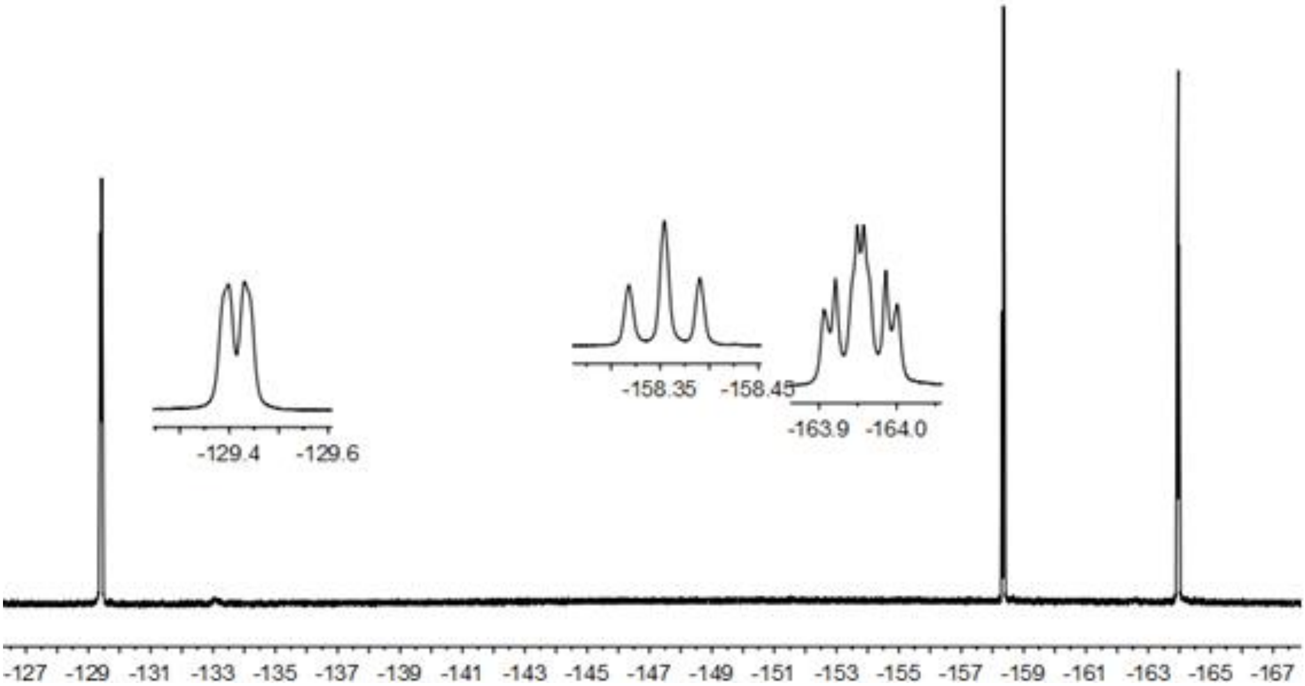

Figure S19. ^{19}F NMR (564 MHz, 299 K, CD_2Cl_2) spectrum of compound **4b**.

Single crystals suitable for the X-ray crystal structure analysis were obtained from diffusion of pentane vapor to a solution of the white powder in CH_2Cl_2 at room temperature.

X-ray crystal structure analysis of compound 4b (erk9170): A colorless prism-like specimen of $C_{29}H_{21}BBrF_{10}N$, approximate dimensions 0.158 mm x 0.170 mm x 0.303 mm, was used for the X-ray crystallographic analysis. The X-ray intensity data were measured. A total of 1382 frames were collected. The total exposure time was 16.14 hours. The frames were integrated with the Bruker SAINT software package using a wide-frame algorithm. The integration of the data using a triclinic unit cell yielded a total of 27371 reflections to a maximum θ angle of 70.23° (0.82 Å resolution), of which 5152 were independent (average redundancy 5.313, completeness = 98.8%, R_{int} = 3.26%, R_{sig} = 2.24%) and 4888 (94.88%) were greater than $2\sigma(F^2)$. The final cell constants of a = 10.2011(4) Å, b = 11.0143(4) Å, c = 12.5252(5) Å, α = 88.7430(10)°, β = 80.8650(10)°, γ = 79.6490(10)°, volume = 1366.81(9) $Å^3$, are based upon the refinement of the XYZ-centroids of 9975 reflections above 20 σ(I) with 7.148° < 2θ < 140.2°. Data were corrected for absorption effects using the multi-scan method (SADABS). The ratio of minimum to maximum apparent transmission was 0.845. The calculated minimum and maximum transmission coefficients (based on crystal size) are 0.4770 and 0.6600. The structure was solved and refined using the Bruker SHELXTL Software Package, using the space group $P\bar{1}$, with Z = 2 for the formula unit, $C_{29}H_{21}BBrF_{10}N$. The final anisotropic full-matrix least-squares refinement on F^2 with 407 variables converged at R1 = 2.95%, for the observed data and wR2 = 7.34% for all data. The goodness-of-fit was 1.039. The largest peak in the final difference electron density synthesis was 0.288 $e^-/Å^3$ and the largest hole was -0.416 $e^-/Å^3$ with an RMS deviation of 0.050 $e^-/Å^3$. On the basis of the final model, the calculated density was 1.614 g/cm^3 and F(000), 664 e^-. CCDC deposition number 1862534.

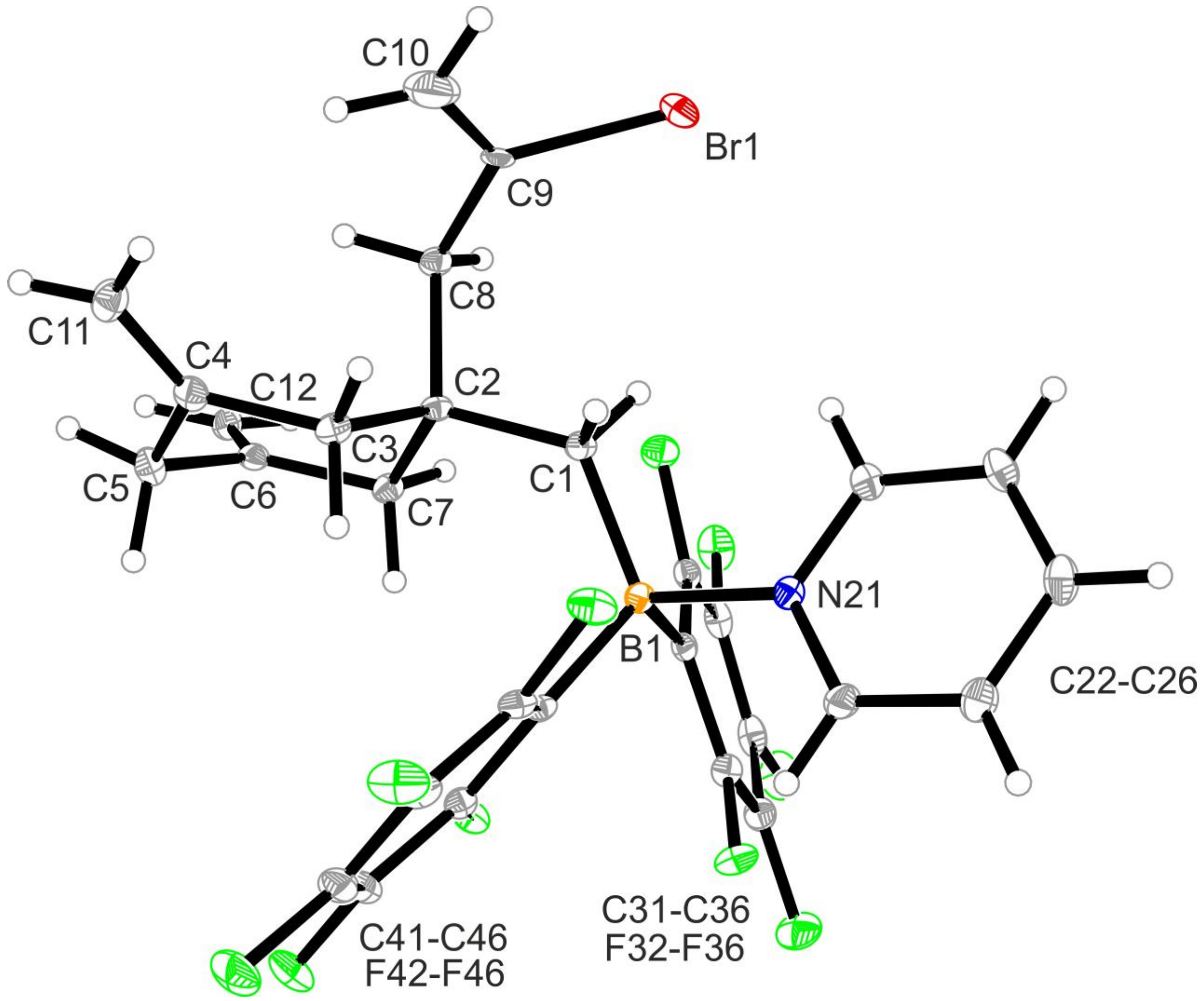

Figure S20. Crystal structure of compound **4b** (thermal ellipsoids: 30% probability)

E) Synthesis of compound **12**

Experiment 1: isolation of a mixture of compounds **11a** and **12**

Scheme S5.

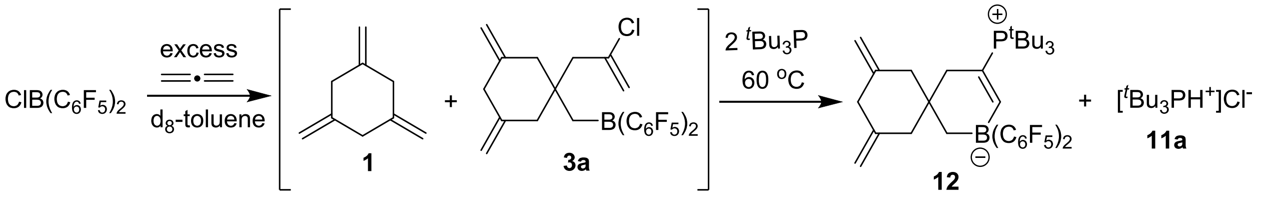

$ClB(C_6F_5)_2$ (165 mg, 0.434 mmol) was dissolved in d_8-toluene (2.0 mL) in a Schlenk tube. After evacuating the Schlenk tube, the solution was exposed to allene gas for several minutes at room temperature. Then the resulting reaction mixture was stirred for 48 hours at room temperature.

Subsequently, $^{t}Bu_3P$ (175 mg, 0.868 mmol) was added. After stirring the reaction mixture for 24 hours at 60 °C, all volatiles were removed in vacuo and pentane (1 mL) was added to the residual oil. Then CH_2Cl_2 was added dropwise to the stirred mixture until it became a suspension. The liquid was removed by filtration. Drying of the remaining solid in vacuo gave a white powder (165 mg, ca. 54%).

A solution of the obtained white powder in CD_2Cl_2 showed a mixture of two main components: compounds **12** (ca. 79 mol%, ^{1}H) and **11a** (ca. 21 mol%, ^{1}H).

NMR data of compound **11a**:

^{1}H NMR (600 MHz, 299 K, CD_2Cl_2): δ ^{1}H: 7.56 (d, $^{1}J_{PH}$ = 462.4 Hz, 1H, PH), 1.66 (d, $^{3}J_{PH}$ = 15.3 Hz, 27 H, tBu).

^{31}P{^{1}H} NMR (243 MHz, 299 K, CD_2Cl_2): δ ^{11}P: 47.9 ($\nu_{1/2}$ ~ 8 Hz).

^{31}P NMR (243 MHz, 299 K, CD_2Cl_2): δ ^{11}P: 47.9 (dm, $^{1}J_{PH}$ ~ 463 Hz).

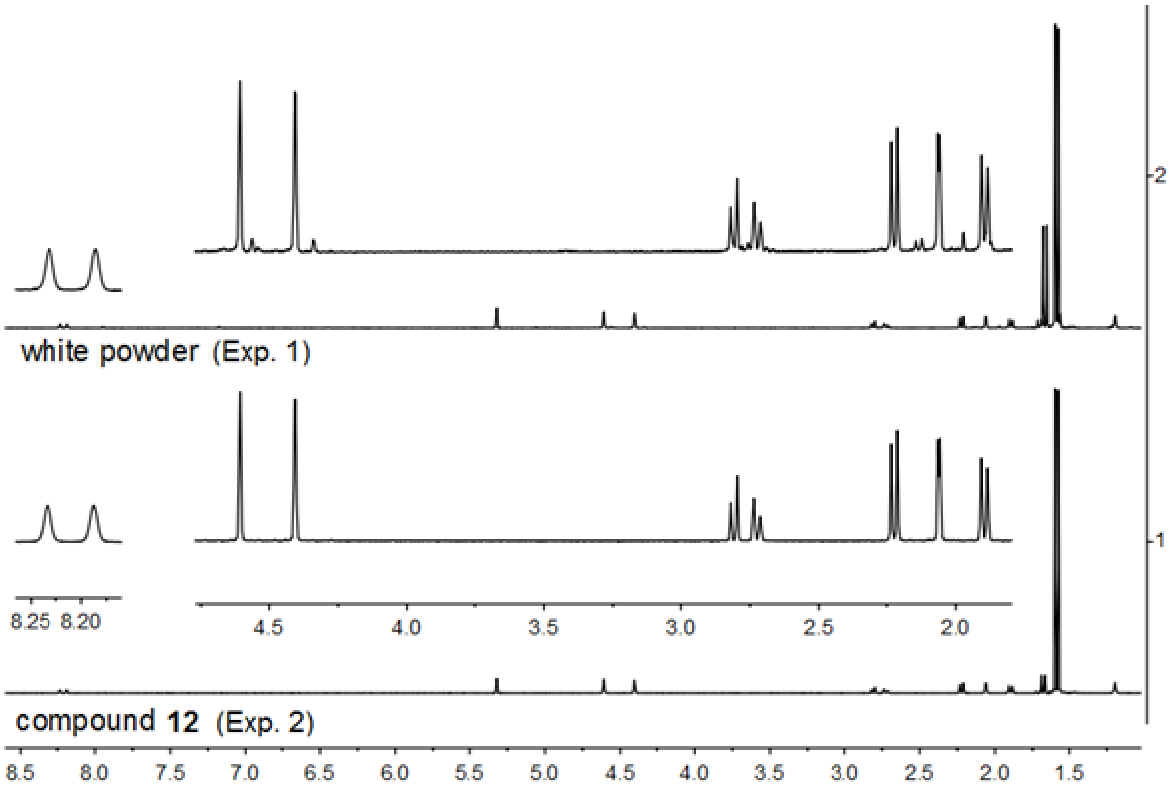

Figure S21. ^{1}H NMR (600 MHz, 299 K, CD_2Cl_2) spectra of (1) compound **12** (Experiment 2) and (2) the obtained white powder (Experiment 1).

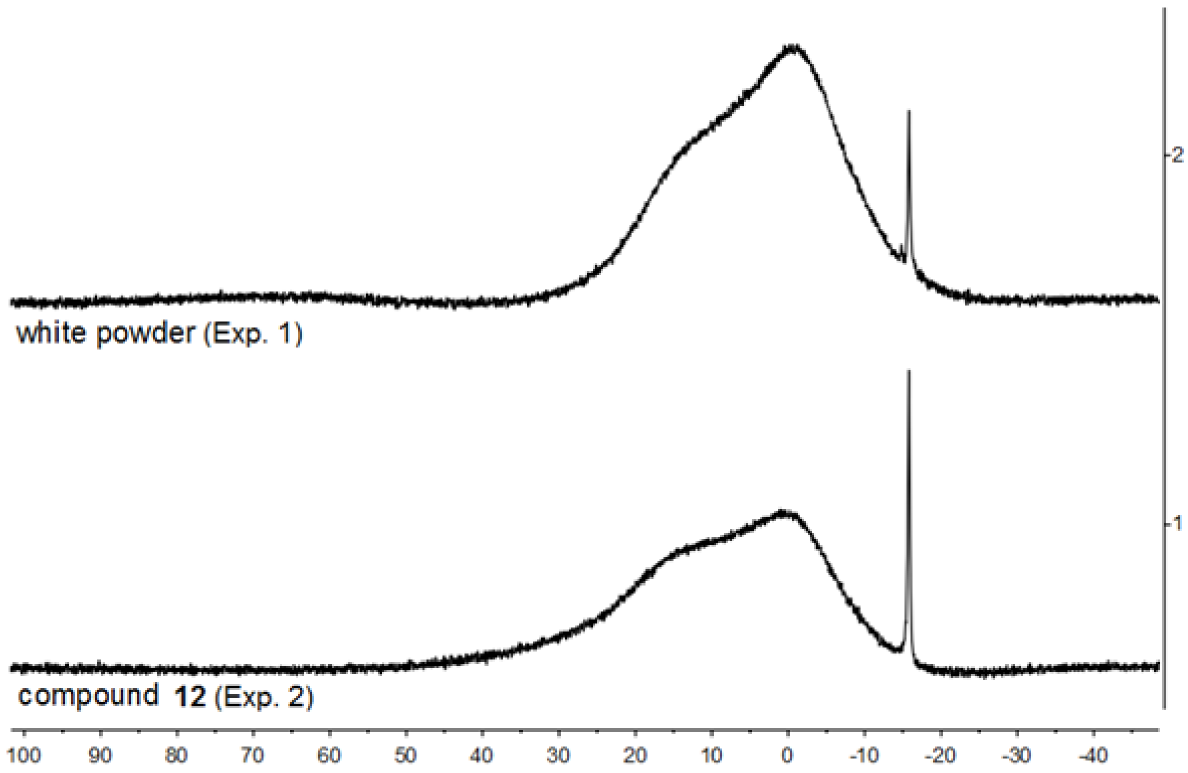

Figure S22.$^{11}B\{^1H\}$ NMR (192 MHz, 299 K, CD_2Cl_2) spectra of (1) compound **12** (Experiment 2) and (2) the obtained white powder (Experiment 1).

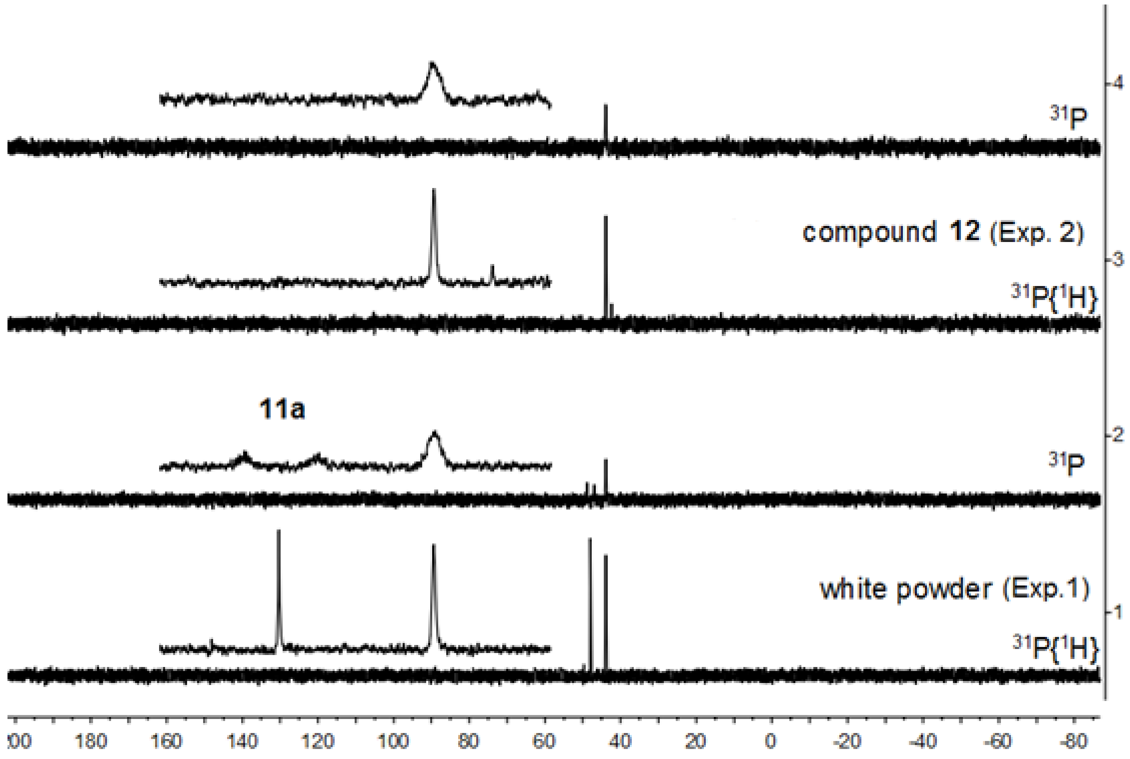

Figure S23. (1) $^{31}P\{^1H\}$ and (2) ^{31}P NMR (243 MHz, 299 K, CD_2Cl_2) spectra of the obtained white powder (Experiment 1) and (3) $^{31}P\{^1H\}$ and (4) ^{31}P NMR spectra of compound **12** (Experiment 2).

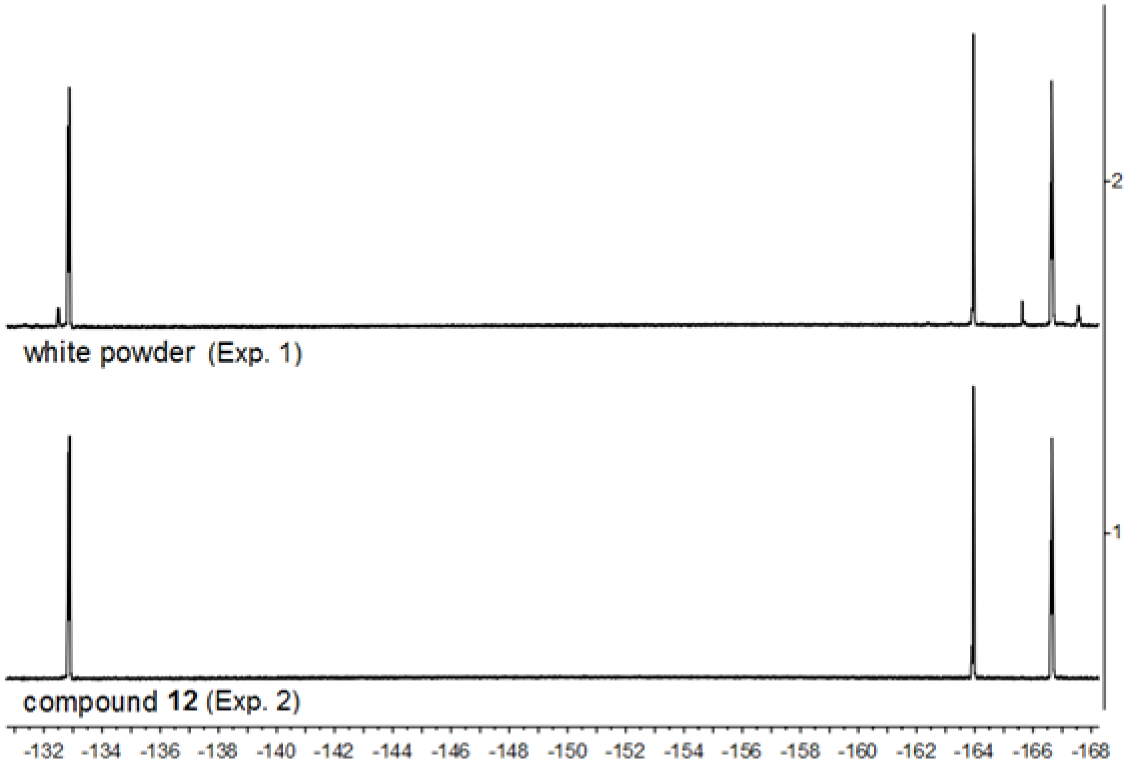

Figure S24.^{19}F NMR (564 MHz, 299 K, CD_2Cl_2) spectra of (1) compound **12** (Experiment 2) and (2) the obtained white powder (Experiment 1).

Experiment 2: isolation and characterization of compound **12**

Scheme S6.

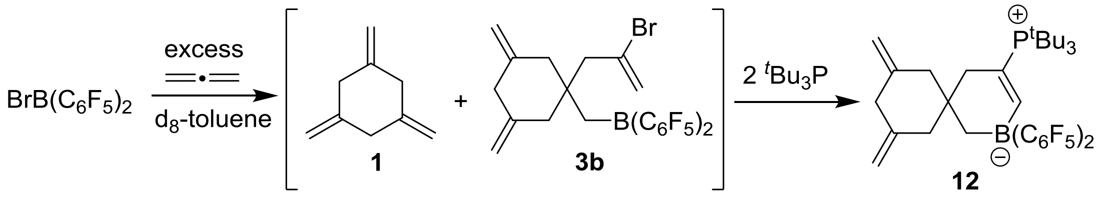

$BrB(C_6F_5)_2$ (150 mg, 0.357 mmol) was dissolved in d_8-toluene (2.0 mL) in a Schlenk tube. After carefully evacuating the Schlenk tube, the solution was exposed to allene gas for several minutes at room temperature. Then the resulting reaction mixture was stirred for 24 hours at room temperature. Subsequently, $^tBu_3P$ (144 mg, 0.714 mmol) was added. After stirring the reaction mixture for 24 hours at room temperature, all the volatiles were removed in vacuo and pentane (1 mL) was added to the residual oil. Then CH_2Cl_2 was added dropwise to the stirred mixture until it became a suspension. The solution was removed by filtration. Drying of the remaining solid in vacuo gave compound **12** (165 mg, 0.234 mmol, 65%) as a white powder.

Anal. Calc. for $C_{36}H_{42}BF_{10}P$: C, 61.20; H, 5.99. Found: C, 60.53; H, 5.93.

NMR data of compound **12**:

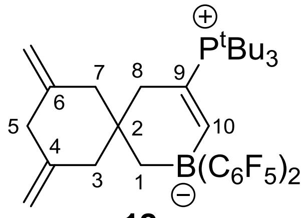

12

^{1}H NMR (600 MHz, 299 K, CD_2Cl_2): δ ^{1}H: 8.21 (d, $^3J_{PH}$ = 27.8 Hz, 1H, BCH=), [4.61, 4.41](each m, each 2H, 4,6-CH_2=), [2.81, 2.72](each dm, $^2J_{HH}$ = 14.0 Hz, each 1H, 5-CH_2), [2.22, 1.90](each d, $^2J_{HH}$ = 13.0 Hz, each 2H, 3,7-CH_2), 2.06 (d, $^4J_{PH}$ = 4.5 Hz , 2H, 8-CH_2), 1.58 (d, $^3J_{PH}$ = 13.2 Hz, 27 H, tBu), 1.20 (s, 2H, BCH_2).

^{13}C{^{1}H} NMR (151 MHz, 299 K, CD_2Cl_2): δ ^{13}C: 177.0 (br m, BCH=), 148.3 (dm, $^1J_{FC}$ ~ 240 Hz, C_6F_5), 147.7 (4,6-C=), 138.0 (dm, $^1J_{FC}$ ~ 240 Hz, C_6F_5), 137.0 (dm, $^1J_{FC}$ ~ 250 Hz, C_6F_5), 128.8 (i-C_6F_5), 118.9 (d, $^1J_{PC}$ = 37.0 Hz, PC=), 108.4 (4,6-CH_2=), 48.0 (3,7-CH_2), 44.4 (5-CH_2), 43.6 (d, $^2J_{PC}$ = 9.3 Hz, 8-CH_2), [41.5 (d, $^1J_{PC}$ = 28.0 Hz), 31.8](tBu), 40.7 (d, $^3J_{PC}$ = 6.1 Hz, 2-C), 32.8 (br m, BCH_2).

^{19}F NMR (564 MHz, 299 K, CD_2Cl_2): δ ^{19}F: [–132.9 (m, 2F, *o*), –163.9 (t, $^3J_{FF}$ = 21.0 Hz, 1F, *p*), –166.6 (m, 2F, *m*)](C_6F_5)[$\Delta\delta^{19}F_{m,p}$ = 2.7].

^{11}B{^{1}H} NMR (192 MHz, 299 K, CD_2Cl_2): δ ^{11}B: –15.8 ($\nu_{1/2}$ ~ 60 Hz).

^{31}P{^{1}H} NMR (243 MHz, 299 K, CD_2Cl_2): δ ^{11}P: 43.9 ($\nu_{1/2}$ ~ 30 Hz).

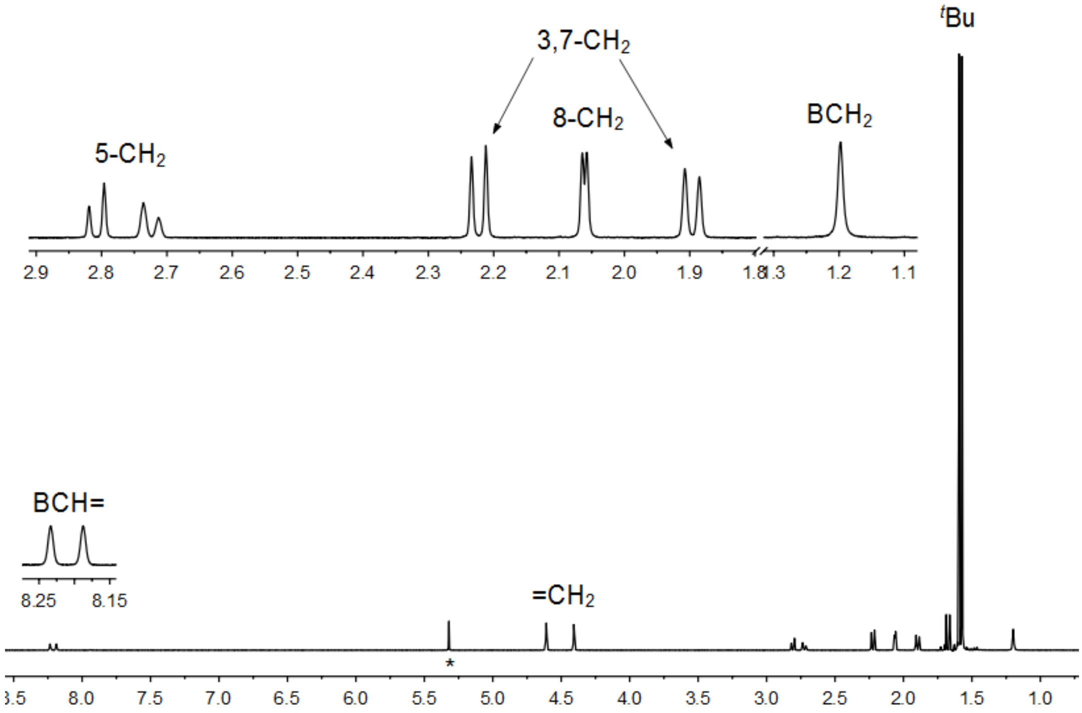

Figure S25. ^{1}H NMR (600 MHz, 299 K, CD_2Cl_2*) spectrum of compound **12**.

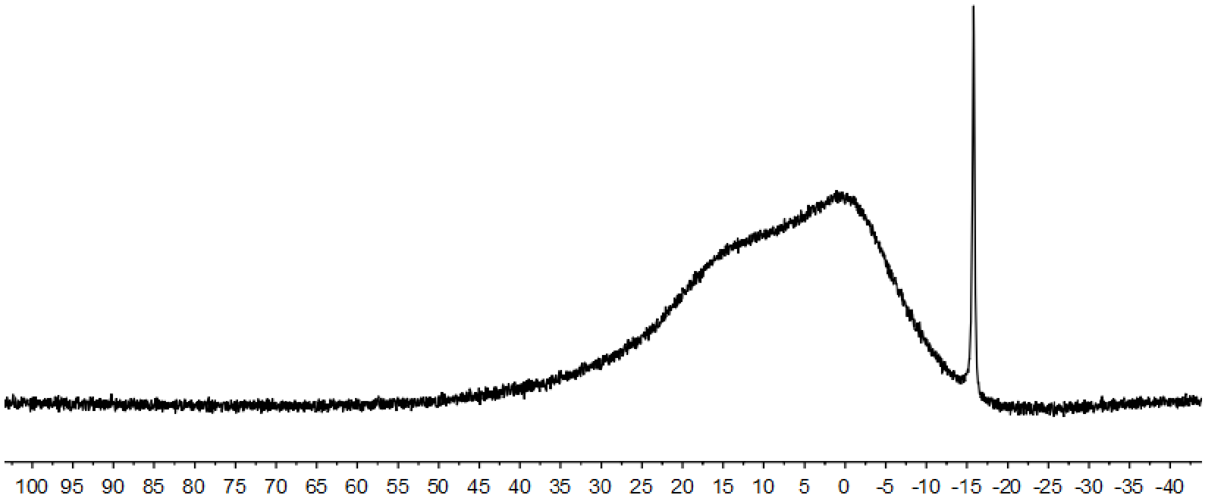

Figure S26.^{11}B{^{1}H} NMR (192 MHz, 299 K, CD_2Cl_2) spectrum of compound **12**.

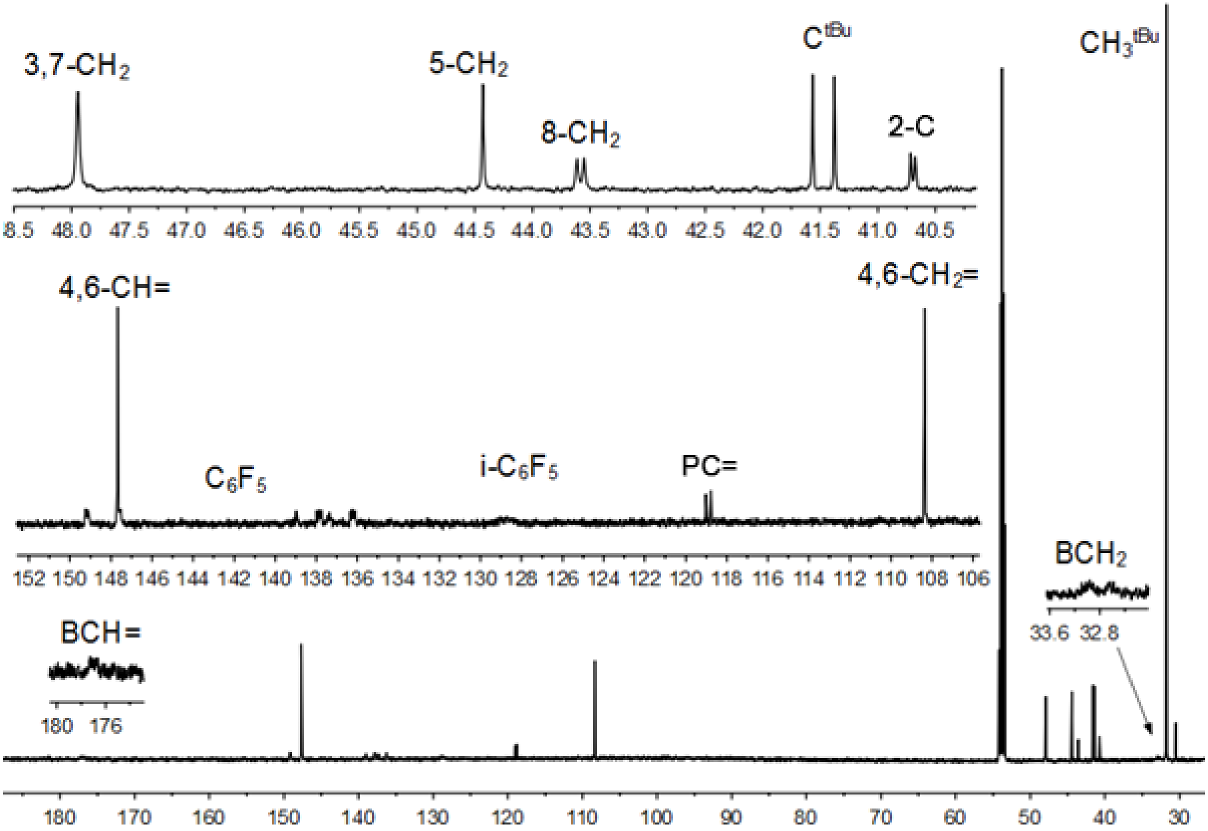

Figure S27. ^{13}C{^{1}H} NMR (151 MHz, 299 K, CD_2Cl_2) spectrum of compound **12**.

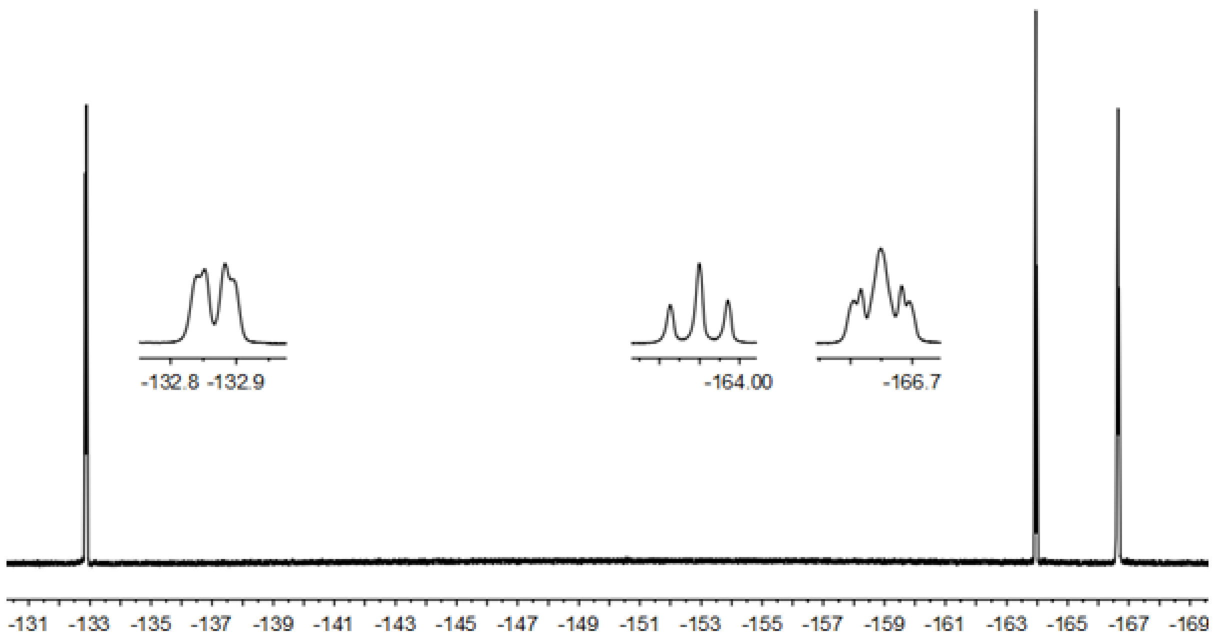

Figure S28. ^{19}F NMR (564 MHz, 299 K, CD_2Cl_2) spectrum of compound **12**.

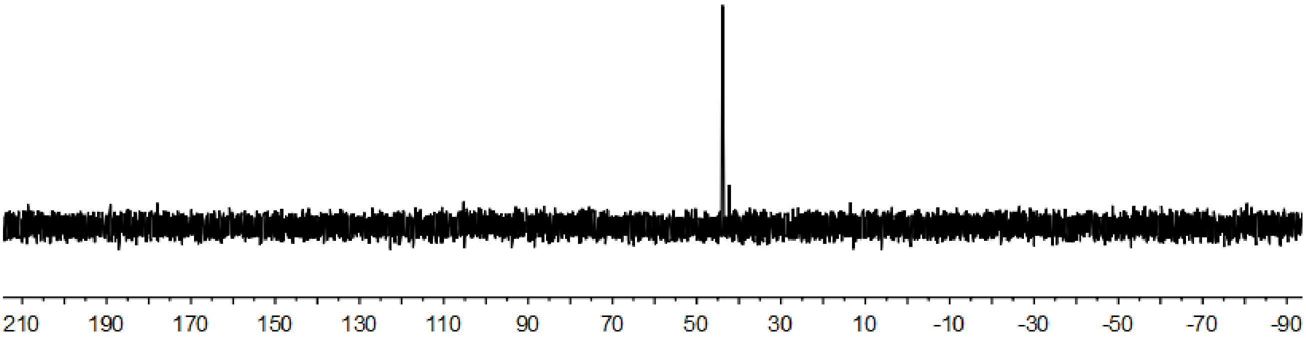

Figure S29. $^{31}P\{^{1}H\}$ NMR (243 MHz, 299 K, CD_2Cl_2) spectrum of compound **12**.

Single crystals suitable for the X-ray crystal structure analysis were obtained from the diffusion of pentane vapor to a solution of the white powder in CH_2Cl_2 at room temperature.

X-ray crystal structure analysis of compound 12 (erk9169): formula $C_{36}H_{42}BF_{10}P$, M = 706.48, colourless crystal, 0.13 x 0.13 x 0.03 mm, a = 9.9507(3) Å, b = 11.7835(3) Å, c = 16.0070(5) Å, α = 71.517(1)°, β = 86.117(1)°, γ = 70.299(1)°, V = 1674.26(8) Å^3, ρ_{calc} = 1.401 gcm^{-3}, μ = 0.164 mm^{-1}, empirical absorption correction (0.979 ≤ T ≤ 0.995), Z = 2, triclinic, space group $P\bar{1}$ (No. 2), λ = 0.71073 Å, T = 173(2) K, ω and ϕ scans, 15352 reflections collected (±h, ±k, ±l), 5781 independent (R_{int} = 0.047) and 4799 observed reflections [I>2σI)], 442 refined parameters, R = 0.055, wR^2 = 0.127, max. (min.) residual electron density 0.32 (-0.28) e.Å^{-3}, hydrogen atoms were calculated and refined as riding atoms. CCDC deposition number 1862535.

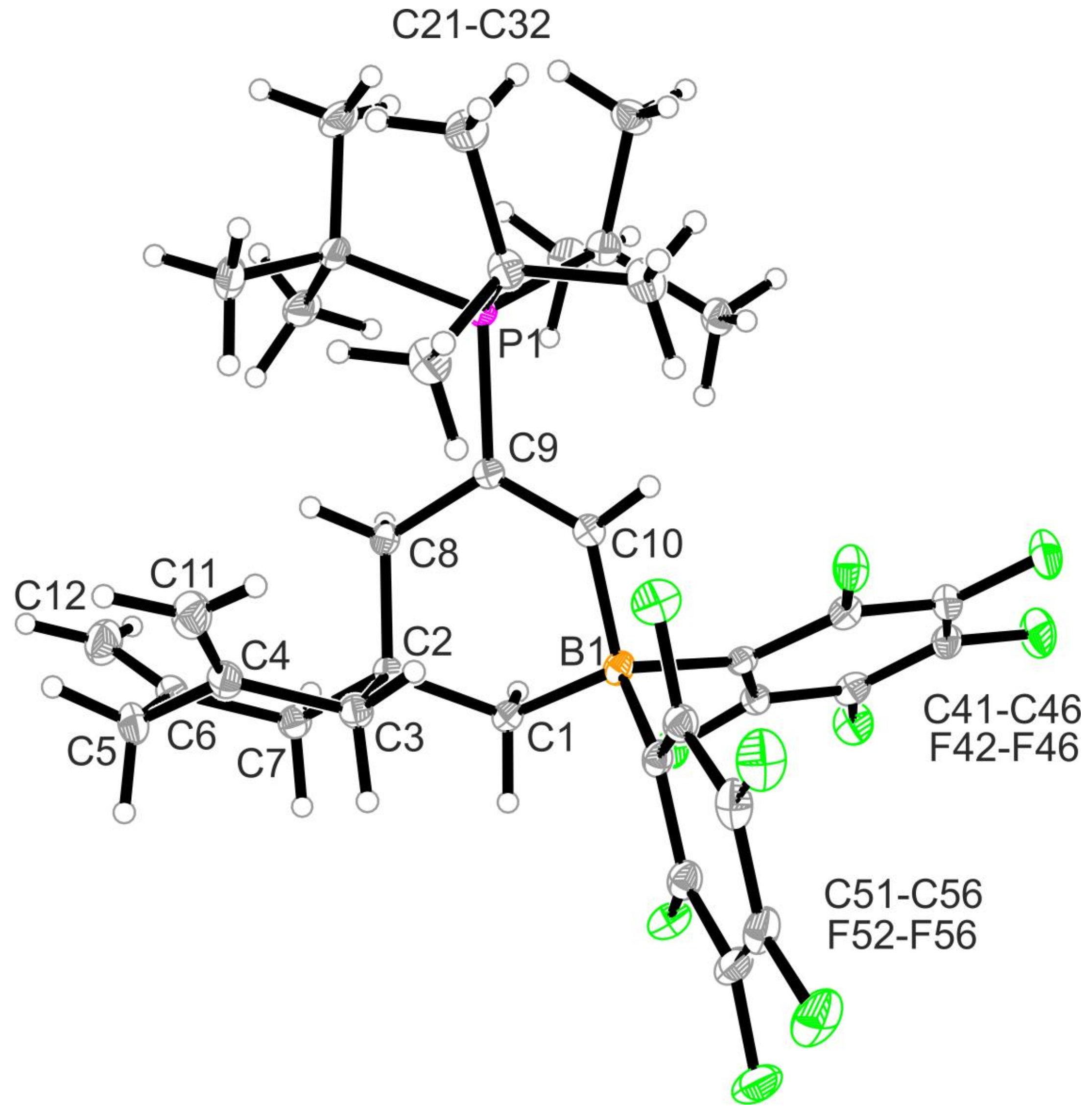

Figure S30. Crystal structure of compound **12** (thermal ellipsoids: 30% probability)

Experiment 3: compound **11b**

Scheme S7.

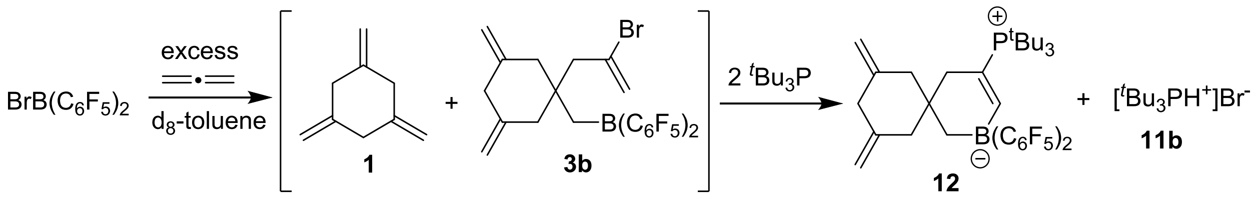

$BrB(C_6F_5)_2$ (50 mg, 0.119 mmol) was dissolved in d_8-toluene (2.0 mL) in a Schlenk tube. After carefully evacuating the Schlenk tube, the solution was exposed to allene gas for several minutes at

room temperature. Then the resulting reaction mixture was stirred for 24 hours at room temperature. Subsequently, $^tBu_3P$ (144 mg, 0.714 mmol) was added. After stirring the reaction mixture for 24 hours at room temperature, all the volatiles were removed in vacuo and pentane (1 mL) was added to the residual oil. Then CH_2Cl_2 was added dropwise to the stirred mixture to give a suspension. The liquid was separated by filtration. Drying of the remaining solid in vacuo gave compound **12** (58 mg, 0.082 mmol, 69%) as a white powder. The filtrate was dried in vacuo, dissolved in CD_2Cl_2 and characterized by NMR experiments.

NMR data of compound **11b** from the dried filtrate:

^{1}H NMR (600 MHz, 299 K, CD_2Cl_2): δ ^{1}H: 8.39 (d, $^1J_{PH}$ = 470.7 Hz, 1H, PH), 1.65 (d, $^3J_{PH}$ = 15.1 Hz, 27 H, tBu).

^{31}P{^{1}H} NMR (243 MHz, 299 K, CD_2Cl_2): δ ^{11}P: 41.4 ($\nu_{1/2}$ ~ 5Hz).

^{31}P NMR (243 MHz, 299 K, CD_2Cl_2): δ ^{11}P: 41.4 (dm, $^1J_{PH}$ ~ 470 Hz).

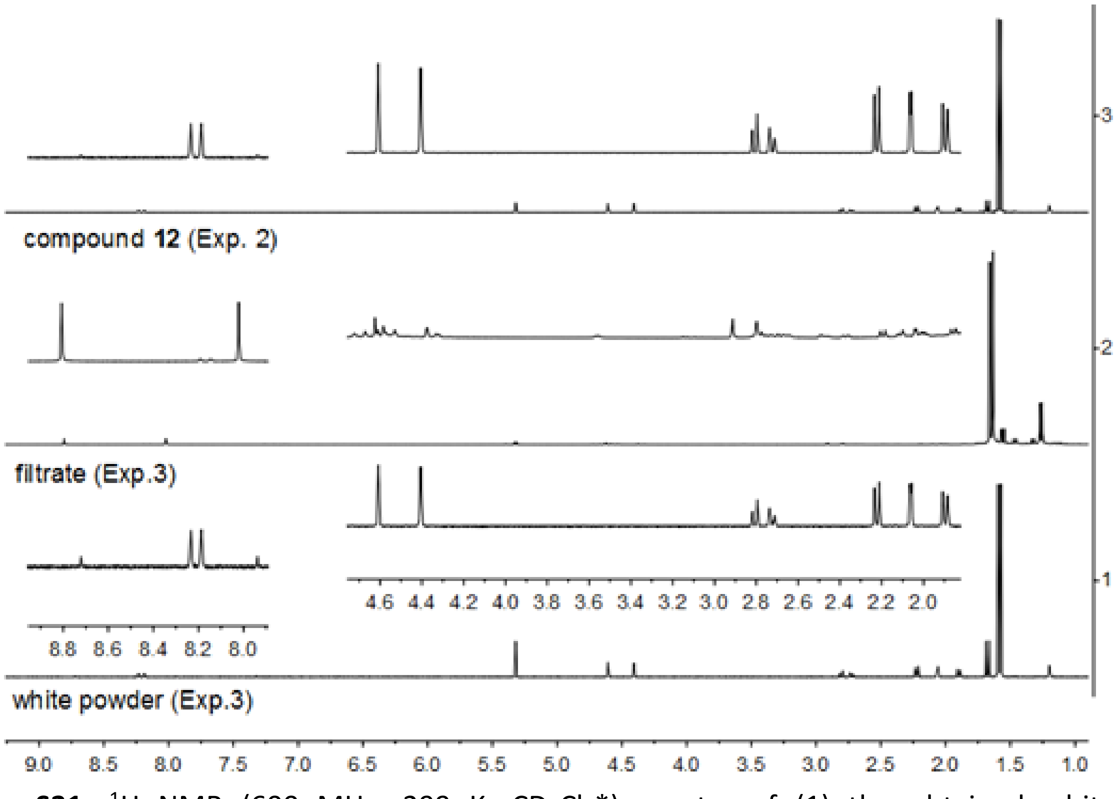

Figure S31. ^{1}H NMR (600 MHz, 299 K, CD_2Cl_2*) spectra of (1) the obtained white powder (Experiment 3), (2) the dried filtrate of Experiment 3 and (3) compound **12** (Experiment 2).

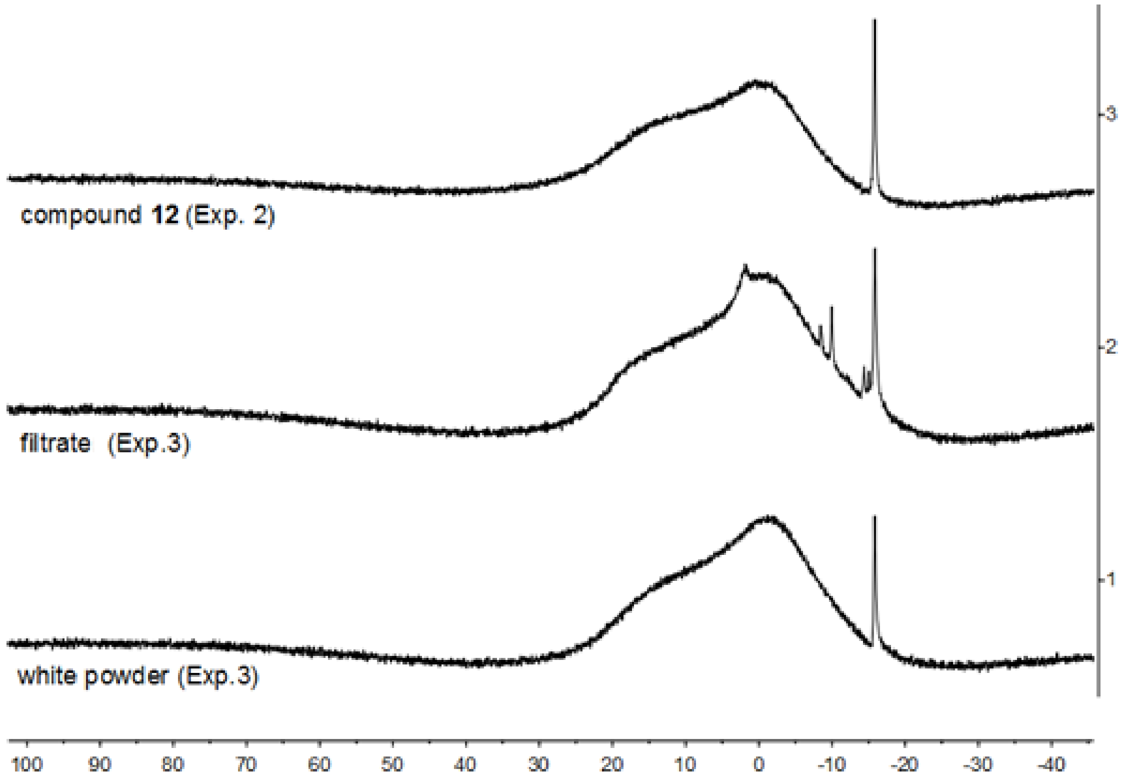

Figure S32.$^{11}B\{^{1}H\}$ NMR (192 MHz, 299 K, CD_2Cl_2) spectra of (1) the obtained white powder (Experiment 3), (2) the dried filtrate of Experiment 3, and (3) compound **12** (Experiment 2).

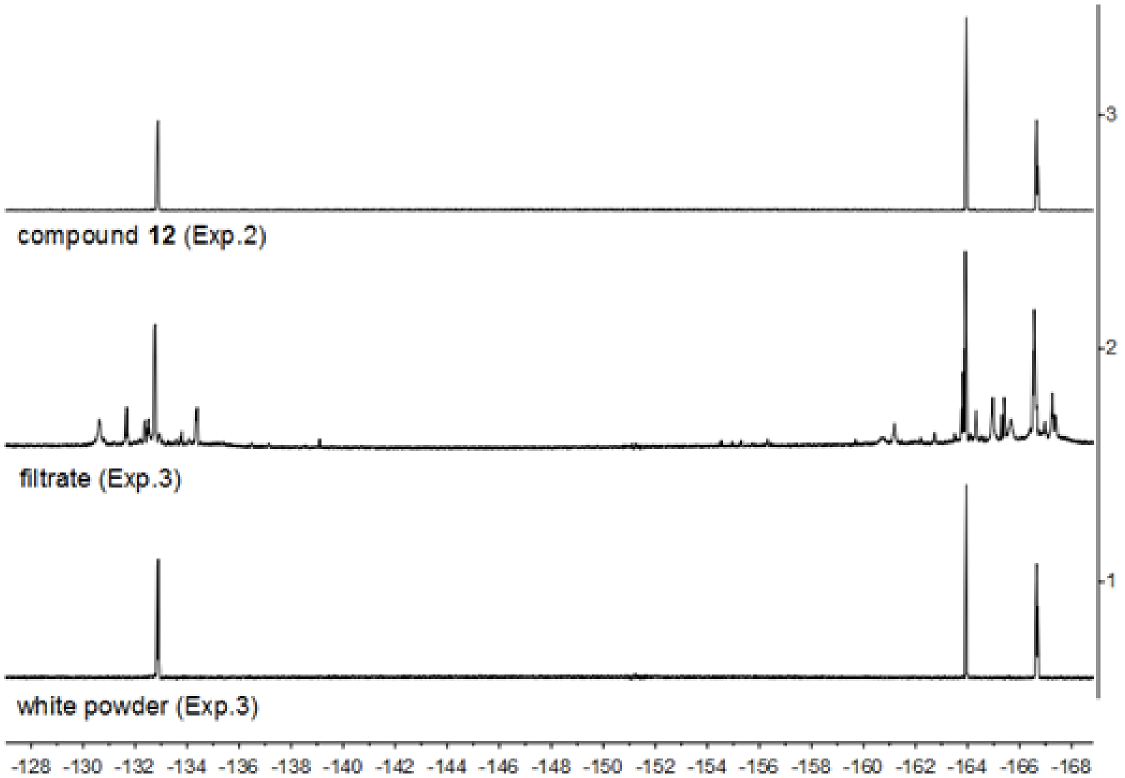

Figure S33.^{19}F NMR (564 MHz, 299 K, CD_2Cl_2) spectra of (1) the obtained white powder (Experiment 3), (2) the dried filtrate of Experiment 3, and (3) compound **12** (Experiment 2).

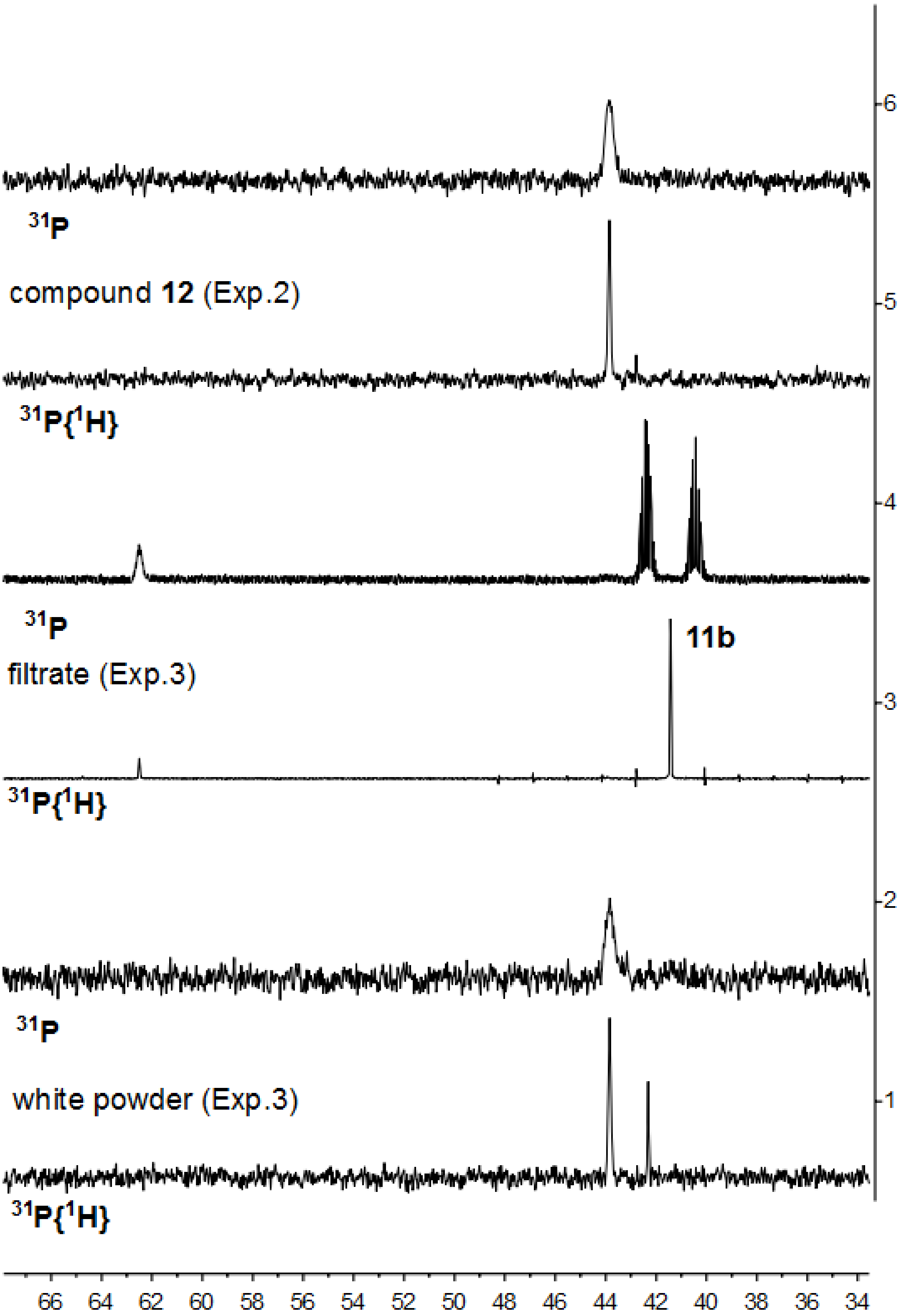

Figure S34. (1) ^{31}P{^{1}H} and (2) ^{31}P NMR (243 MHz, 299 K, CD_2Cl_2) spectra of the obtained white powder (Experiment 3), (3) ^{31}P{^{1}H} and (4) ^{31}P NMR spectra of the dried filtrate of Experiment 3, and (5) ^{31}P{^{1}H} and (6) ^{31}P NMR spectra of compound **12** (Experiment 2).

F) $XB(C_6F_5)_2$ (X = Cl, Br) catalyzed cyclotrimerization of substituted allenes **13**

Scheme S8.

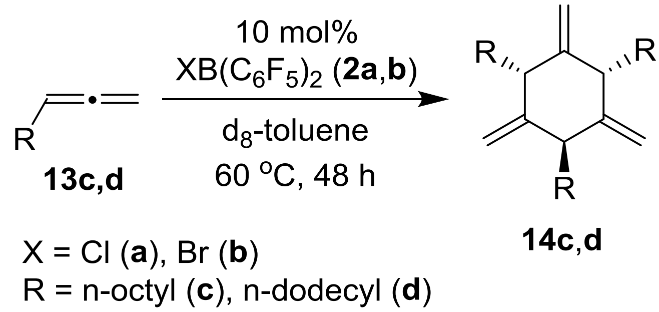

General procedure: borane **2** (0.060 mmol) and substrate **13** (0.600 mmol) were dissolved in d_8-toluene (ca. 1 mL) in a NMR tube. After flame-sealing the NMR tube, it was placed in a steel autoclave and heated at 60 °C for 48 h. Then the tube was opened and all the volatile was removed in vacuo. The residue was purified through column chromatography (silica gel, pentane) giving the corresponding final product **14**.

Table S1. Results of $XB(C_6F_5)_2$ (X: Cl, Br) catalyzed cyclotrimerization of substituted allenes **13**.[a]

Entry	Substrate (mg/mmol)	Borane (mg/mmol)	Yield (mg/mmol)[b]	Yield (%)[b]
1	**13c** (91.2 / 0.600)	**2a** (22.8 / 0.060)	**14c** (40.4 / 0.009)	44
2	**13c** (91.2 / 0.600)	**2b** (25.4 / 0.060)	**14c** (42.0 / 0.009)	46
3	**13d** (126 / 0.600)	**2a** (22.8 / 0.060)	**14d** (50.2 / 0.008)	40
4	**13d** (126 / 0.600)	**2b** (25.4 / 0.060)	**14d** (68.1 / 0.011)	54

[a]Reaction conditions: 60 °C, 48 h; [b] isolated yield.

Entry 1:

Scheme S9.

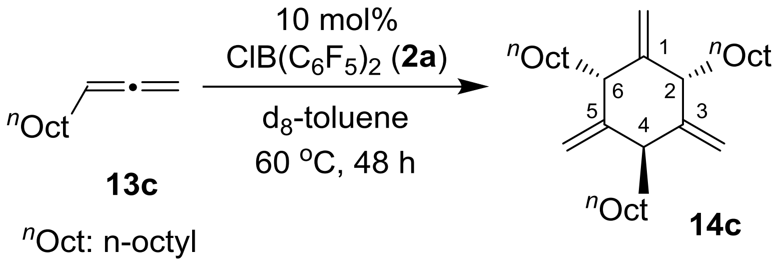

Following the general procedure $ClB(C_6F_5)_2$ (**2a**) (22.8 mg, 0,060 mmol) and n-octylallene (**13c**) (91.2 mg, 0.600 mmol) were used as starting materials. Compound **14c** (40.4 mg, 0.009 mmol, 44%) was isolated as colorless oil.

HRMS: m/z Calc. for $C_{33}H_{60}$ $[M+Ag]^+$: 563.37405. Found 563.37454.

^{1}H NMR (600 MHz, 299 K, CD_2Cl_2): δ ^{1}H: 4.67 (s, 2H, 1-CH_2=), [4.66, 4.63](each m, each 2H, 3,5-CH_2=), 2.90 (t, $^3J_{HH}$ = 7.1 Hz, 1H, 4-CH), 2.86 (t, $^3J_{HH}$ = 7.1 Hz, 2H, 2,6-CH), 1.64 (m, 2H, 4-CH_2), [1.64, 1.58](each m, each 2H, 2,6-CH_2), 1.31-1.28 (m, 36H, CH_2^{octyl}), 0.89 (t, $^3J_{HH}$ = 7.3 Hz, 9H, CH_3).

^{13}C{^{1}H} NMR (151 MHz, 299 K, CD_2Cl_2): δ ^{13}C: 154.1 (1-C=), 154.0 (3,5-C=), 108.0 (1-CH_2=), 105.7 (3,5-CH_2=), 51.6 (2,6-CH), 46.2 (4-CH), 34.0 (2,6-CH_2), 30.7 (4-CH_2), [32.3, 30.2, 30.0, 29.9 (br m), 29.7 (br m), 28.3, 27.9, 23.1](CH_2^{octy}), 14.3 (CH_3).

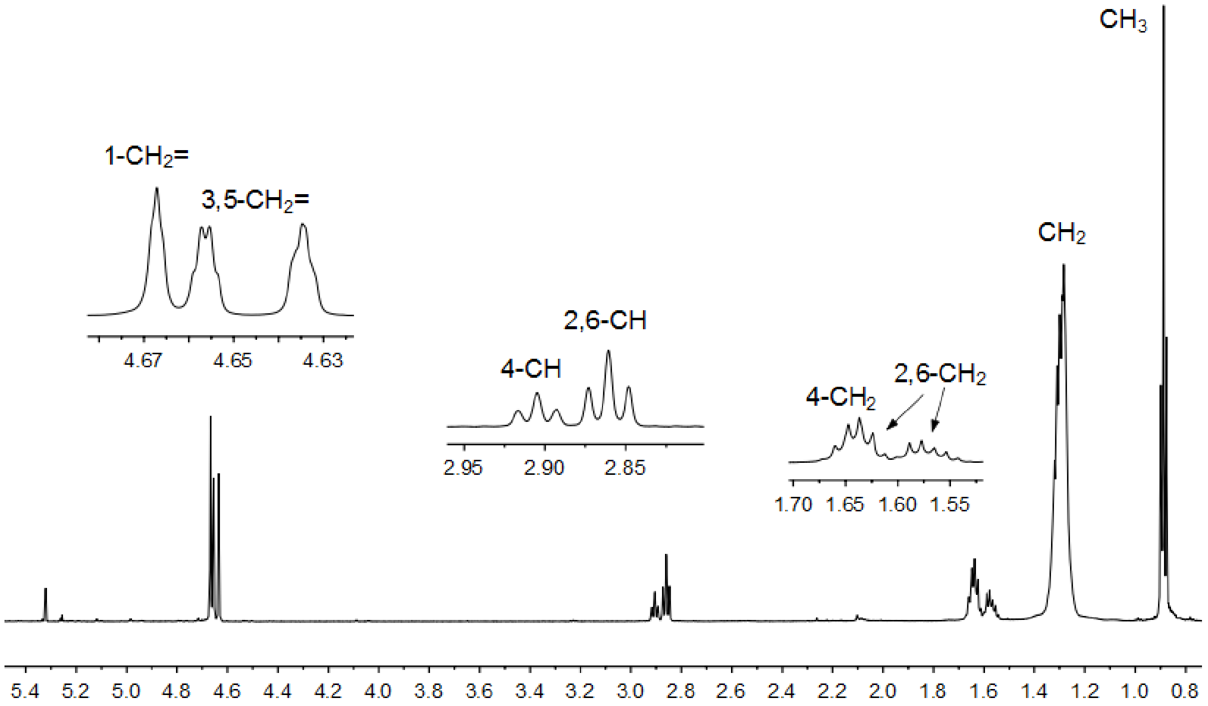

Figure S35. ^{1}H NMR (600 MHz, 299 K, CD_2Cl_2) spectrum of isolated compound **14c**.

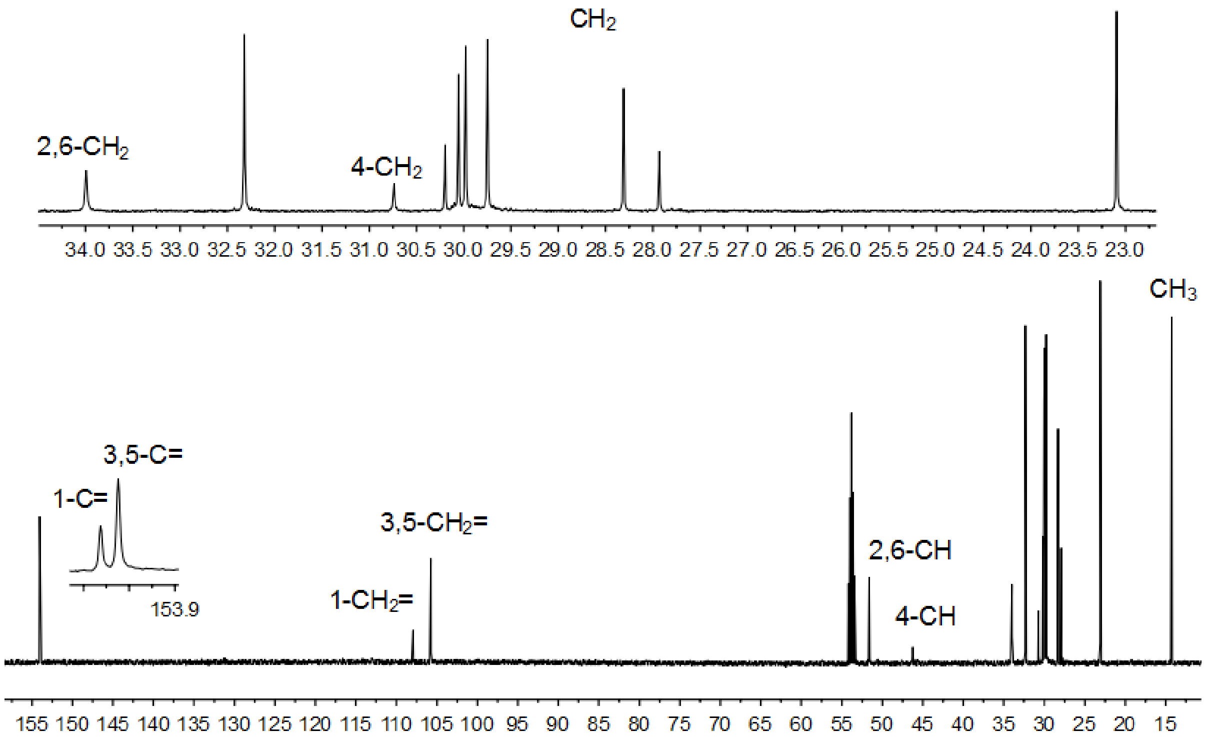

Figure S36. $^{13}C\{^{1}H\}$ NMR (151 MHz, 299 K, CD_2Cl_2) spectrum of isolated compound **14c**.

Entry 2:

Scheme S10.

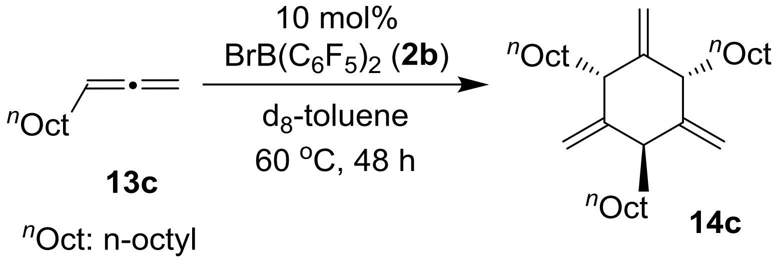

Following the general procedure $BrB(C_6F_5)_2$ (**2b**) (25.4 mg, 0,060 mmol) and n-octylallene (**13c**) (91.2 mg, 0.600 mmol) were used as starting materials. A colorless oil (42.0 mg, 0.009 mmol, 46%) was isolated as colorless oil.

HRMS: m/z Calc. for $C_{33}H_{60}$ $[M+Ag]^+$: 563.37405. Found 563.37497.

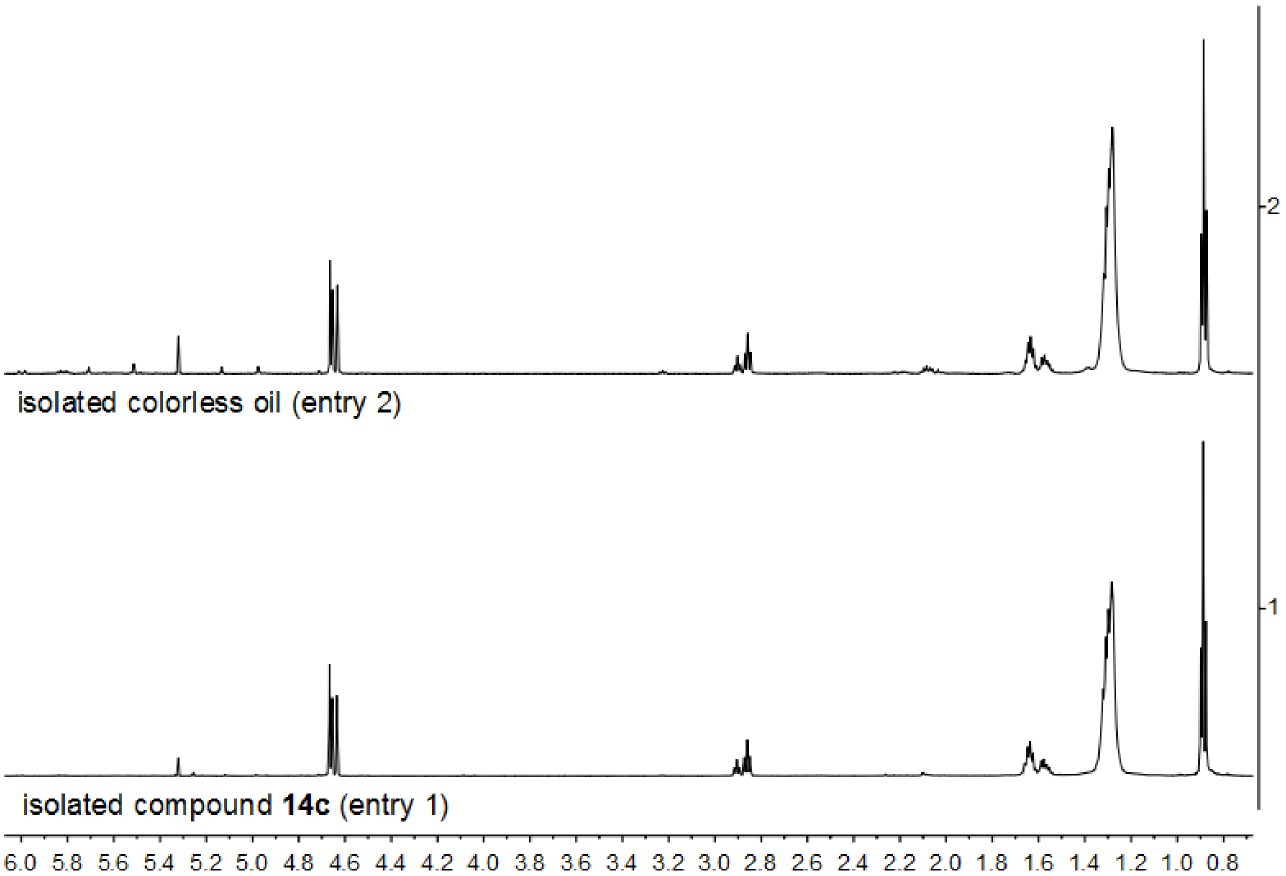

Figure S37. ^{1}H NMR (600 MHz, 299 K, CD_2Cl_2) spectra of (1) isolated compound **14c** (entry 1) and (2) isolated colorless oil (entry 2).

Entry 3:

Scheme S11.

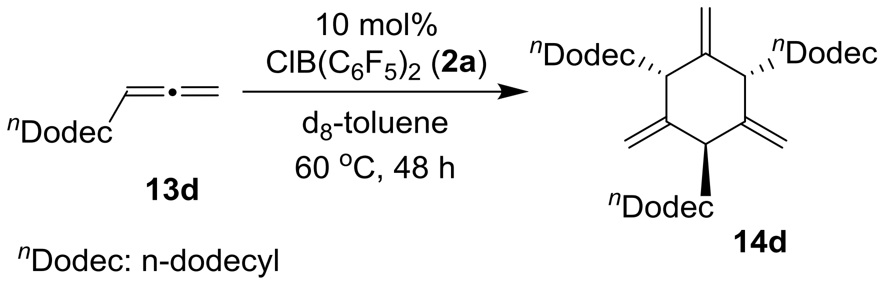

Following the general procedure $ClB(C_6F_5)_2$ (**2a**) (22.8 mg, 0,060 mmol) and n-dodecylallene (**13c**) (126 mg, 0.600 mmol) were used as starting materials. Compound **14d** (50.2 mg, 0.008 mmol, 40%) was isolated as coloerless oil.

HRMS: m/z Calc. for $C_{45}H_{84}$ $[M+Ag]^+$: 731.56185. Found 731.56279.

^{1}H NMR (600 MHz, 299 K, CD_2Cl_2): δ ^{1}H: 4.67 (s, 2H, 1-CH_2=), [4.66, 4.63](each m, each 2H, 3,5-

CH_2=), 2.90 (t, $^3J_{HH}$ = 7.1 Hz, 1H, 4-CH), 2.86 (t, $^3J_{HH}$ = 7.1 Hz, 2H, 2,6-CH), 1.64 (m, 2H, 4-CH_2), [1.64, 1.58](each m, each 2H, 2,6-CH_2), 1.32-1.27 (m, 60H, $CH_2^{dodecyl}$), 0.89 (t, $^3J_{HH}$ = 7.3 Hz, 9H, CH_3).

$^{13}C\{^1H\}$ NMR (151 MHz, 299 K, CD_2Cl_2): δ ^{13}C: 154.1 (1-C=), 154.0 (3,5-C=), 108.0 (1-CH_2=), 105.8 (3,5-CH_2=), 51.6 (2,6-CH), 46.2 (4-CH), 34.0 (2,6-CH_2), 30.7 (4-CH_2), [32.3, 30.2, 30.1-30.0 (br m), 28.3, 27.9, 23.1]($CH_2^{dodecyl}$), 14.3 (CH_3).

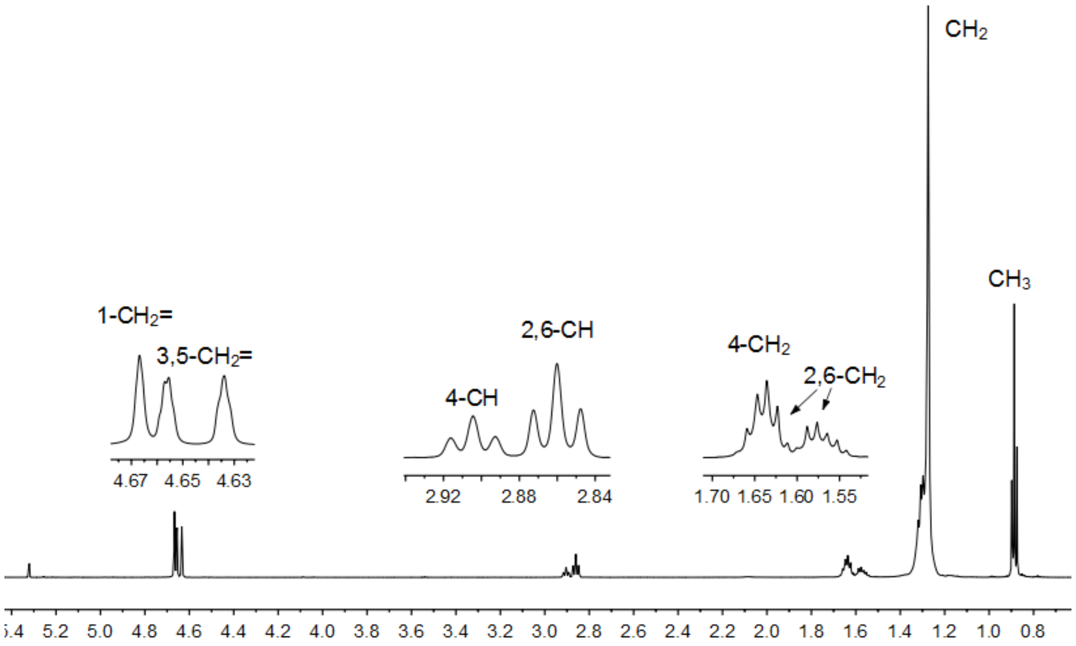

Figure S38. ^1H NMR (600 MHz, 299 K, CD_2Cl_2) spectrum of isolated compound **14d**.

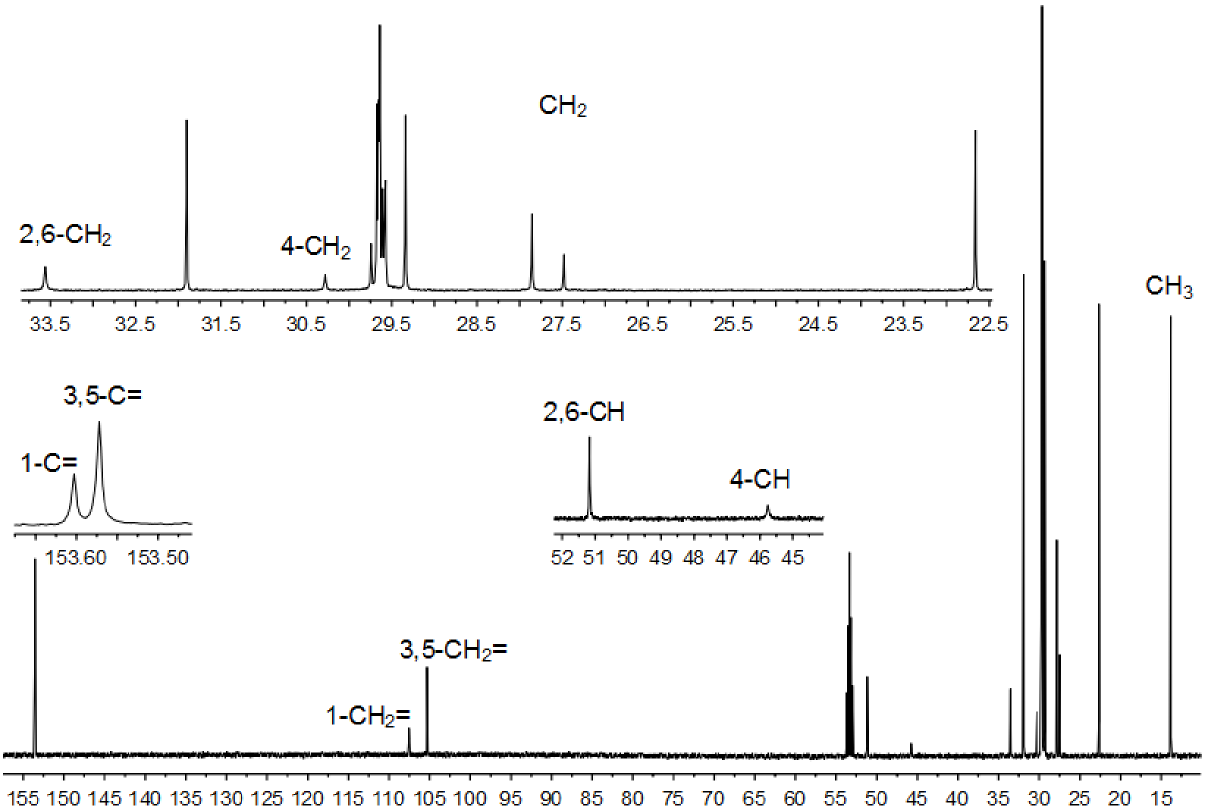

Figure S39. $^{13}C\{^{1}H\}$ NMR (151 MHz, 299 K, CD_2Cl_2) spectrum of isolated compound **14d**.

Entry 4:

Scheme S12.

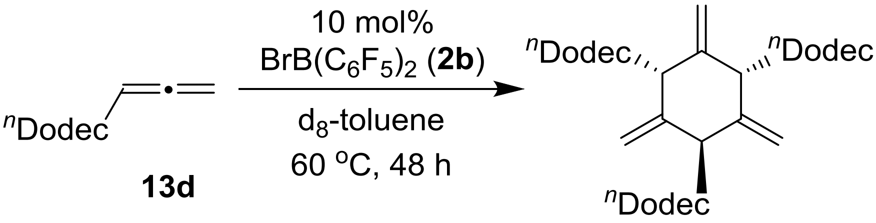

13d **14d**

^{n}Dodec: n-dodecyl

Following the general procedure $BrB(C_6F_5)_2$ (**2b**) (25.4 mg, 0,060 mmol) and n-dodecylallene (**13c**) (126 mg, 0.600 mmol) were used as starting materials. A colorless oil (68.1 mg, 0.011 mmol, 54%) was isolated.

HRMS: m/z Calc. for $C_{45}H_{84}$ $[M+Ag]^+$: 731.56185. Found 731.56310.

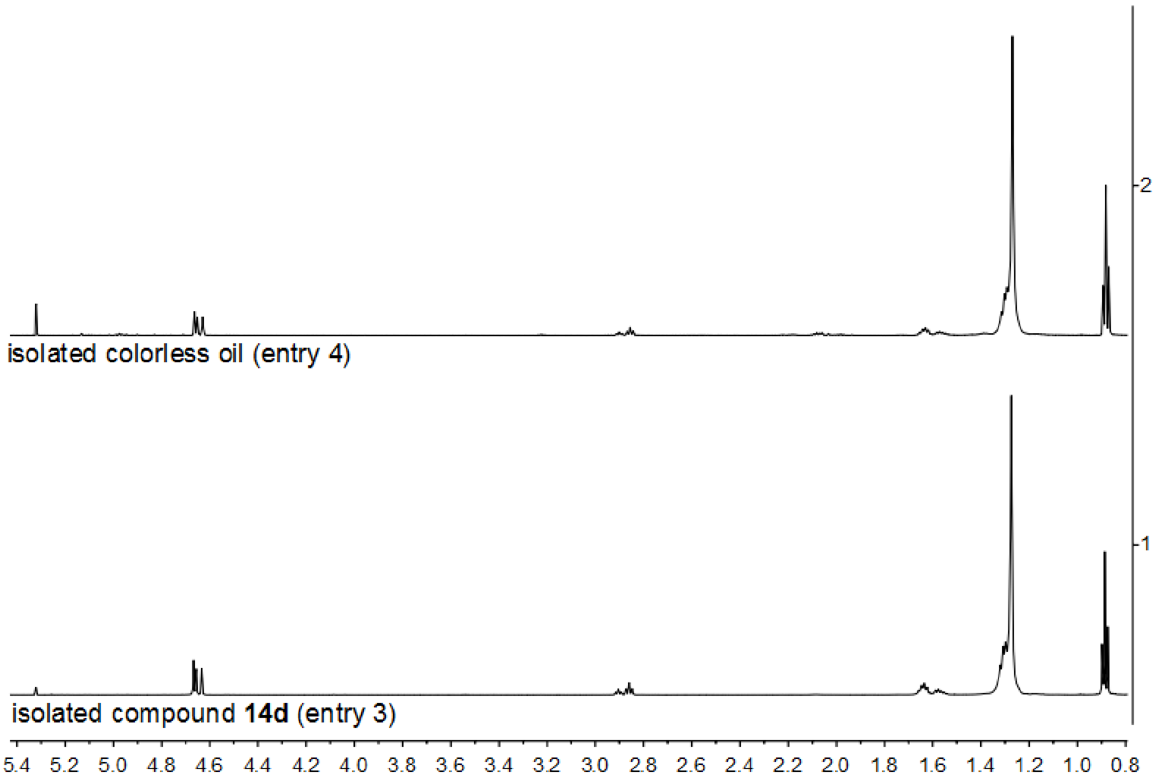

Figure S40. ^{1}H NMR (600 MHz, 299 K, CD_2Cl_2) spectra of (1) isolated compound **14d** (entry 3) and (2) isolated colorless oil (entry 4).

G) Isomerization of the cyclotrimer 1

Scheme S13.

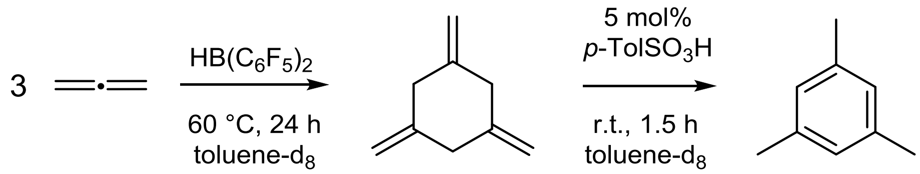

Step 1: $HB(C_6F_5)_2$ (20.8 mg, 0.06 mmol) was suspended in toluene-d$_8$ (0.7 mL) and the atmosphere removed. After applying allene gas for 2 min, the mixture was stirred for 10 min at room temperature. The now clear solution was transferred to a NMR tube, which was then flame-sealed and heated to 60 °C for 24 h in a steel autoclave. Then the solution was filtered through a small plug of silica and ferrocene (9.8 mg, 0.05 mmol) was added as an internal standard. The obtained solution was characterized by ^{1}H NMR experiment. A yield of 0.077 mmol was estimated by integration (^{1}H) of compound **1** in relation to the ferrocene standard.

NMR data of compound **1** in solution from **Step 1**:

^{1}H NMR (600 MHz, 299 K, toluene-d_8): δ ^{1}H: 4.59 (m, 1H, =CH_2), 2.75 (m, 1H, CH_2).

Step 2: Without further workup *p*-toluene sulfonic acid monohydrate (0.7 mg, 0.004 mmol; 5 mol%) was added to the solution from Step 1. After 1.5 h at room temperature, the ^{1}H NMR spectrum indicated complete conversion to mesitylene.

NMR data of mesitylene in solution from **Step 2**:

^{1}H NMR (600 MHz, 299 K, toluene-d_8): δ ^{1}H: 6.66 (m, 1H, ArH), 2.14 (m, 3H, CH_3).

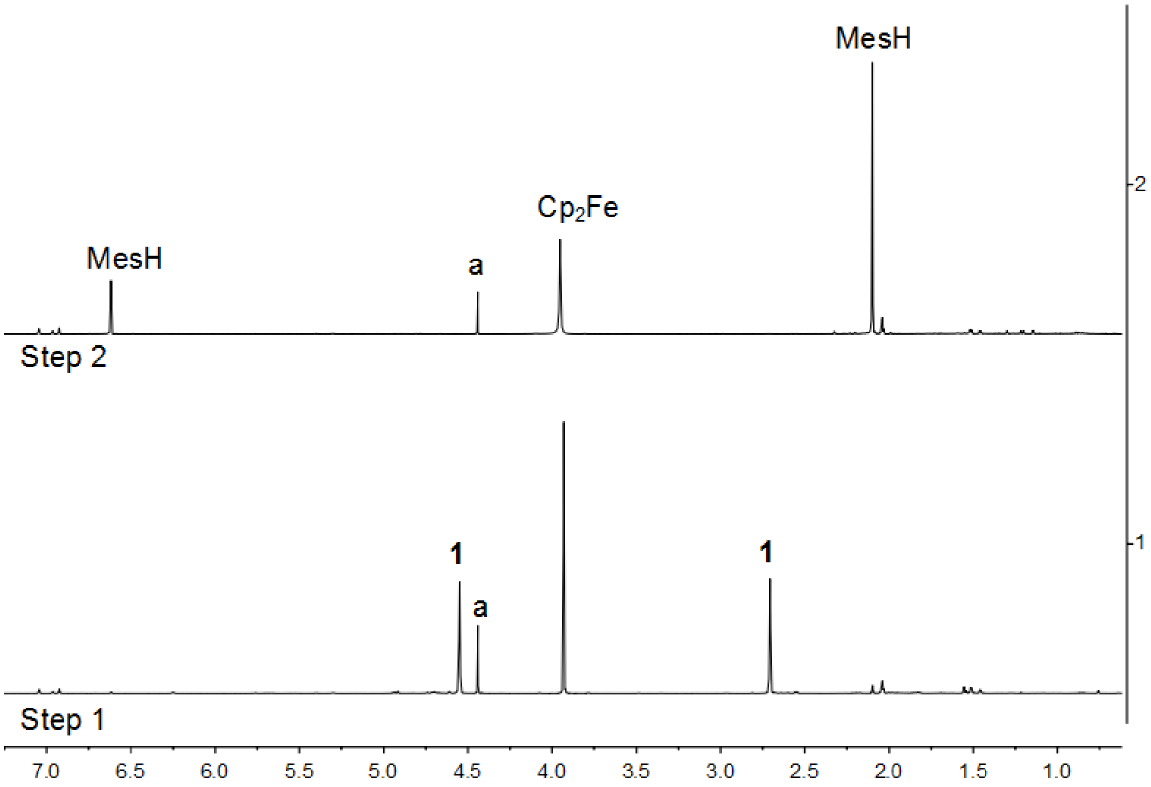

Figure S41. ^{1}H NMR (600 MHz, 299 K, toluene-d_8) spectra of the reaction mixture of Step 1 (spectrum 1) and Step 2 (spectrum 2).

H) Isomerization of the cyclotrimer **14c**

Experiment 1: (NMR scale)

Scheme S14.

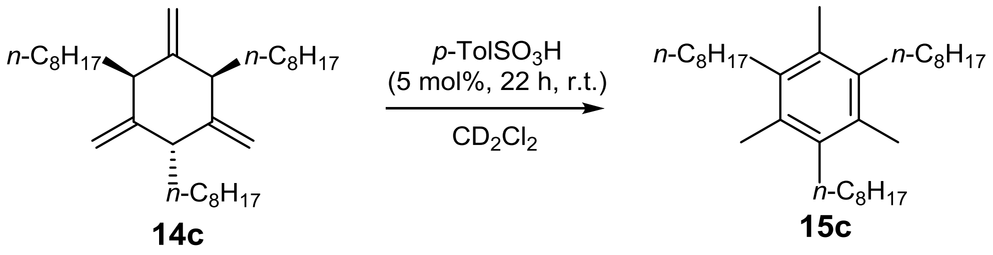

The cyclotrimer **14c** (11.0 mg, 0.024 mmol) was dissolved in CD_2Cl_2 (0.7 mL) and *p*-toluene sulfonic acid monohydrate (0.2 mL, 0.001 mmol; 0.0055 M in CD_2Cl_2) was added. Then the solution was sealed in a NMR tube. After 22 h at room temperature, the reaction mixture was characterized by ^{1}H NMR experiment.

Experiment 2: (preparative scale)

Scheme S15.

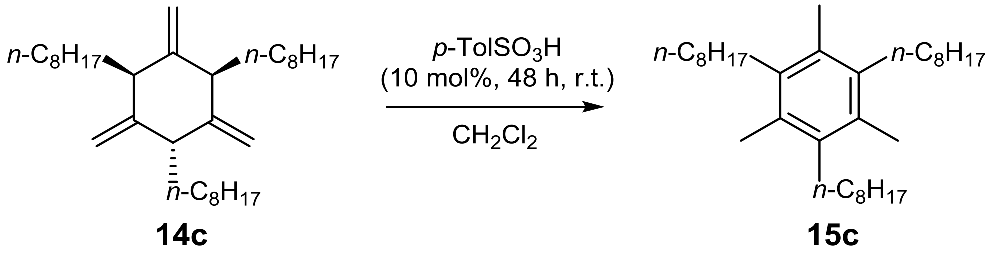

The cyclotrimer **14c** (86.0 mg, 0.19 mmol) was dissolved in CH_2Cl_2 (10 mL) and *p*-toluene sulfonic acid monohydrate (2 mg, 0.01 mmol; 5 mol%) was added. Then the solution was stirred for 24 h at room temperature, during which it took on a brownish color. After removal of all volatiles *in vacuo*, the ^{1}H NMR spectrum of the crude product indicated a conversion of 75%. The crude product was dissolved in CH_2Cl_2 (10 mL) and another portion of *p*-toluene sulfonic acid monohydrate (2 mg, 0.01 mmol; 5 mol%) was added. After stirring for additional 24 h at room temperature and removal of all volatiles *in vacuo*, the ^{1}H NMR spectrum of the crude product indicated full conversion. Then the crude product was dissolved in pentane (10 mL) and passed through a small plug of silica. After rinsing of the silica plug with pentane (30 mL), all volatiles were removed *in vacuo* and compound **15c** (72.0 mg, 0.15 mmol, 78%) was isolated as a yellowish oil.

HRMS: m/z Calc. for $C_{33}H_{60}$ $[M+Ag]^+$: 563.37405. Found 563.37445.

^{1}H NMR (600 MHz, 299 K, CD_2Cl_2): δ ^{1}H: 2.60 (m, 2H, CH_2^{Ar}), 2.22 (s, 3H, CH_3^{Ar}), 1.45-1.20 (m, 12H, CH_2), 0.89 (m, 3H, CH_3^{Oct}).

^{13}C{^{1}H} NMR (600 MHz, 299 K, CD_2Cl_2): δ ^{13}C: 137.8 and 131.5 (C^{Ar}), 32.3 (CH_2), 31.1 (CH_2^{Ar}), 30.7 (CH_2), 30.1 (CH_2), 29.9 (CH_2), 29.7 (CH_2), 23.1 (CH_2), 15.7 (CH_3^{Ar}), 14.3 (CH_3^{Oct}).

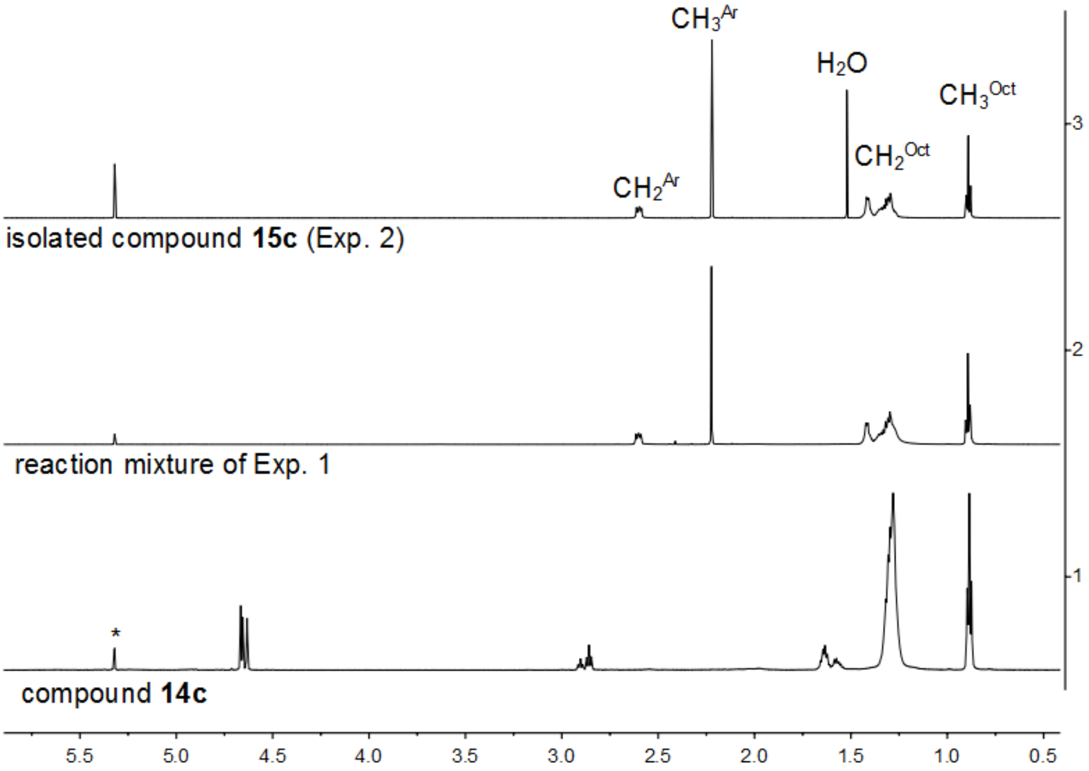

Figure S42. ^{1}H NMR (600 MHz, 299 K, CD_2Cl_2(*))spectra (1) of compound **14c**, (2) of the reaction mixture from Experiment 1 and (3) of isolated compound **15c** from Experiment 2.

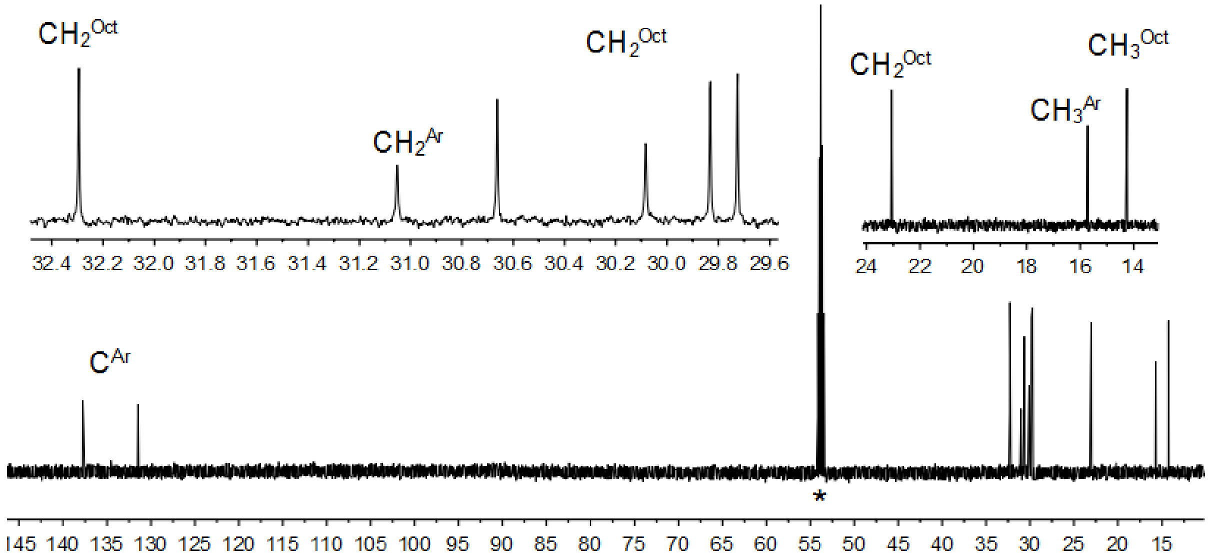

Figure S43. ^{13}C{^{1}H} NMR (151 MHz, 299 K, CD_2Cl_2(*)) spectrum of isolated compound **15c** from Experiment 2.

I) Isomerization of cyclotrimer **14e**

Experiment 1: (NMR scale)

Scheme S16.

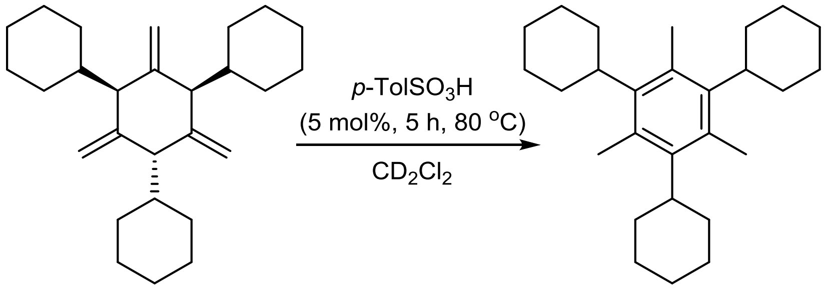

14e **15e**

The cyclotrimer **14e** (8.8 mg, 0.024 mmol) was dissolved in CD_2Cl_2 (0.7 mL) and *p*-toluene sulfonic acid monohydrate (0.2 mL, 0.001 mmol; 0.0055 M in CD_2Cl_2) was added. Then the solution was sealed in a NMR tube, which was then placed in a steel autoclave and heated up to 80 °C for 5 hours. The reaction mixture was then characterized by ^1H NMR experiment.

Experiment 2: (preparative scale)

Scheme S17.

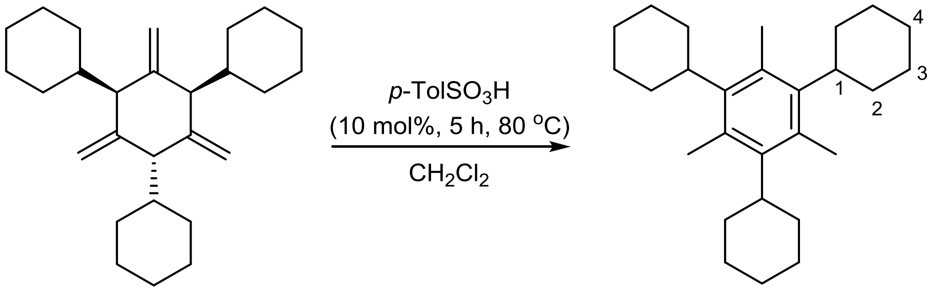

14e **15e**

The cyclotrimer (88.0 mg, 0.24 mmol) and *p*-toluene sulfonic acid monohydrate (4.6 mg, 0.024 mmol; 10 mol%) were mixed in CH_2Cl_2 (10 mL) in an ampule, which was subsequently placed in a steel autoclave. The autoclave was treated with argon (ca. 10 bar) and then heated up to 80 °C for 5 hours. After the autoclave was cooled down to room temperature, the argon was carefully released. A blue solution was obtained. All the volatile was removed by rotation evaporator and the residue was purified via column chromatography (silica gel, pentane) giving product **15e** (32.3 mg, 0.088 mmol, 37%) as white solid. Crystals suited for the X-ray crystal structure analysis were obtained from a solution of compound **15e** in dichloromethane at -35 °C.

HRMS: m/z Calc. for $C_{27}H_{42}$ $[M+Ag]^+$: 473.23320. Found 473.23366.

^{1}H NMR (600 MHz, 299 K, CD_2Cl_2): δ ^{1}H: 3.06 (tt, $^3J_{HH}$ = 12.6, 3.5 Hz, 1H, CH), 2.35 (br s, 3H, CH_3), 1.99, 1.63 (each m, each 2H, 2-CH_2), 1.84, 1.39 (each m, each 2H, 3-CH_2), 1.74, 1.29 (each m, each 1H, 4-CH_2).

^{13}C{^{1}H} NMR (600 MHz, 299 K, CD_2Cl_2): δ ^{13}C: 142.5, 132.8 (each br, C^{Ar}), 41.9 (CH), 31.0 (2-CH_2), 28.3 (3-CH_2), 26.8 (4-CH_2), 19.3 (CH_3).

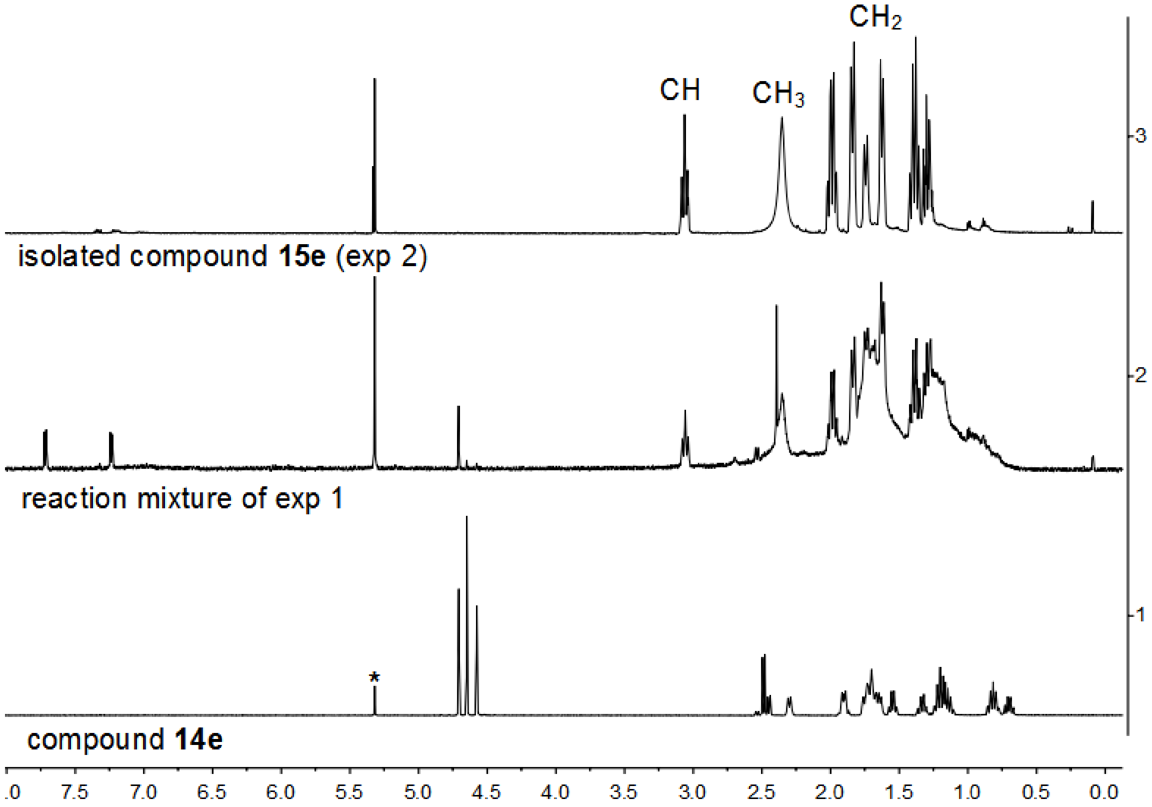

Figure S44. ^{1}H NMR (600 MHz, 299 K, CD_2Cl_2(*))spectra of (1) compound **14e**, (2) the reaction mixture from Experiment 1, and (3) isolated compound **15e** from Experiment 2.

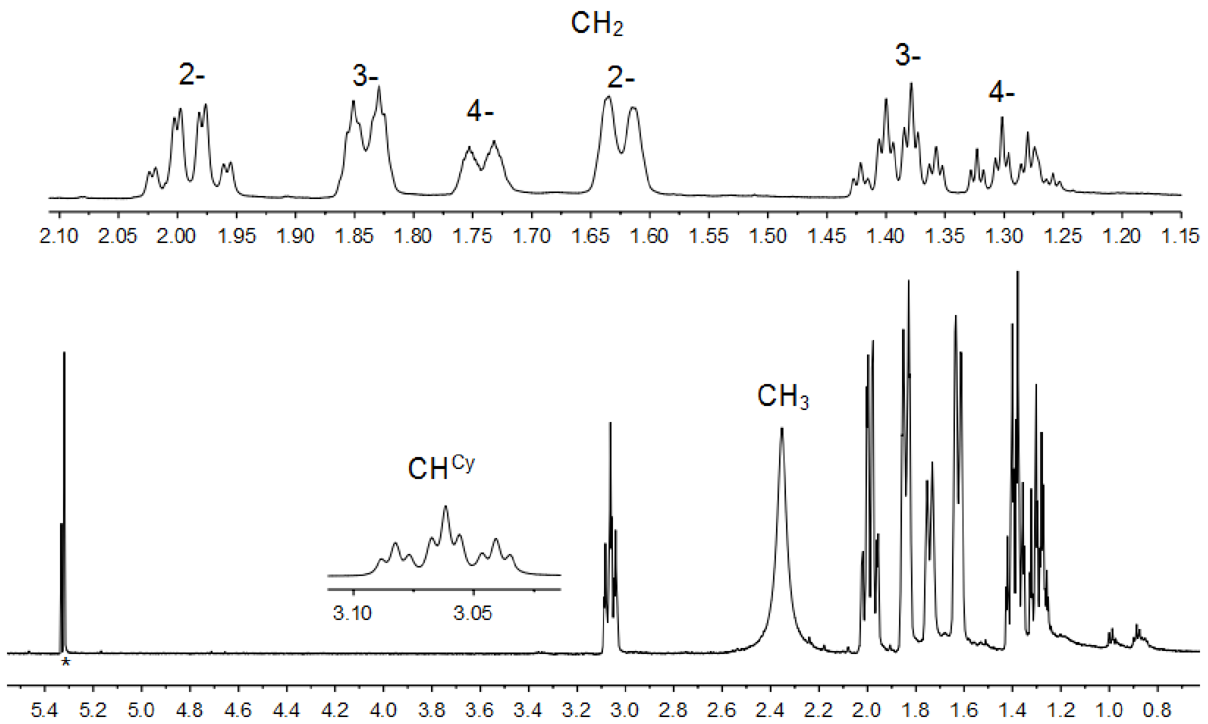

Figure S45. ^{1}H NMR (600 MHz, 299 K, CD_2Cl_2(*)) spectrum of isolated compound **15e** from Experiment 2.

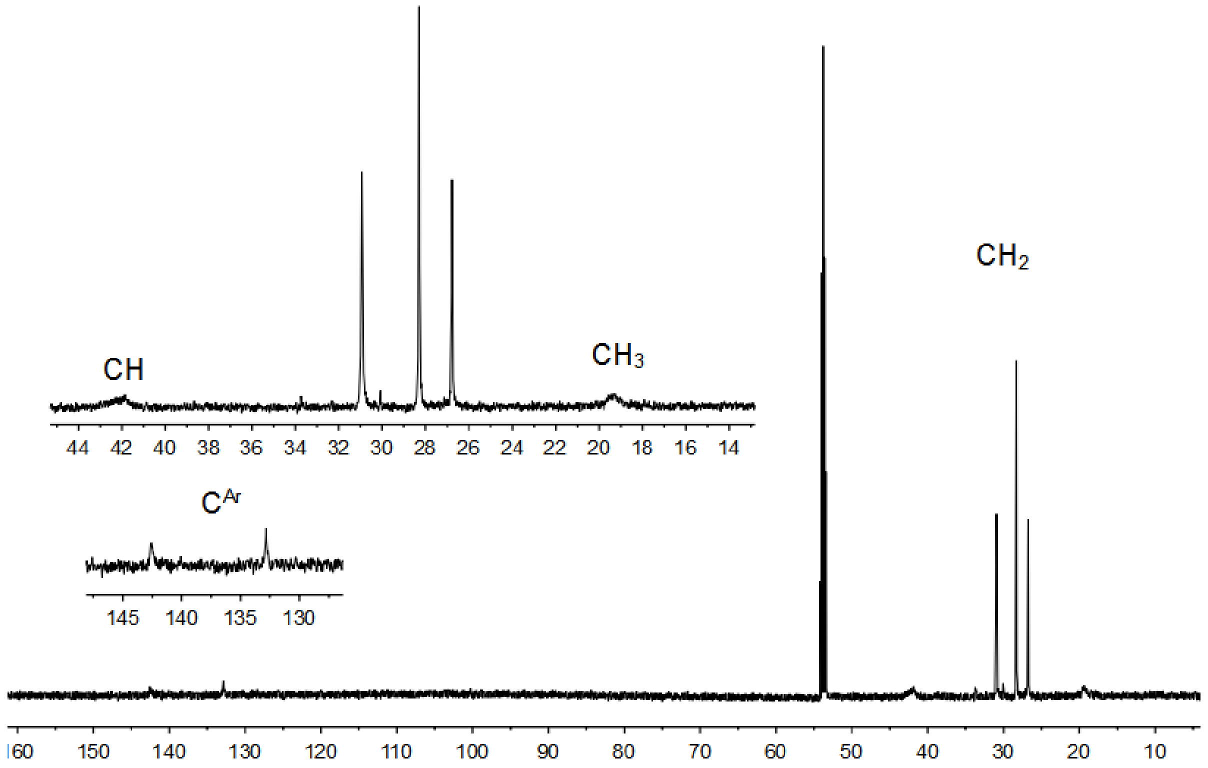

Figure S46. $^{13}C\{^{1}H\}$ NMR (151 MHz, 299 K, CD_2Cl_2) spectrum of isolated compound **15e** from Experiment 2.

X-ray crystal structure analysis of 15e (erk9371): A colorless plate-like specimen of $C_{27}H_{42} \cdot 0.5 \times CH_2Cl_2$, approximate dimensions 0.050 mm x 0.101 mm x 0.224 mm, was used for the X-ray crystallographic analysis. The X-ray intensity data were measured. A total of 1217 frames were collected. The total exposure time was 21.26 hours. The frames were integrated with the Bruker SAINT software package using a wide-frame algorithm. The integration of the data using a triclinic unit cell yielded a total of 25656 reflections to a maximum θ angle of 66.84° (0.84 Å resolution), of which 8246 were independent (average redundancy 3.111, completeness = 98.3%, R_{int} = 5.52%, R_{sig} = 6.09%) and 5834 (70.75%) were greater than $2\sigma(F^2)$. The final cell constants of a = 10.8108(3) Å, b = 12.1099(3) Å, c = 19.1989(5) Å, α = 76.7030(10)°, β = 75.1760(10)°, γ = 87.9660(10)°, volume = 2363.95(11) $Å^3$, are based upon the refinement of the XYZ-centroids of 9965 reflections above 20 σ(I) with 4.890° < 2θ < 133.7°. Data were corrected for absorption effects using the multi-scan method (SADABS). The ratio of minimum to maximum apparent transmission was 0.932. The calculated minimum and maximum transmission coefficients (based on crystal size) are 0.7330 and 0.9300. The structure was solved and refined using the Bruker SHELXTL Software Package, using the space group *P*-1, with Z = 4 for the formula unit, $C_{27}H_{42} \cdot 0.5 \times CH_2Cl_2$. The final anisotropic full-matrix least-squares refinement on F^2 with 639 variables converged at R1 = 6.90%, for the observed data and wR2 = 17.20% for all data. The goodness-of-fit was 1.030. The largest peak in the final difference electron density synthesis was 0.720 $e^-/Å^3$ and the largest hole was -0.554 $e^-/Å^3$ with an RMS deviation of 0.058 $e^-/Å^3$. On the basis of the final model, the calculated density was 1.149 g/cm^3 and F(000), 900 e^-.

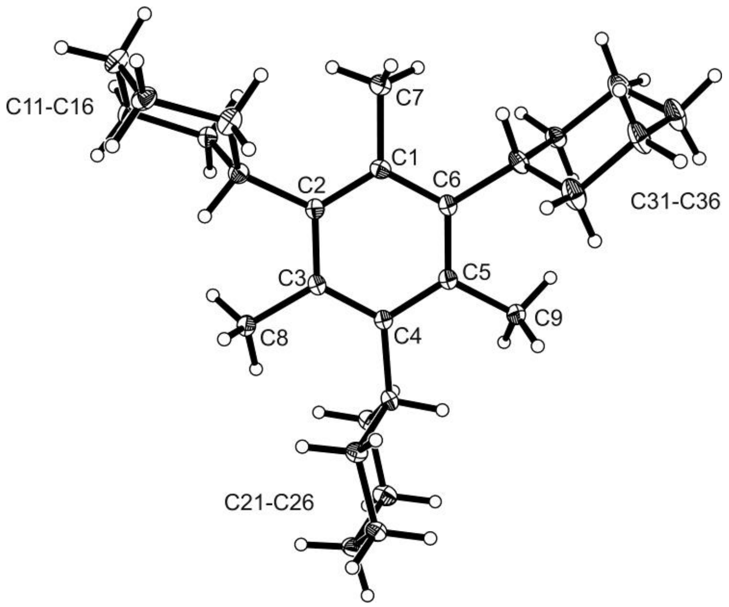

Figure S47 Crystal structure analysis of compound **15e** [only one of two crystallographically independent molecules is shown (mol A); thermal ellipsoids: 30% probability].
